# Pentacosacyclenes: cruciform molecular nanocarbons based on cyclooctatetraene†

**DOI:** 10.1039/d4sc05938g

**Published:** 2024-10-11

**Authors:** Rakesh Kumar, Piotr J. Chmielewski, Tadeusz Lis, Mirosław Czarnecki, Marcin Stępień

**Affiliations:** a Wydział Chemii, Uniwersytet Wrocławski ul. F. Joliot-Curie 14 50-383 Wrocław Poland marcin.stepien@uwr.edu.pl

## Abstract

Pentacosacyclene (PC) and pentacosacyclene tetraimide (PCTI) were obtained in concise syntheses involving radial extension of tridecacyclene. PC is an electron-rich hydrocarbon with a C_88_ π-conjugated framework, whereas PCTI is electron-deficient and contains a C_96_N_4_ core. PC and PCTI both have non-planar saddle-shaped conformations, and PC was found to self-assemble with C_60_ to produce a uniquely structured supramolecular crystalline phase. In solution, PCTI undergoes eight single-electron reductions, while PC exhibits two reversible oxidations and three reversible reduction events. Chemically generated anions of PC and PCTI showcase extended near-infrared to infrared absorptions, with the lowest energy bands observed at >3200 nm for the PCTI monoanion and *ca.* 2800 nm for the PCTI dianion. The electronic and redox properties of pentacosacyclenes can be explained using molecular orbital and valence bond theories as originating from changes in the local aromaticity of five- and eight-membered rings.

## Abstract 2

In pentacosacyclenes, a saddle-shaped cyclooctatetraene ring (orange) undergoes acenaphthylene fusion (green) and rylene extension (yellow). Their properties are further tuned by peripheral decoration with alkyl groups (red) or imide rings (blue).

## Graphical abstract

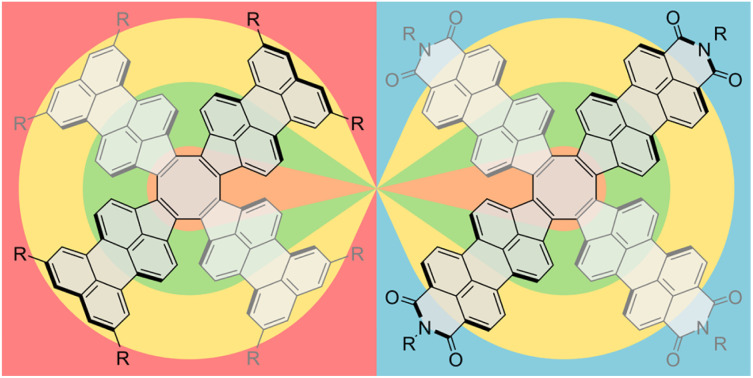

## Introduction

**Fig. 1:**
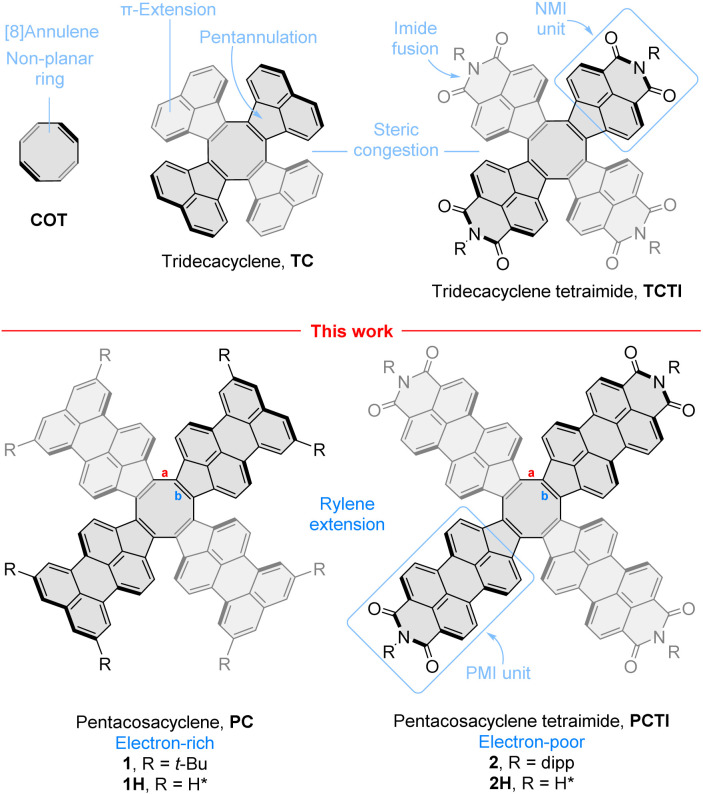
Cyclooctatetraene and its radially π-extended analogues. Bond labeling is indicated for pentacosacyclene derivatives. dipp, 2,6-diisopropylphenyl.

In the chemistry of molecular nanocarbons, non-benzenoid ring fusion, *i.e.* introduction of rings other than six-membered ones, dramatically increases the structural diversity, providing more options for property tuning. This happens not only because of the greater number of available ring frameworks, but also because of the geometric effect of non-benzenoid fusion, which can lead to both positively and negatively curved π systems, depending on the size of the rings introduced. In particular, eight-membered rings, formally derived from 1,3,5,7-cyclooctatetraene (COT, Fig. 1),^1,2^ have served to stabilize the negative curvature in various hetero-^3–10^ and carbocyclic systems,^11–13^ and to promote the formation of reduced anionic states.^14–19^ One π extension route leads *via* the tridecacyclene motif (TC, Fig. 1),^15,20^ the recently discovered higher homologue of decacyclene.^21,22^ Among several π-extended TC derivatives reported to date,^23–27^ our own tridecacyclene tetraimide (TCTI)^26^ is notable for stabilization of eight reduced states up to an octaanion. The resulting anions, particularly the dianion, exhibit strong absorptions in the near-infrared region, making TCTI a potentially useful electrochromic material. This extended redox behavior is promoted by a combination of two key structural features: the introduction of electron withdrawing naphthalenemonoimide (NMI) units,^28^ which are responsible for lowering of virtual MO levels, and pentannulation of the NMI units to the COT ring, which provides for local aromatic stabilization of the anionic states.^26^

While the NMI pentannulation strategy is now relatively well established,^29–35^ systems obtained by multiple pentannulation of higher rylenes have not been similarly explored. The properties of such nanocarbons are interesting in view of the extensive body of work on benzenoid oligoimides.^36–38^ Specifically, larger π systems can lead to better charge delocalization and smaller energy gaps, whereas introduction of additional fused rings is expected to offer additional aromatic stabilization modes unavailable to the smaller NMI counterparts. Because of these possibilities, perylene extension appeared as the next logical step in our investigations. The compactness of the COT core and its ability to sustain non-planar geometries even in relatively large π-systems^9^ encouraged us to consider pentacosacyclene‡‡We propose the trivial name pentacosacyclene, which reflects the presence of 25 fused rings in the core motif of 1 and 2. The name is modeled after decacyclene^21^ and tridecacyclene,^20^ and is notably shorter than possible systematic alternatives. (PC, Fig. 1) and its tetraimide (PCTI) as appropriate targets, combining relative ease of synthesis with good solubility and lack of self-aggregation. PCTI can be considered the higher analogue of TCTI, featuring four perylenemonoimide (PMI) units fused to the COT ring. Herein we describe the synthesis of appropriately functionalized PC (1, R = *t*-Bu) and PCTI (2, R = dipp) and use physical and theoretical analysis to unravel the interplay between rylene length and imide fusion and its effect on the redox and optical properties of π-extended COTs.

## Results and discussion

### Synthesis and structure

**Scheme 1:**
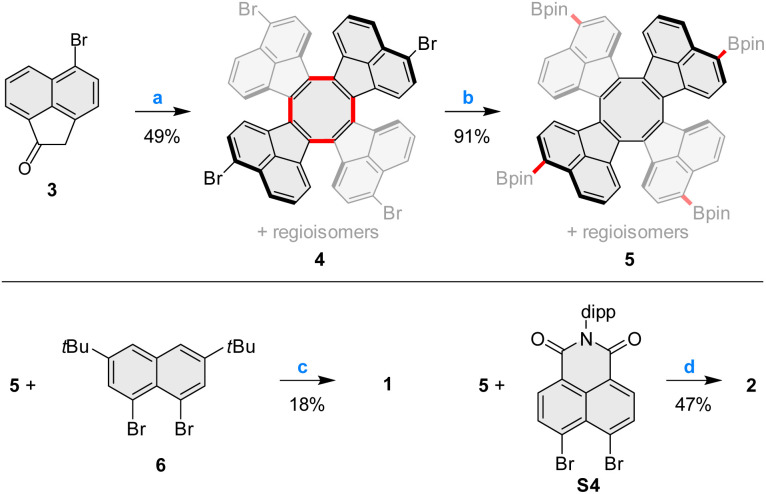
Synthesis of pentacosacyclenes 1–2. Reagents and conditions: (a) TiCl_4_ (6.0 equiv.), *o*-dichlorobenzene, reflux, 3 h; (b) bis(pinacolato)diboron, Pd(dppf)Cl_2_, KOAc, 1,4-dioxane, 110 °C, overnight; (c) Pd_2_(dba)_3_·CHCl_3_, P(*m*-tolyl)_3_, Cs_2_CO_3_, 1-chloronaphthalene, 160 °C, 48 h. (d) Pd_2_(dba)_3_·CHCl_3_, PCy_3_·HBF_4_, Cs_2_CO_3_, 1-chloronaphthalene, 160 °C, 24 h.

Pentacosacyclene derivatives 1 and 2 were obtained according to the procedures shown in Scheme 1. 5-Bromoacenaphthylen-1(2*H*)-one 3, prepared by bromination of acenaphthylen-1(2*H*)-one with *N*-bromosuccinimide, was refluxed with TiCl_4_ in *o*-dichlorobenzene, providing the desired tetrabromo tridecacyclene 4 in 49% yield. Formation of other cyclic homologues of 4 (including decacyclene) was not observed under these conditions. 4 was obtained as a mixture of positional isomers;^27^ however, separation of the isomers was unnecessary, since all of them would converge to the same final product. Borylation of 4 with bis(pinacolato)diboron under Miyaura conditions efficiently yielded the corresponding mixture of tetraboronic esters 5. Fourfold annulative coupling between 5 and the dibromonaphthalene 6, performed under conditions developed by Würthner *et al.*,^39^ produced compound 1 in a relatively low yield (18%), which is however typical for multiple [3 + 3] annulations of this kind. A similar reaction^40^ involving 5 and the known building block S4 furnished the tetraimide 2 in a significantly better yield of 47%. Both 1 and 2 were unambiguously characterized using NMR spectroscopy and mass spectrometry (for complete synthetic and analytical details, see the ESI†).

**Fig. 2:**
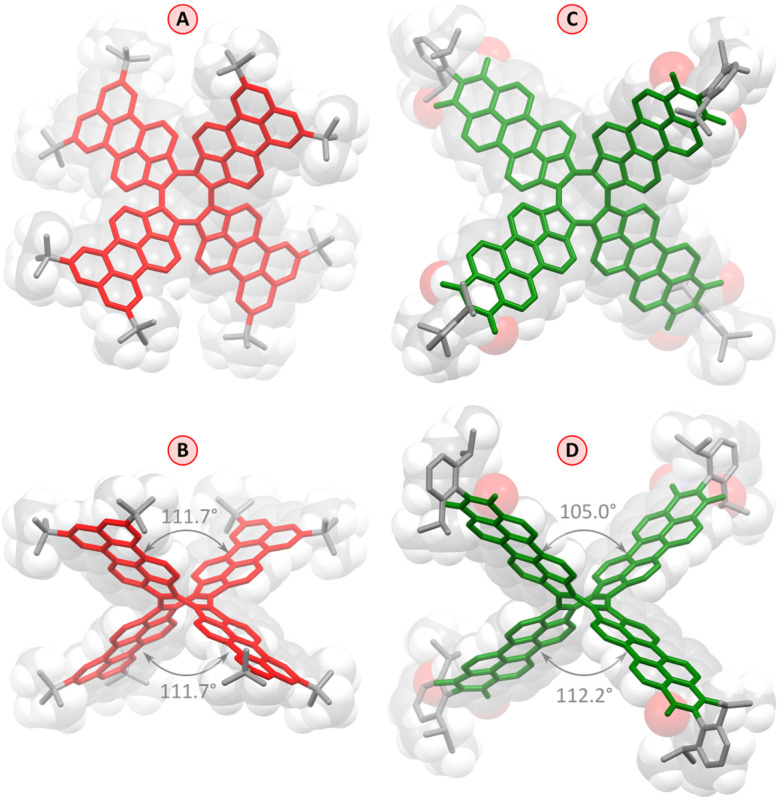
Molecular structure of 1 (red) and 2 (green) determined by X-ray diffraction analyses (solvent molecules and hydrogens are omitted for clarity). Axial (A and C) and meridional views (B and D) are shown relative to the fourfold molecular axis. Splay angles of 1 and 2 between nonadjacent perylene units are indicated in panels (B) and (D), respectively.

Slow evaporation of an *n*-hexane solution of 1 yielded red-colored single crystals suitable for X-ray diffraction (XRD) analysis, which unambiguously confirmed the pure hydrocarbon structure of 1 (C_120_H_104_, Fig. 2). The crystals were tetragonal (*I*4_1_/*acd* space group), with the PC molecule located on a 4̄ special position. The cruciform aromatic core of PC had the expected shape of a deep saddle, with the symmetrically equivalent splay angles of 111.7° (*vs.* 90.8° and 114.8° in TCTI^26^ and 103–105° in TC^20^). The bond length pattern in 1 is consistent with localization of the COT double bonds in the five membered rings, and essentially unperturbed π conjugation in the perylene subunits. The presence of *t*-Bu groups in the structure is apparently sufficient to prevent any π-stacking interactions in the crystal.

The red–violet crystals obtained by slow vapor diffusion of *n*-hexane into a solution of 2 in toluene were found to contain a toluene solvate, 2·14C_7_H_8_. The level of solvation in this structure is remarkably high, a feature previously observed by us in other NMI-dipp derivatives.^29,30,41^ As in these earlier instances, the PC molecules for a loose network stabilized by weak interactions involving dipp groups and edges of the PMI units (formal void volume of *ca.* 52%, as estimated using contact surface calculations), with the resulting channels filled with toluene molecules. The geometry of individual molecules of 2 is similar to that determined for 1, although the two splay angles are no longer equal, and the individual PMI units show somewhat stronger deviations from planarity. It should, however, be noted that the molecule of 2 has a larger enclosing van der Waals radius than 1 (18.3 Å *vs.* 14.2 Å), and may be more susceptible to distortions caused by packing forces in the crystal.

**Fig. 3:**
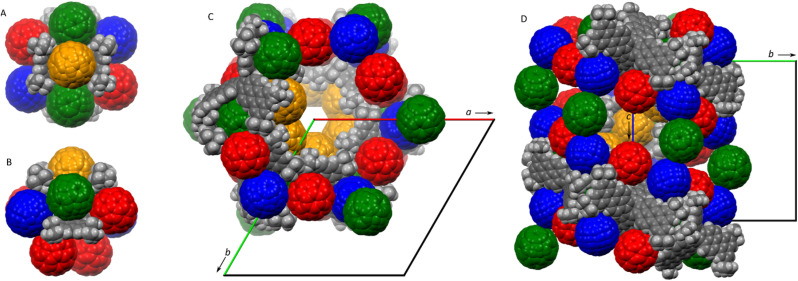
Partial packing diagrams of the 1·5C_60_·*n*C_7_H_8_ phase. (A and B) The molecule of 1 and its nearest fullerene neighbors viewed along the twofold symmetry axis (A) and in a perpendicular direction (B). (C and D) Packing diagrams along the *c* axis (C) and a* axis (D), showing the structure of the 6_5_ channel. 1 is shown in gray whereas the four symmetry-independent fullerene molecules are in red, green, blue, and orange, respectively. Refined toluene molecules and disordered positions of *t*-Bu groups are removed for clarity.

Given its electron-rich, negatively curved π surface, 1 was envisaged as a potential host for fullerenes.^41–44^ No evidence for self-assembly between 1 and C_60_ was found in toluene-*d*_8_ solution using ^1^H NMR spectroscopy. However, compound 1 co-crystallized with buckminsterfullerene and toluene, to produce a supramolecular phase with a composition of 1·5C_60_·*n*C_7_H_8_. A single-crystal XRD analysis showed that the phase had a hexagonal symmetry, and was assigned to the enantiomorphic *P*6_5_22 space group.§§The *P*6_5_22 space group was chosen arbitrarily over *P*6_1_22. The chirality of the specimen could not be determined from diffraction data. The latter finding is remarkable, because macroscopic chirality is produced by supramolecular co-assembly of achiral components.^45,46^ The crystal contains one symmetry-independent molecule of 1, which is located on a twofold symmetry axis (gray, Fig. 3). Unusually, the lattice contains four types of symmetry-independent C_60_ molecules, one of which is located on a general position (red), whereas the other three are disordered on twofold symmetry axes (green, blue, and orange). The structure contains extended cavities corresponding to a fractional cell volume of 35%, which are filled with toluene molecules. Most of these molecules are highly disordered but partial refinement was nevertheless achieved yielding a total refined occupancy of *n* = 7.2.

Each molecule of 1 is surrounded by nine fullerenes, which form close contacts with the pentacosacyclene surface and among themselves (Fig. 3A and B). In one of these interactions, the C_60_ molecule (shown in orange) is clasped between two opposite perylene units, leading to a notable decrease of the splay angle (73°, *vs.* 127.9° on the opposite side of the PC molecule). Remarkably, the orange C_60_ molecules define extended helical channels along the 6_5_ screw axes running along the crystallographic *c* direction (Fig. 3C). These channels contain recesses extending laterally in the *ab* plane (visible between pairs of red fullerenes in Fig. 3D). The packing motif is notably more complex than that described for a TC–C_60_ phase,^20^ showcasing the ability of negatively curved aromatics to promote the formation of structurally non-trivial supramolecular solids.

### Electronic and redox properties

Simultaneous availability of PC and PCTI derivatives, 1 and 2, respectively, offers a chance of analyzing the individual effects of radial rylene extension and imide fusion on the electronic properties of these COT derivatives. The electronic absorption spectra of 1 and 2 (Fig. 5A and B, black traces) are qualitatively similar, featuring one structured band in the visible region (*λ*^abs^_max_ = 561 nm and 573 nm, respectively) and a weak absorption tail extending up to 900–1000 nm. Absorption spectra recorded in a range of non-polar and polar solvents showed that the two compounds are only weakly solvatochromic (see ESI Fig. S5 and S14†), which is noteworthy given the dipolar character of the perylenemonoimide fragments present in PCTI.

**Fig. 4:**
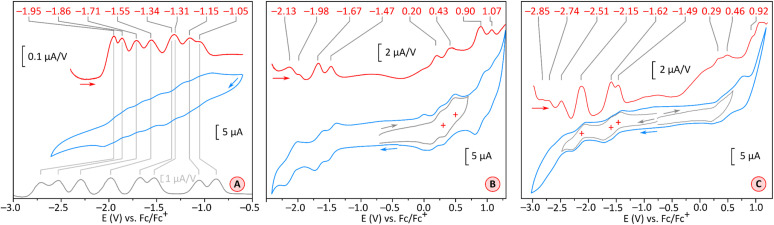
Cyclic voltammograms (CV, blue traces) and differential pulse voltammograms (DPV, red traces) recorded for compound 2 (THF, [Bu_4_N]PF_6_, glassy-carbon electrode, 100 mV s^−1^, panel (A)) and for compound 1 (in DCM, [Bu_4_N]PF_6_, glassy-carbon electrode, 100 mV s^−1^, panel (B); and in THF, [Bu_4_N]PF_6_, glassy-carbon electrode, 100 mV s^−1^, panel (C)). The gray trace in panel (A) contains the reported DPV of TCTI.^26^

As anticipated, tetraimide 2 revealed its multivalent nature in electrochemical experiments (Fig. 4A, S19 and S20†). It undergoes multiple reversible reductions in the −1.05 to −1.95 V potential range (*vs.* Fc^+^/Fc, Fig. 4A). Up to eight one-electron events can be found in this voltage region, in line with chronocoulometric experiments (Fig. S21–S25†). Thus, PCTI is somewhat less susceptible to the first two reductions than TCTI^26^ (*E*_red1_ = −0.88 V, *E*_red2_ = −1.06 V) but the subsequent six events take place at progressively higher relative potentials, with the difference between *E*_red8_ values being particularly large (−2.71 V for TCTI). In fact, at the potential of −2.00 V, TCTI remains at the tetraanion level, whereas PCTI is already completely reduced. Two irreversible oxidation events at *ca.* 0.66 V and 0.92 V were also observed for 2, implying a further decrease of the electrochemical energy gap (*ca.* 1.71 eV) relative to those of TCTI (*ca.* 1.89 eV)^26^ and TC (2.11 eV).^20^

In comparison, the electron-rich PC derivative 1 showed four one-electron oxidations (in DCM, Fig. 4B and S15†), two of which, at 0.20 V and 0.43 V, were reversible. The low-potential events were better defined in THF (Fig. 4C, S16 and S17†): the first two reductions occurred at −1.49 V and −1.62 V, *i.e.* at notably lower potentials than the corresponding reductions of 2. The effect of imide fusion is however weaker in the PCTI/PC pair, for which the shift of the first reduction potential is −0.44 V, in comparison with the TCTI/TC pair for which the corresponding shift is *ca.* −0.79 V.^20,26^ The combined voltammetric data suggest that the apparent electrochemical energy gap of 1 (1.67–1.78 eV) is comparable with that of 2 (*ca.* 1.71 eV), with both values being slightly smaller than the reported gaps of TCTI (*ca.* 1.89 eV)^26^ and TC (*ca.* 2.11 eV).^20^

**Fig. 5:**
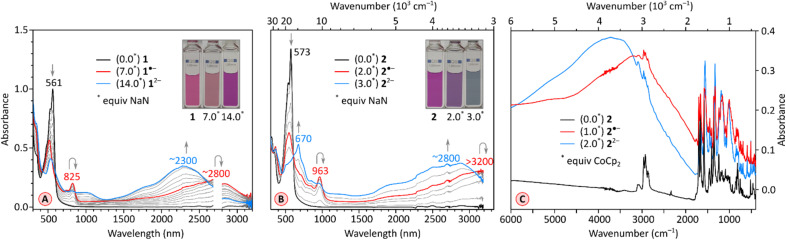
(A) Stepwise reduction of 1 (0.154 mM in THF) with sodium naphthalenide (NaN, 1.54 mM in THF). The red and blue traces correspond to the maximum concentration of [1˙^−^ and [1]^2-^, respectively. (B) Stepwise reduction of 2 (0.10 mM in THF) with sodium naphthalenide (NaN, 1.0 mM in THF) in the presence of 15 crown 5 ether (100 equiv.). The red and blue traces correspond to the maximum concentration of [2]˙^−^ and [2]^2−^, respectively. (C) IR spectra in a KBr pellet of reduction of 2 with cobaltocene (in THF). The black, red and blue traces correspond to the maximum concentration of 2, [2]˙^−^ and [2]^2−^, respectively.

The chemical reduction of 1 could be effected with sodium naphthalenide (NaN), which was employed in THF, in the presence of 15-crown-5 ether (Fig. 5A). The spectra of the resulting [1]˙^−^ and [1]^2−^ qualitatively resemble those previously reported for TCTI,^26^ but are notably more red-shifted, with absorption maxima at *ca.* 2800 nm and 2300 nm, respectively. For 2, which is easier to reduce than 1, the first two reductions were accessible with both cobaltocene (CoCp_2_, *E*°′ = −1.3 V,^47^ Fig. S7†), and sodium naphthalenide (NaN, Fig. 5B), yielding similar though not identical spectral changes. Notably, no isosbestic points were observed in the CoCp_2_ titration, suggesting the coexistence of [2]˙^−^ and [2]^2−^ in solution, in line with the small difference of *E*_red1_ and *E*_red2_ potentials (0.10 V). The dianion produced in the reaction with CoCp_2_ was subsequently reoxidized back to the neutral species using diiodine. A better separation of the two reduction events was seen when the more potent reagent NaN was used, enabling clear identification of the radical anion [2]˙^−^ and the dianion [2]^2−^ in the UV-vis-NIR spectra. The NIR bands of the reduced states, [2]˙^−^ and [2]^2−^, are even more red-shifted, with maxima at >3200 nm and at *ca.* 2800 nm, respectively. These two values correspond to a dramatic bathochromic shift when compared with the respective absorptions of [TCTI]˙^−^ and [TCTI]^2−^ (2084 nm and 1692 nm, respectively). Using these values, it can be estimated that on going from TCTI to PCTI the optical HOMO–LUMO gap of the monoanion and dianion is reduced respectively by ≥0.21 eV and by ≈0.29 eV.

π-Electronic transitions rarely extend into the infrared region occupied by the vibrational spectrum (*λ* > 3000 nm).^48,49^ To reveal the full extent of electronic absorptions of the PCTI anions, we recorded the IR spectra of [2]˙^−^ and [2]^2−^, as well as the spectrum of the neutral 2 (Fig. 5C). The anion samples were generated by reduction with cobaltocene, in the inert atmosphere of a glovebox; the solvents were removed and the resulting solids were pressed into KBr pellets. The absorption maxima of [2]˙^−^ and [2]^2−^ were located at approximately 3000 cm^−1^ and 3750 cm^−1^, respectively, with both bands extending beyond 2000 cm^−1^. In each case, the overlap with the vibrational spectrum leads to considerable suppression of C–H stretching bands, possibly reflecting a Fano resonance between the discrete vibrational transitions and the electronic absorption continuum.^48,49^

**Fig. 6:**
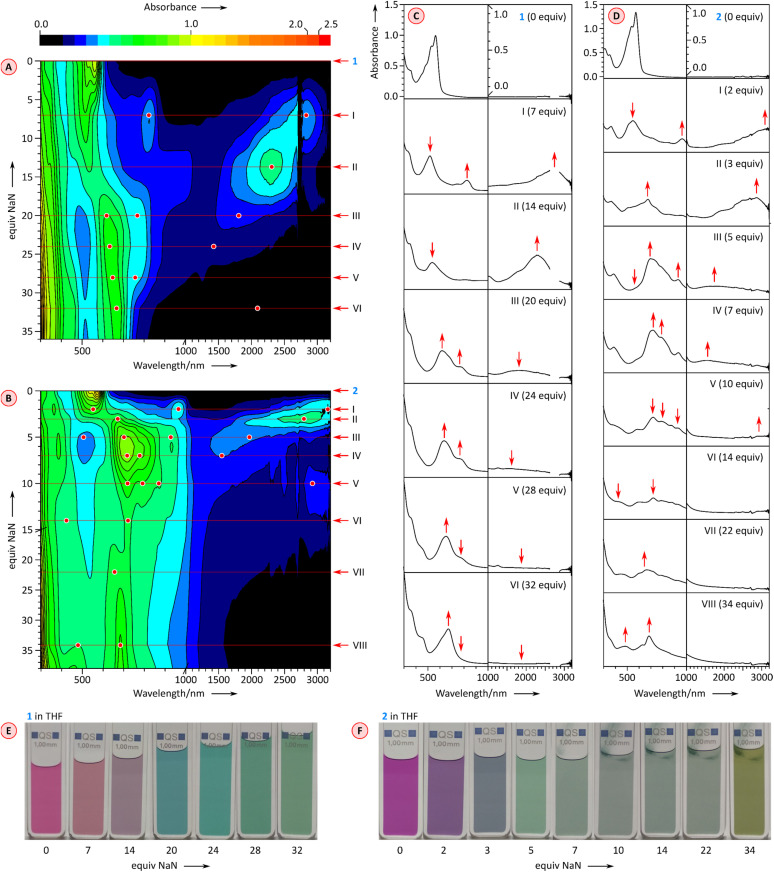
Chemical reduction of 1 (0.154 mM in THF) and 2 (0.1 mM in THF) with sodium naphthalenide (NaN, nominal equivalents corresponding to the initial concentration of naphthalene) in the presence of 15-crown-5 (100 equiv. added to the initial solution). (A and B) 2D maps showing the changes of UV-vis-NIR spectra as a function of added NaN. Key titration stages are labeled with Roman numerals. Spectral features of interest are indicated with red dots and arrows. (C and D) Cross-sections of the 2D maps at key titration stages. (E and F) Color changes observed during titration.

Further reduction of 1 and 2 was carried out using larger amounts of NaN and monitored with UV-vis-NIR spectroscopy (Fig. 6). Depending on the number of reduced forms and the relative reduction potentials, the emergence of higher anionic states can be observed with variable precision in such experiments.^26,29,30^ After the initial reduction to the mono-dianion (Fig. 6A and C, stages I and II), compound 1 produces further spectral changes in the NIR region when treated with further equivalents of NaN, but the changes are relatively poorly resolved. Overall, four additional stages could be discerned in the titration, which could in principle correspond to a reduction to at least the hexaanion [1]^6−^. However, the ultimate stage contains no significant absorptions in the NIR range, a feature previously found to correspond to fully reduced octaanion [TCTI]^8−^.^26^ Since [1]^8−^ is also expected to have a larger energy gap than the intermediate anionic states, stage VI may actually correspond to the octaanion or, given the limited reversibility of reductions observed electrochemically, to other reduction products. The chemical reduction of 2 (Fig. 6B and D) proceeded *via* the previously observed [2]˙^−^ and [2]^2−^, which could be generated using a relatively smaller amount of added NaN that the corresponding anions of 1. Further spectral changes appeared to correspond to six consecutive reductions and the ultimate formation of the octaanion [2]^8−^, displaying no NIR absorption maxima. The latter species could be reverted to the neutral 2 through reoxidation achieved by the addition of diiodine in tetrahydrofuran.

**Fig. 7:**
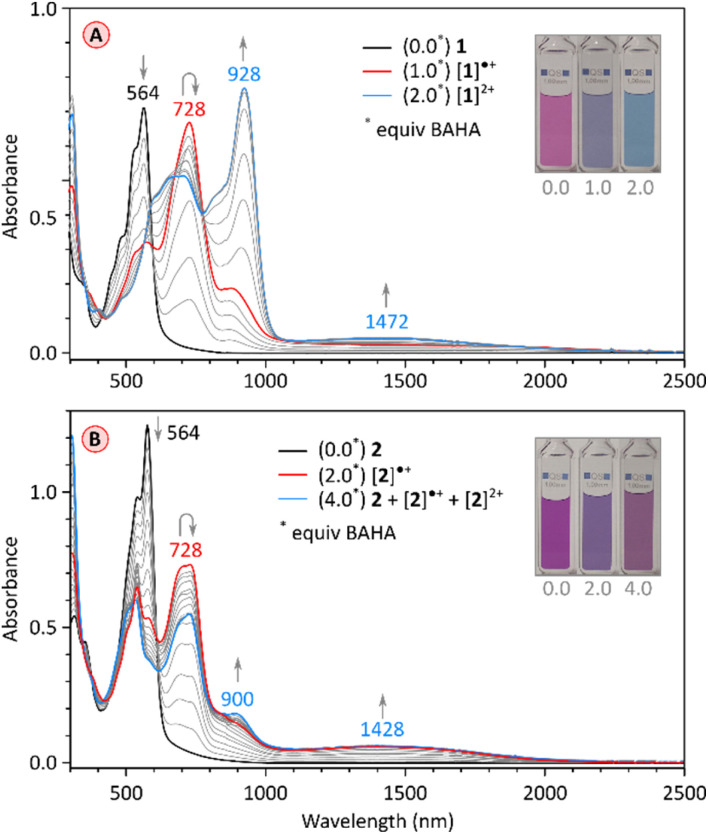
(A) Stepwise oxidation of 1 (0.108 mM in CH_2_Cl_2_) with tris(4-bromophenyl)ammoniumyl hexachloroantimonate (BAHA, 1.08 mM in CH_2_Cl_2_). The red and blue traces correspond to the maximum concentration of [1]˙^+^ and [1]^2+^, respectively. (B) Stepwise oxidation of 2 (0.1 mM in CH_2_Cl_2_) with tris(4-bromophenyl)ammoniumyl hexachloroantimonate (BAHA, 1.0 mM in CH_2_Cl_2_). The red trace corresponds to the maximum concentration of [2]˙^+^.

Chemical oxidation of the electron-rich PC derivative 1 occurred cleanly with stoichiometric amounts of tris(4-bromophenyl)ammoniumyl hexachloroantimonate (BAHA, *E*°′ = 0.7 V in DCM,^50^Fig. 7A and S2†). Two cationic states were observed, [1]˙^+^ and [1]^2+^, characterized by principal absorption maxima at 728 nm and 928 nm, respectively. The radical cation featured additional tailing absorption in the NIR range, which evolved into a weak maximum at 1472 nm in the dication. Isosbestic points were identified for both oxidation steps, consistent with the relatively large difference between the *E*_ox1_ and *E*_ox2_ potentials (0.23 V). [1]^2+^ could be reduced back to the neutral 1 by exposure to hydrazine hydrate. The initial oxidation of tetraimide 2 with 2 equiv. of BAHA produced the corresponding monocation [2]˙^+^, identifiable by the similarity of its spectrum to that of [1]˙^+^ (Fig. 7B and S13†). Subsequent addition of BAHA did not however lead to the formation of the dication, which is apparently not accessible under these experimental conditions (*E*_ox1_ = 0.66 and *E*_ox2_ = 0.92, Fig. S18†). These experiments nevertheless show that both pentacosacyclenes 1 and 2 provide better stabilization of oxidized species than the respective tridecacyclene derivatives TC (*E*_ox1_ = 0.57 onset, non-reversible)^20^ and TCTI (*E*_ox1_ = 1.01 V, non-reversible).^26^

### Electronic structure and aromaticity

**Fig. 8:**
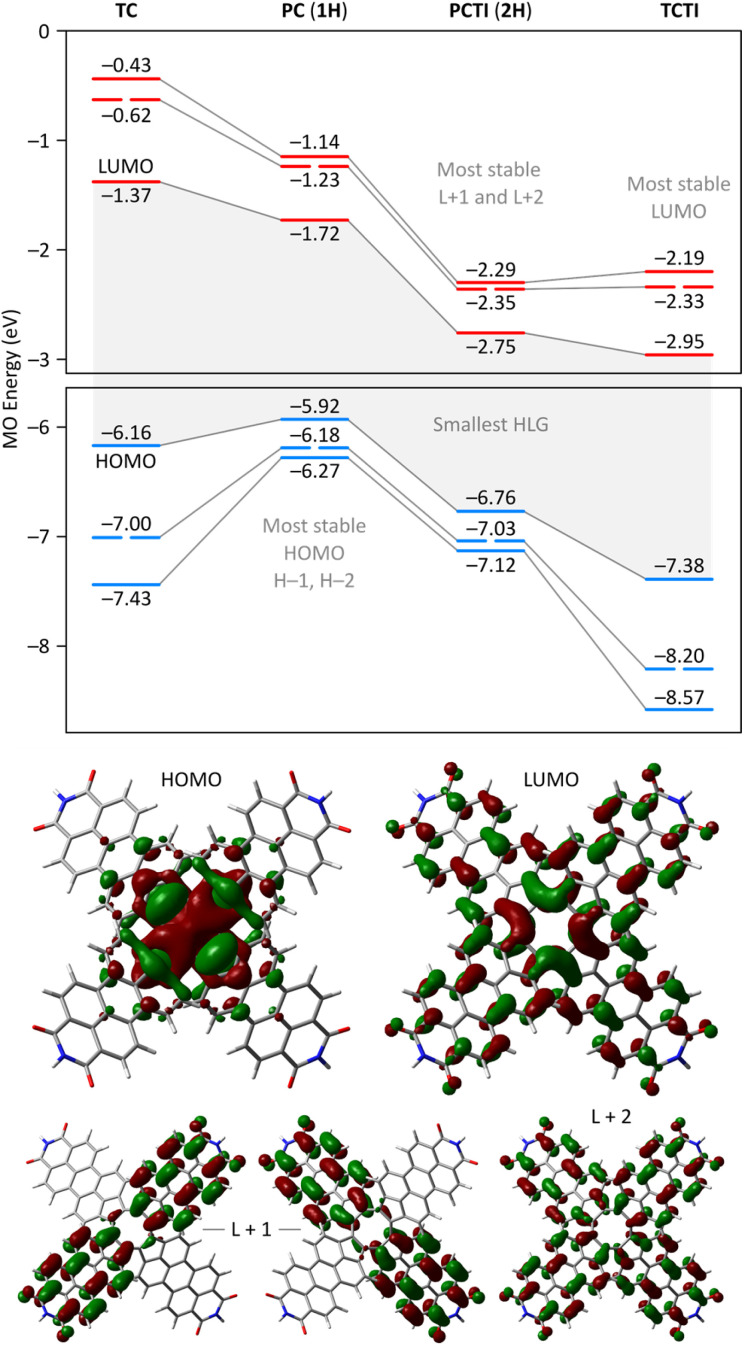
Top: Kohn–Sham molecular orbital diagrams from the substituent-free tridecadecacyclenes and pentacosacyclenes 1H and 2H (GD3BJ-CAM-B3LYP/6-31G(d,p)). Gray lines connect the corresponding MO levels. Bottom: selected MO amplitudes for 2H.

A theoretical insight into the electronic properties of the perylene-fused COTs 1 and 2 was obtained from density functional theory (DFT) calculations carried out for substituent-free models 1H and 2H (each with R = H, *cf.*Fig. 1) at the most relevant oxidation levels of both systems. A qualitatively similar pattern of Kohn–Sham molecular orbitals (MOs) was found for the neutral singlets ^1^1H and ^1^2H: both systems feature a non-degenerate HOMO that is predominantly localized on the COT ring (Fig. 8). The LUMO is also singly degenerate, and is delocalized over the entire π framework, with constructive interactions between adjacent five-membered rings. In both systems, the H−1 and L+1 levels have double degeneracy, while the H−2 and L+2 levels are nondegenerate. This key range of frontier orbitals has very similar MO amplitudes in 1H and 2H, indicating that the presence of imide functionalities does not lead to qualitative changes in the electronic structure. However, in the tetraimide, all of these levels are shifted to lower energies (by more than 1 eV in the case of virtual orbitals), in line with the strongly electron-withdrawing character of the imide groups. Consequently, 2H has four low-lying virtual levels, thus justifying the ability of 2 to undergo eight one-electron reductions. The higher lying virtual levels in 1H are consistent with its lower reduction potentials and, potentially, the narrower range of reduced states attainable by 1. Importantly, in comparison with TCTI, the LUMO of 2H is less stabilized by 0.20 eV, whereas the L+1 and L+2 levels have somewhat lower energies. This observation nicely justifies the differences in relative reduction potentials between 2 and TCTI (*vide supra*). Apparently, the relatively high-lying LUMO of 2H reflects the weaker effect of imide fusion in the radially extended π system of PCTI.

**Fig. 9:**
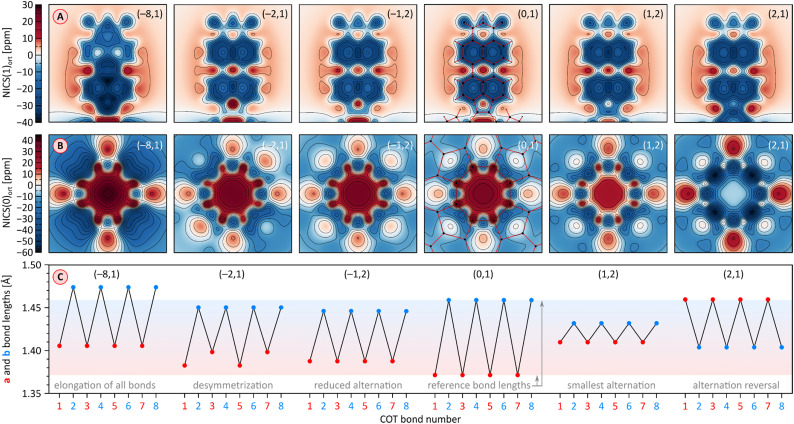
(A and B) Nucleus-independent chemical shift (NICS) maps evaluated for 2H at the GIAO/GD3BJ-CAM-B3LYP/6-31G(d,p) level of theory. NICS(*z*)_ort_ are the negative values of the magnetic shielding component in the direction perpendicular to the reference plane, calculated at an offset of *z* Å relative to that plane. The NICS maps are calculated for PMI units (panel A), at an offset of *z* = 1 Å in the direction approaching the fourfold axis, and for the COT rings (panel B) in the direction of the fourfold axis at the geometric center of the ring (*z* = 0 Å). The PMI projection of ^1^[2H]^2−^ contains one of the subunits with a shorter a bond. Projections of the molecular framework of 2H are only shown for the neutral singlet state. (C) Variation of a and b bond lengths in the COT ring of 2H as a function of its oxidation state. Bonds 1–8 are numbered consecutively starting with an a-type bond (*cf.*Fig. 1). Reference bond lengths of the neutral singlet state of 2H are indicated with a shaded horizontal bar. The (*m*,*n*) pairs shown in all subpanels correspond to the charge (*m*) and multiplicity (*n*) of specific electronic states of ^*n*^[2H]^*m*^.

2H and 1H display similar, yet remarkable variation of bond lengths in the COT ring as a function of the oxidation level. These changes are complemented by modulation of local magnetic characteristics evident in 2D NICS maps (Fig. 9). As illustrated for 2H in Fig. 9C, the neutral state features a tetraene-like bonding pattern, with the shorter bonds of the COT unit (1.371 Å) embedded in the five-membered rings, and the longer bonds (1.459 Å) linking the cyclopenta[*cd*]perylene moieties. The interior of the COT ring in ^1^2H is noticeably shielded (Fig. 9B), somewhat more strongly that in the parent COT molecule, which also features stronger bond alternation (1.334/1.472 Å). While these features are consistent with the emergence of weak antiaromaticity in the COT ring of ^1^2H, the observed effect may also be caused by other factors, such as superposition of outer ring currents.

**Fig. 10:**
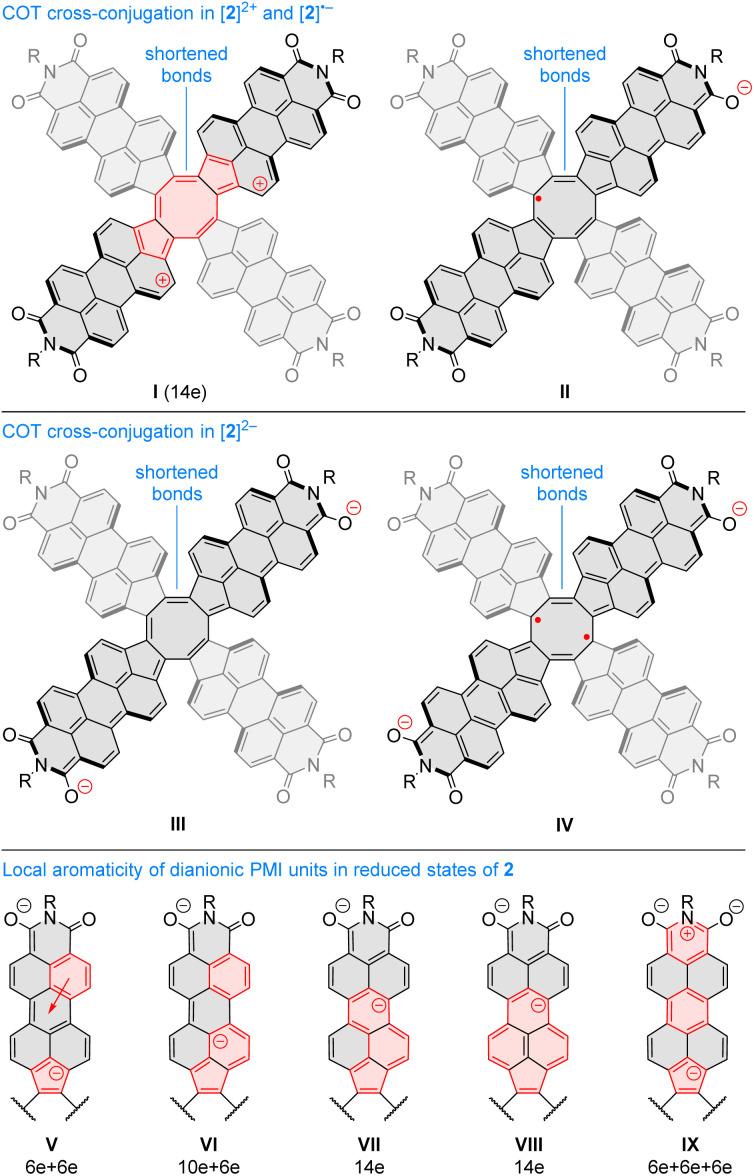
(I–IV) Examples of resonance contributors increasing inter-subunit couplings in PCTI ions. (V–IX) Resonance contributors explaining the local diatropicity of the PCTI octaanion. Annulenoid conjugation pathways are indicated in red.

Oxidation to the radical cation ^2^[2H]^+^ produces the smallest bond length alternation and a reduced shielding inside the COT ring. Interestingly, these characteristics are intermediate between those of the neutral 2H and the dication ^1^[2H]^2+^. The latter state is notable for a reversal of bond-length alternation, the a bonds (1, 3, 5, and 7, red labels in Fig. 9C) being longer than the b bonds (1.460 Å *vs.* 1.404 Å). Additionally, the eight- and five-membered rings in ^1^[2H]^2+^ become notably diatropic, a unique feature across the whole range of states shown. The diatropicity of the COT ring could be rationalized by invoking the aromaticity of the cyclooctatetraene dication [COT]^2+^. However, the latter species is planar and displays full equalization of bond lengths (1.404 Å), in contrast to the PCTI dication. The increase of aromaticity seen in ^1^[2H]^2+^ may originate from additional [10]annulenoid resonance contributions such as I (Fig. 10), which would also explain the unexpected alternation reversal.

Radical anions ^2^[2H]^−^ and ^2^[1H]^−^ are notable for reduced bond alternation in the COT rings. In particular, a slight shortening of the b bonds indicates a stronger interaction between subunits. This feature is in line with the constructive overlap of amplitudes at the b bonds in the LUMO levels of 1H and 2H, and can be further explained by considering resonance contributors of type II (Fig. 10), in which the placement of the radical center in the COT ring results in an increased double bond character of the b bonds. In contrast to ^1^[2H]^2+^ and ^1^[1H]^2+^, which are both closed-shell species, the corresponding dianions, ^1^[2H]^2−^ and ^1^[1H]^2−^, are predicted to be ground-state open-shell singlets, with low lying-triplet states (Δ*E*_ST_ < 2 kcal mol^−1^). In addition, the dianions show reduced symmetries (*C*_2v_ rather than *D*_2d_), with two types of a bonds of unequal length. With a non-degenerate LUMO, neither ^1^[2H]^2−^ nor ^1^[1H]^2−^ should be susceptible to a Jahn–Teller (JT) distortion. It can be proposed that the geometrical distortion and configuration mixing in the dianions occur *via* a pseudo-JT effect originating from partial population of the low-lying L+1 level, which splits in the *C*_2v_ symmetry. This assumption is confirmed by localization of spin density on two opposite PMI units containing longer a bonds. The conjugation pattern found in the dianion may be seen as a superposition of closed-shell contributions such as III (Fig. 10), which cross-conjugate opposite PMI subunits, and open-shell contributions such as IV, which is directly derived from the radical anion configuration II.

The octaanion ^1^[2H]^8−^ has longer b bonds (1.474 Å) than any of the oxidation levels described above. This lengthening suggests that the subunits are effectively decoupled, similarly to what has been reported for the TCTI octaanion.^26^ Subunits of the PCTI octaanion show a striking increase of aromaticity, which differs qualitatively from other oxidation levels, which mostly feature perylene-like aromaticity, decomposable into two 10-electron segments. In ^1^[2H]^8−^ the most diatropic region encompasses the five membered ring and its three nearest six-membered neighbors. In addition, an increase of diatropicity is seen in the imide ring. This behavior can be interpreted by assuming that each of the subunits bears two negative charges, one of which resides closer to the five membered ring whereas the other is predominantly associated with the imide unit. With this assumption, it is possible to draw a number of valence structures V–IX, with clearly discernible local aromaticity (Fig. 10). In comparison with the TCTI octaanion, which was stabilized primarily by cyclopentadienide and indenide contributions analogous respectively to V and VI,^26^^1^[2H]^8−^ benefits additionally from the 14-electron aromaticity of cyclopenta[*a*]naphthalenide (VII) and cyclopenta[*cd*]phenalenide (VIII) substructures. Interestingly, charge-separated pyridinium structures such as IX can be used to rationalize the diatropicity of the imide ring seen in ^1^[2H]^8−^.

## Conclusions

The chemistry of pentacosacyclenes described in this work offers a deeper insight into the individual effects of radial π-extension and imide fusion on the properties of non-benzenoid aromatics. The extension leads to smaller energy gaps and a denser distribution of frontier orbital energies. The imide rings, in turn, introduce additional stabilization of both filled and virtual levels, which is however smaller than in the corresponding tridecacyclene tetraimide. These changes of MO energies result in a more compressed range of reduction potentials, increased accessibility of oxidized states, and in remarkably small energy gaps in the mono- and dications. The latter feature may potentially be useful for developing infrared electrochromic materials. The embedded cyclooctatetraene ring has a defining effect on the properties of pentacosacyclenes, by introducing nonplanarity and promoting electronic communication between subunits in the charged states. The COT ring offers a strain-free strategy of developing negatively curved π surfaces, which can be exploited for supramolecular interactions with spherical guests, as demonstrated by the intricate crystalline phase formed by C_60_ and pentacosacyclene, wherein spontaneous chirality resolution is achieved by combining two achiral components. Investigations of other π-aromatic oligoimides are ongoing in our laboratory.

## Data availability

The data supporting this article have been included as part of the ESI.†

## Author contributions

R. K. and M. S. designed the experiments. R. K. performed all the synthetic experiments and characterization. T. L. performed X-ray crystallographic analyses. P. J. C. performed electrochemical analyses. M. C. and R. K. performed vibrational spectroscopy measurements. M. S. performed theoretical calculations. M. S. wrote the manuscript with contributions from other authors.

## Conflicts of interest

There are no conflicts to declare.

## Supplementary Material

SC-OLF-D4SC05938G-s001

SC-OLF-D4SC05938G-s002

SC-OLF-D4SC05938G-s003
