# Supplementary material for: Pentacosacyclenes: cruciform molecular nanocarbons based on cyclooctatetraene
Source: Chem Sci. 2024 Oct 11;15(44):18640–9. doi: 10.1039/d4sc05938g (PMC11499954; doi:10.1039/d4sc05938g)
Supplement: SC-015-D4SC05938G-s002 [file SC-015-D4SC05938G-s002.pdf]

Supplementary Information (SI) for Chemical Science.
This journal is © The Royal Society of Chemistry 2024

Pentacosacyclenes: Cruciform Molecular Nanocarbons Based on Cyclooctatetraene

Supplementary Information

by Rakesh Kumar, Piotr J. Chmielewski, Tadeusz Lis, Mirosław Czarnecki, and Marcin Stępień*

Table of Contents

Experimental 2
Synthesis 6
Additional Schemes 12
Additional Figures 16
Additional Tables 32
NMR Spectra 37
Mass Spectra 48
References 52

Experimental

General. Tetrahydrofuran was dried using a commercial solvent purification system. All other solvents and reagents were used as received. 1H NMR spectra were recorded on high-field spectrometers (^{1}H frequency 500.13 or 600.13 MHz), equipped with broadband inverse or conventional gradient probe heads. Spectra were referenced to the residual solvent signals (chloroform-*d*, 7.24 ppm). ^{13}C NMR spectra were recorded with ^{1}H broadband decoupling and referenced to solvent signals ($^{13}CDCl_3$, 77.0 ppm). Two-dimensional NMR spectra were recorded with 2048 data points in the t_2 domain and upto 2048 in the t_1 domain with a 1.5 s recovery delay. All 2D spectra were recorded with gradient selection except for ROESY. The ROESY spinlock time was 200ms. High resolution mass spectra were recorded using MALDI and ESI ionization in the positive mode on Bruker Apex ultra-FT-ICR. UV-vis-NIR absorption spectrometry was performed using the Perkin Elmer LAMBDA 1050 UV-vis-NIR spectrometer.

Oxidation of compound 1 with BAHA. The 1 mm path length spectrophotometer cell was filled with a 0.2 mL solution of compound **1** (0.108 mM) in DCM. The BAHA solution (0.2 equiv) was added to the above compound **1,** and the cell was tightly closed, and the mixture was shaken well and UV-vis-NIR spectra was measured. The same procedure was repeated, and the progress of the reaction was monitored spectrophotometrically with the addition of BAHA solution up to 2.4 equivalents. To the oxidized solution of compound $\mathbf{1}^{2+}$, hydrazine hydrate (6.0 equiv) was added and the mixture was shaken and recorded the absorption spectrum.

Reduction of compound 1 with sodium naphthalenide. Dry and deoxygenated tetrahydrofuran was stored for 24 h in an inert atmosphere of glove box over fresh sodium pieces before use. All operations were performed in the glove box. Sodium naphthalenide (0.025 M in THF) was prepared by stirring of naphthalene dried under high vacuum (16.0 mg, 0.125 mmol) with excess sodium metal in tetrahydrofuran (5.0 mL) for 24 h. The solution was dark yellow. The 1.0 mm path length spectrophotometer cell was filled with 0.2 mL solution of compound **1** (0.154 mM) and 15 crown 5 ether (100.0 equiv) in tetrahydrofuran. The sodium naphthalenide solution (0.5 equiv) was added to the above mixture inside the glove box, and the cell was tightly closed, and the mixture was shaken. The cell was removed from the glove box and UV-vis-NIR spectra were measured. The cell was taken back into the glove box and titration with sodium naphthalenide was continued, and the progress of the reaction was monitored spectrophotometrically with the addition of sodium naphthalenide up to 42.0 equivalents. The reduction–reoxidation sequence was performed entirely in the glove box. To the reduced solution of compound **1**, diiodine solution [(0.1 M in THF) was added up to 50.0 equivalents and the mixture was shaken. The cell was closed tightly and removed from the glove box, to record the absorption spectrum.

Reduction of compound 2 with cobaltocene (UV-VIS-NIR experiment). Dry and deoxygenated tetrahydrofuran was stored for 24 h in an inert atmosphere of glove box. All operations were performed in the glove box. Cobaltocene solution (1.0×10^{-3} M in THF) was prepared by dissolving cobaltocene (1.9 mg, 0.01 mmol) in 10 mL of THF. The 1 mm path length spectrophotometer cell was filled with a 0.2 mL solution of compound **2** (0.10 mM) in tetrahydrofuran. The cobaltocene solution (0.25 equiv) was added to the above compound **2** inside the glove box, and the cell was tightly closed, and the mixture was shaken. The cell was removed from the glove box and UV-vis-NIR spectra was measured. The same procedure was repeated, and the progress of the reaction was monitored spectrophotometrically with the addition of cobaltocene solution up to 2.5 equivalents. To the reduced solution of compound **2**, diiodine solution (0.1 M in THF) was added up to 2.5 equivalents and the

mixture was shaken. The cell was closed tightly and removed from the glove box, to record the absorption spectrum.

Reduction of compound 2 with cobaltocene (IR experiment). Dry and deoxygenated tetrahydrofuran was stored for 24 h in an inert atmosphere of the glove box. All operations were performed in the glove box. In a flame dried round bottom flask (5.0 mL), compound **2** (2.0 mg) was dissolved in dry THF (1.0 mL) and treated with 1.0 equiv of cobaltocene (0.094 mL, 1.06 x 10^{-2} M in THF). The colour of the reaction mixture was changed from red violet to violet for monoanionic species. The solution was shaken well and dry under vacuum in glove box. The obtained dark violet solid was mixed with dry KBr (300 mg) in the china dish and the resulted mixture was placed in the KBr pellet assembly. The assembly was taken out from the glove box and pressed under high pressure to prepare the KBr pellet and immediately recorded the IR spectrum. Similarly, we prepared the KBr pallet for the dianion species of compound **2** with the addition of 2.0 equiv of cobaltocene (0.188 mL, 1.06 x 10^{-2} M in THF). The colour changed for this reaction was from red violet to light green.

Reduction of compound 2 with sodium naphthalenide. Dry and deoxygenated tetrahydrofuran was stored for 24 h in an inert atmosphere of glove box over fresh sodium pieces before use. All operations were performed in the glove box. Sodium naphthalenide (0.025 M in THF) was prepared by stirring of naphthalene dried under high vacuum (16.0 mg, 0.125 mmol) with excess sodium metal in tetrahydrofuran (5.0 mL) for 24 h. The solution was dark yellow. The 1.0 mm path length spectrophotometer cell was filled with 0.2 mL solution of compound **2** (0.10 mM) and 15 crown 5 ether (100.0 equiv) in tetrahydrofuran. The sodium naphthalenide solution (0.5 equiv) was added to the above mixture inside the glove box, and the cell was tightly closed, and the mixture was shaken. The cell was removed from the glove box and UV-vis-NIR spectra was measured. The cell was taken back into the glove box and titration with sodium naphthalenide was continued, and the progress of the reaction was monitored spectrophotometrically with the addition of sodium naphthalenide up to 34.0 equivalents. The reduction–reoxidation sequence was performed entirely in the glove box. To the reduced solution of compound **2**, diiodine solution (0.1 M in THF) was added up to 34.0 equivalents and the mixture was shaken. The cell was closed tightly and removed from the glove box, to record the absorption spectrum.

Oxidation of compound 2 with BAHA. The 1 mm path length spectrophotometer cell was filled with a 0.2 mL solution of compound **2** (0.10 mM) in DCM. The BAHA solution (0.2 equiv) was added to the above compound **2,** and the cell was tightly closed, and the mixture was shaken well and UV-vis-NIR spectra was measured. The same procedure was repeated, and the progress of the reaction was monitored spectrophotometrically with the addition of BAHA solution up to 4.0 equivalents. To the oxidized solution of compound $\mathbf{2}^{+}$, hydrazinehydrate (6.0 equiv) was added and the mixture was shaken and recorded the absorption spectrum.

Computational methods. Density functional theory (DFT) calculations were performed using Gaussian 16.[1] DFT geometry optimizations were carried out in unconstrained C_1 symmetry, using molecular mechanics or semiempirical models as starting geometries. The calculations were performed using the hybrid functional B3LYP,[2–4] including the CAM[5] and GD3BJ[6] corrections, and the 6-31G(d,p) basis set. Wavefunctions were tested for instabilities and, if required, reoptimized until a stable solution was found. Each structure was optimized to meet standard convergence criteria. The existence of a local minimum was verified by a normal mode frequency calculation. Nucleus-Independent Chemical Shift

(NICS)[7] maps were obtained at the CAM level of theory, by evaluating GIAO shieldings over a square grid of 201 × 201 points and located 1 Å above the plane of the molecule. The plots correspond to the anisotropic shielding value in the direction perpendicular to the cross-section plane.

X-ray crystallography. X-Ray quality crystal of compound **1** was grown from the concentrated solution of *n*-hexane and for compound **2** was grown by vapor diffusion of *n*-hexane into concentrated solution of **2** in toluene. Diffraction data were collected on a Rigaku Oxford Diffraction XtaLAB Synergy-R DW diffractometer equipped with a HyPix ARC 150° Hybrid Photon Counting (HPC) detector using CuKα (λ = 1.5418 Å) for compound **2** at 100 K. Data collection, cell refinement, data reduction and analysis were carried out with the Xcalibur PX software, CRYSALIS CCD and CRYSALIS RED, respectively (Oxford Diffraction Ltd., Abignon, England, 2009). An analytical absorption correction was applied with the use of CRYSALIS RED. All structures were solved by direct methods with the SHELXS-97 program and refined using SHELXL-97 with anisotropic thermal parameters for non-H atoms. In the final refinement cycles, all H atoms were treated as riding atoms in geometrically optimized positions. CCDC 2381163, 2381426, and 2381427 contain the supplementary crystallographic data for this paper. These data are provided free of charge by The Cambridge Crystallographic Data Centre via www.ccdc.cam.ac.uk/data_request/cif.

Synthesis

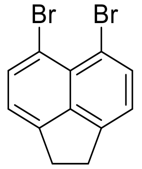

5,6-Dibromo-1,2-dihydroacenaphthylene (S2). Compound **S2** was prepared according to a literature procedure.[8] Yield 25%. **^{1}H NMR** (500 MHz, chloroform-*d* , 300 K): δ 7.78 (d, *J* = 7.5 Hz, 2H), 7.08 (d, *J* = 7.5 Hz, 2H), 3.29 (s, 4H).

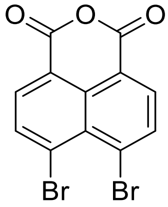

6,7-Dibromo-1*H*,3*H*-benzo[*de*]isochromene-1,3-dione (S3). Compound **S3** was prepared according to a literature procedure.[9] Since the anhydride was insoluble, it was used in subsequent steps without purification

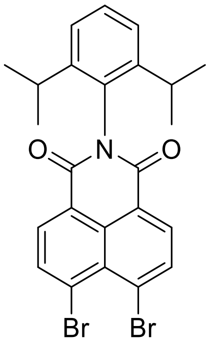

6,7-Dibromo-2-(2,6-diisopropylphenyl)-1*H*-benzo[*de*]isoquinoline-1,3(2*H*)-dione (S4). Compound **S4** was prepared according to a literature procedure.[9] Yield 40%. **^{1}H NMR** (500 MHz, chloroform-*d* , 300 K): δ 8.45 (d, *J* = 8.0 Hz, 2H), 8.25 (d, *J* = 8.0 Hz, 2H), 7.46 (t, *J* = 7.5 Hz, 1H), 7.30 (d, *J* = 7.5 Hz, 2H), 2.64 (m, 2H), 1.12 (d, *J* = 7.0 Hz, 12H).

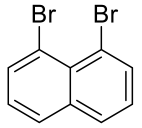

1,8-Dibromonaphthalene (S6). Compound **S6** was prepared according to a literature procedure.[10] Yield 22%. **^{1}H NMR** (500 MHz, chloroform-*d* , 300 K): δ 7.93 (dd, *J* = 7.5, 1.0 Hz, 2H), 7.81 (dd, *J* = 8.0, 1.0 Hz, 2H), 7.25 (m, 2H).

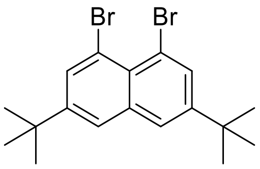

1,8-Dibromo-3,6-di-*tert*-butylnaphthalene (6). A flame-dried 25 mL round-bottom flask was equipped with a spin vane magnetic stir-bar, 1,8-dibromonaphthalene (400 mg, 1.55 mmol, 1.0 equiv) was dissolved in tert-butyl chloride (2.02 mL, 18.6 mmol, 12.0 equiv) at 45 °C, aluminium chloride (41.4 mg, 0.031 mmol, 0.2 equiv) was added during 1 hour and the reaction mixture was stirred at 45 °C for 24 h. The reaction mixture was quenched with water and extract with ethyl acetate, then dried over sodium sulfate and evaporated under reduced pressure. The residue was purified by column chromatography *via* silica gel using *n*-hexane as an eluent to provide compound **6** (480 mg, 78% yield) as white solid. **^{1}H NMR** (500 MHz, chloroform-*d* , 300 K): δ 7.90 (d, *J* = 2.0 Hz, 2H), 7.67 (d, *J* = 2.0 Hz, 2H), 1.36 (s, 18H). **^{13}C NMR** (151 MHz, chloroform-*d* , 300 K): δ 149.39, 137.09, 133.60, 125.50, 124.91, 119.17, 34.50, 30.92. **HRMS** (ESI–TOF): *m/z*: $[M + K]^+$ Calcd for $C_{18}H_{22}Br_2K$: 436.9700; Found 437.1949.

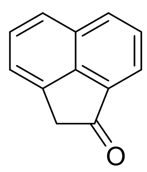

Acenaphthylen-1(2*H*)-one (S7). Compound **S7** was prepared by the following literature procedure.[11] Yield 55%. **^{1}H NMR** (500 MHz, chloroform-*d* , 300 K): δ 8.07 (d, *J* = 8.5 Hz, 1H), 7.94 (d, *J* = 7.0 Hz, 1H), 7.80 (d, *J* = 8.5 Hz, 1H), 7.69 (m, 1H), 7.58 (dd, *J* = 8.5, 7.0 Hz, 1H), 7.44 (d, *J* = 6.5 Hz, 1H), 3.80 (d, *J* = 1.0 Hz, 2H).

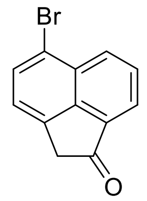

5-Bromoacenaphthylen-1(2*H*)-one (3). A flame-dried 100 mL round-bottom flask was equipped with a spin vane magnetic stir-bar, acenaphthylen-1(2H)-one (2.5 g, 14.9 mmol, 1 equiv) was dissolved in *N,N*-dimethylformamide (25 ml), *N*-bromosuccinimide (2.65 g, 14.9 mmol, 1 equiv) was added and the reaction mixture was stirred at room temperature for 2 days. The precipitated crystals were collected by filtration and recrystallized from ethanol to give 5-bromoacenaphthen-1-one (1.9 g, 52% yield) as white solid. **^{1}H NMR** (500 MHz, chloroform-*d* , 300 K): δ 8.25 (d, *J* = 8.0 Hz, 1H), 7.97 (d, *J* = 7.5 Hz, 1H), 7.79 (m, 2H), 7.30 (d, *J* = 7.5 Hz, 1H), 3.75 (d, *J* = 1.0 Hz, 2H). **^{13}C NMR** (125 MHz, chloroform-*d* , 300 K): δ 201.79, 143.56, 134.94, 134.68, 131.46, 130.88, 130.55, 129.15, 122.28, 121.98, 118.77, 41.56. **HRMS** (ESI–TOF): *m/z*: $[M + Na]^+$ Calcd for $C_{12}H_7BrONa$: 268.9572; Found 268.9569.

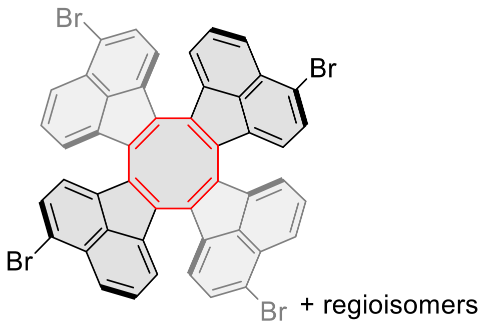

1,7,13,19-Tetrabromocycloocta[1,2-*a*:3,4-*a*':5,6-*a*'':7,8-*a*''']tetraacenaphthylene (4). A flame-dried 2-necked 100 mL round-bottom flask was equipped with a spin vane magnetic stir-bar, a reflux condenser and purged with an atmosphere of nitrogen. Titanium tetrachloride (0.94 mL, 8.6 mmol) and *o*-dichlorobenzene (10.5 mL) were added to the flask and the reaction mixture was brought to reflux under nitrogen. 5-bromoacenaphthylen-1(2H)-one, (0.354 g, 1.43 mmol) was dissolved in *o*-dichlorobenzene (10.5 mL) and slowly added to the refluxing mixture. The reaction mixture was refluxed for 3 h. The reaction mixture was then poured (while hot) into a 250 mL flask containing crushed ice and concentrated HCl (aq.) (10 mL). After the ice melted, the reaction mixture was extracted with chloroform. Organic layer was dried over anhydrous Na_2SO_4 and evaporated under reduced pressure. The residue was purified by column chromatography *via* silica gel using dichloromethane: *n*-hexane (20:80, v/v) as an eluent to provide compound **4** (160 mg, 49% yield) as brown solid. **^{1}H NMR** (500 MHz, chloroform-*d* , 300 K): δ 8.02 (m, 4H), 7.74 (m, 4H), 7.60 (m, 8H), 7.34 (m, 4H). **HRMS** (MALDI–TOF): *m/z*: $[M]^+$ Calcd for $C_{48}H_{20}Br_4$: 915.8260; Found 915.8234.

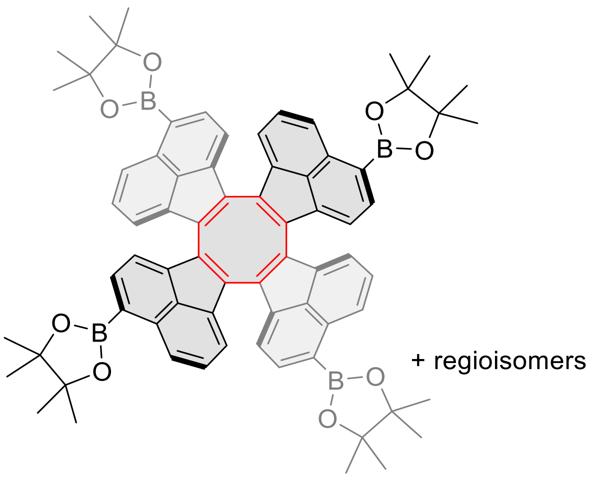

1,7,13,19-Tetrakis(4,4,5,5-tetramethyl-1,3,2-dioxaborolan-2-yl)cycloocta[1,2-*a*:3,4-*a*':5,6-*a*'':7,8-*a*''']tetraacenaphthylene (5). Compound **3** (100.0 mg, 0.11 mmol, 1 equiv), bis(pinacolato)diboron (166.3 mg, 0.66 mmol, 6 equiv), Pd(dppf)Cl_2 (12.0 mg, 0.0164 mmol, 0.15 equiv), and potassium acetate (128.5 mg, 1.31 mmol, 12 equiv) were dissolved in degassed 1,4-dioxane (4.0 mL) under argon atmosphere of glovebox. The mixture was heated at 110°C for overnight. The reaction mixture was cooled to room temperature, and then added to water before being extracted with dichloromethane. Combined organic layers were washed with water and brine, then dried over sodium sulfate and evaporated under reduced pressure. The crude washed with methanol and used for next reaction without furthere purification (110 mg, 91%). **^{1}H NMR** (500 MHz, chloroform-*d* , 300 K): δ 8.59 (m, 4H),

8.12 (m, 4H), 7.58 (m, 12H), 1.41 (s, 48H). **HRMS** (MALDI–TOF): *m/z*: [M]$^+$ Calcd for $C_{72}H_{68}B_4O_8$: 1104.5319; Found 1104.5318.

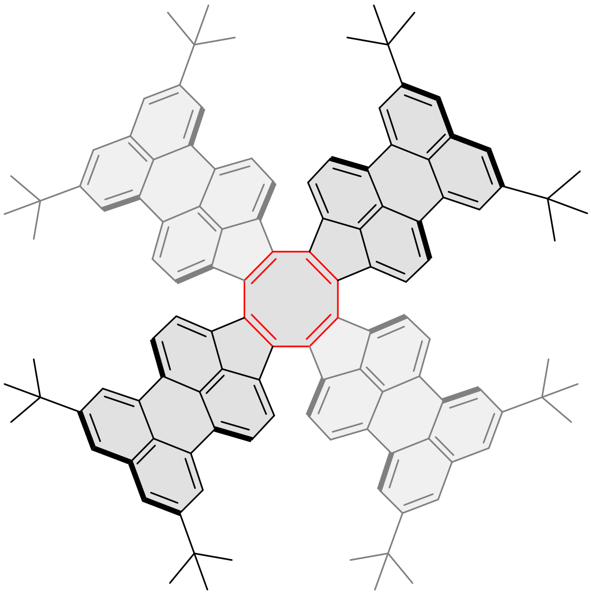

Pentacosacyclene (1). A Schlenk tube was charged with compound **5** (25 mg, 0.0226 mmol, 1.0 equiv), 1,8-dibromo-3,6-di-tert-butylnaphthalene **6** (54.0 mg, 0.136 mmol, 6.0 equiv), tris-(dibenzylideneacetone)dipalladium(0)–chloroform adduct (9.37 mg, 0.00905 mmol, 0.40 equiv), Tri-m-tolylphosphine (11.0 mg, 0.0362 mmol, 1.60 equiv), Cs_2CO_3 (88.5 mg, 0.272 mmol, 12.0 equiv) and 1-chloronaphthalene (2.0 mL) in an inert atmosphere of glovebox. The reaction mixture was stirred at 160 °C for 24 h. After cooling down to room temperature, *n*-hexane (10 mL) was added to the reaction mixture and passed through a column (silica gel), the 1-chloronaphthalene was removed *via* using dichloromethane: *n*-hexane (5:95 v/v) and the desired product **1** (6.3 mg, 18%) as red violet solid was obtained *via* using dichloromethane: *n*-hexane (8:92, v/v) as an eluting solvents. ^{1}**H NMR** (500 MHz, chloroform-*d* , 300 K): δ 8.55 (d, *J* = 1.5 Hz, 8H), 8.39 (d, *J* = 7.5 Hz, 8H), 7.93 (d, *J* = 8.0 Hz, 8H), 7.82 (d, *J* = 1.5 Hz, 8H), 1.52 (s, 72H). ^{13}**C NMR** (151 MHz, chloroform-*d* , 300 K): δ 149.12, 139.06, 136.99, 134.47, 132.23, 130.69, 129.27, 126.42, 125.53, 124.13, 123.61, 120.60, 119.83, 35.01, 31.36. **HRMS** (MALDI–TOF): *m/z*: [M]$^+$ Calcd for $C_{120}H_{104}$: 1545.8162; Found 1545.8162. **UV-vis** (DCM, 300 K) λ [nm] (ε in M^{-1} cm^{-1}): 485, 532, 564.

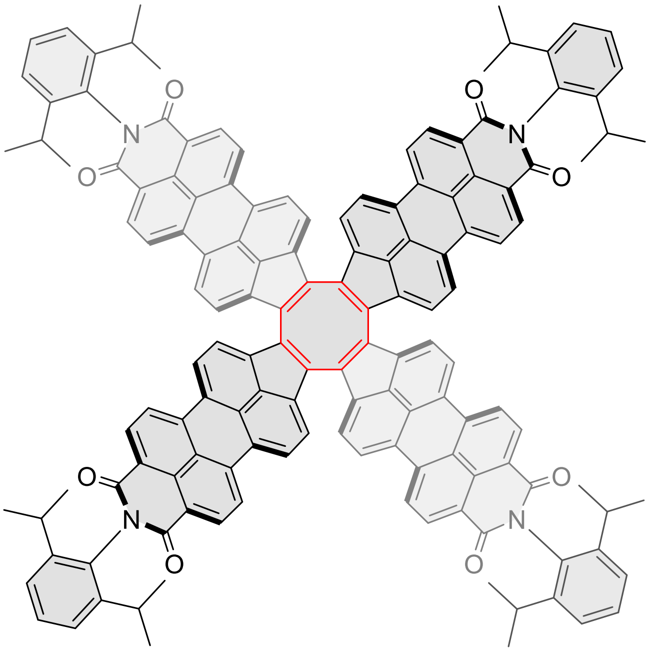

Pentacosacyclene tetraimide (2). A Schlenk tube was charged with compound **5** (15.0 mg, 0.0115 mmol, 1.0 equiv), 6,7-dibromo-2-(2,6-diisopropylphenyl)-1H-benzo[*de*]isoquinoline-1,3(2*H*)-dione **S4** (36.0 mg, 0.0693 mmol, 6.0 equiv), tris-(dibenzylideneacetone)dipalladium(0)–chloroform adduct (4.8 mg, 0.00462 mmol, 0.40 equiv), tricyclohexylphosphine tetrafluoroborate (6.8 mg, 0.0185 mmol, 1.60 equiv), Cs_2CO_3 (45.0 mg, 0.139 mmol, 12.0 equiv) and 1-chloronaphthalene (3.0 mL) in an inert atmosphere of glovebox. The reaction mixture was stirred at 160 °C for 24 h. After cooling down to room temperature, *n*-hexane (10 mL) was added to the reaction mixture and passed through a column (silica gel), the 1-chloronaphthalene was removed *via* using dichloromethane: *n*-hexane (30:70 v/v) and the desired product **2** (11 mg, 47%) as red violet solid was obtained *via* using dichloromethane: *n*-hexane (75:25, v/v) as an eluting solvents. **^{1}H NMR** (500 MHz, chloroform-*d* , 300 K): δ 8.72 (d, *J* = 8.0 Hz, 8H), 8.61 (d, *J* = 8.0 Hz, 8H), 8.47 (d, *J* = 7.5 Hz, 8H), 7.90 (d, *J* = 7.5 Hz, 8H), 7.47 (t, *J* = 8.0 Hz, 4H), 7.34 (d, *J* = 8.0 Hz, 4H), 7.31 (d, *J* = 8.0 Hz, 4H), 2.81 (p, *J* = 7.0 Hz, 4H), 2.73 (p, *J* = 7.0 Hz, 4H), 1.21 (d, *J* = 7.0 Hz, 24H), 1.13 (d, *J* = 7.0 Hz, 24H). **^{13}C NMR** (151 MHz, chloroform-*d* , 300 K): δ 163.64, 145.71, 145.56, 141.42, 138.14, 135.50, 131.75, 131.03, 130.67, 130.18, 129.62, 126.58, 125.69, 124.05, 123.69, 122.75, 122.35, 29.30, 29.20, 24.08, 23.92. **HRMS** (MALDI–TOF): *m/z*: $[M + H]^+$ Calcd for $C_{144}H_{101}N_4O_8$: 2014.7647; Found 2014.7649. **UV-vis** (THF, 300 K) λ [nm] (ε in M^{-1} cm^{-1}): 309, 351, 537, 573.

Additional Schemes

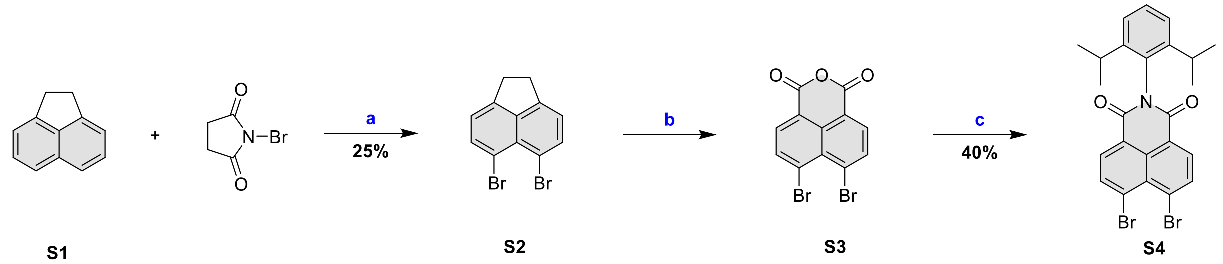

Scheme S1. Synthesis of compound **S4**. Reagents and conditions: (a) *N*-Bromosuccinimide, DMF: THF (1: 3), 10 °C, 18 h, warm to rt; (b) $K_2Cr_2O_7$, CrO_3, acetic acid, reflux, overnight; (c) 2,6-diisopropylaniline, *N*-methyl-2-pyrrolidone, propionic acid, 160 °C, overnight.

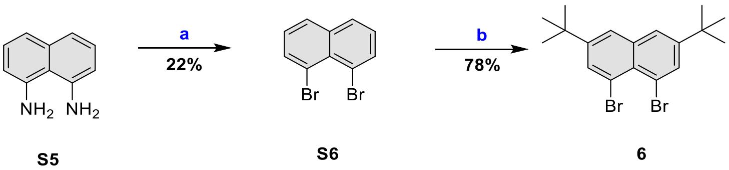

Scheme S2. Synthesis of compound **1**. Reagents and conditions: (a) H_2SO_4, $NaNO_2$, -15 °C, 15 minutes, 48% aq. HBr, CuBr, heat to 60 °C, 2 h; (b) tert-butyl chloride, $AlCl_3$, 45 °C, overnight.

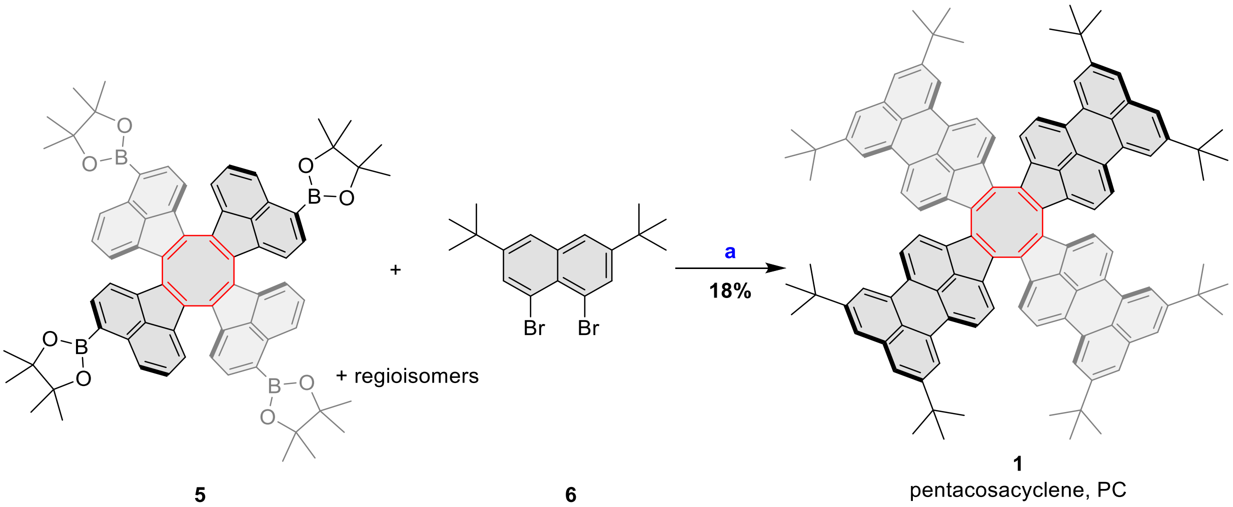

Scheme S3. Synthesis of compound **1**. Reagents and conditions: (a) $Pd_2(dba)_3.CHCl_3$, P(*m*-totyl)$_3$, Cs_2CO_3, 1-chloronaphthalene, 160 °C, 48 h.

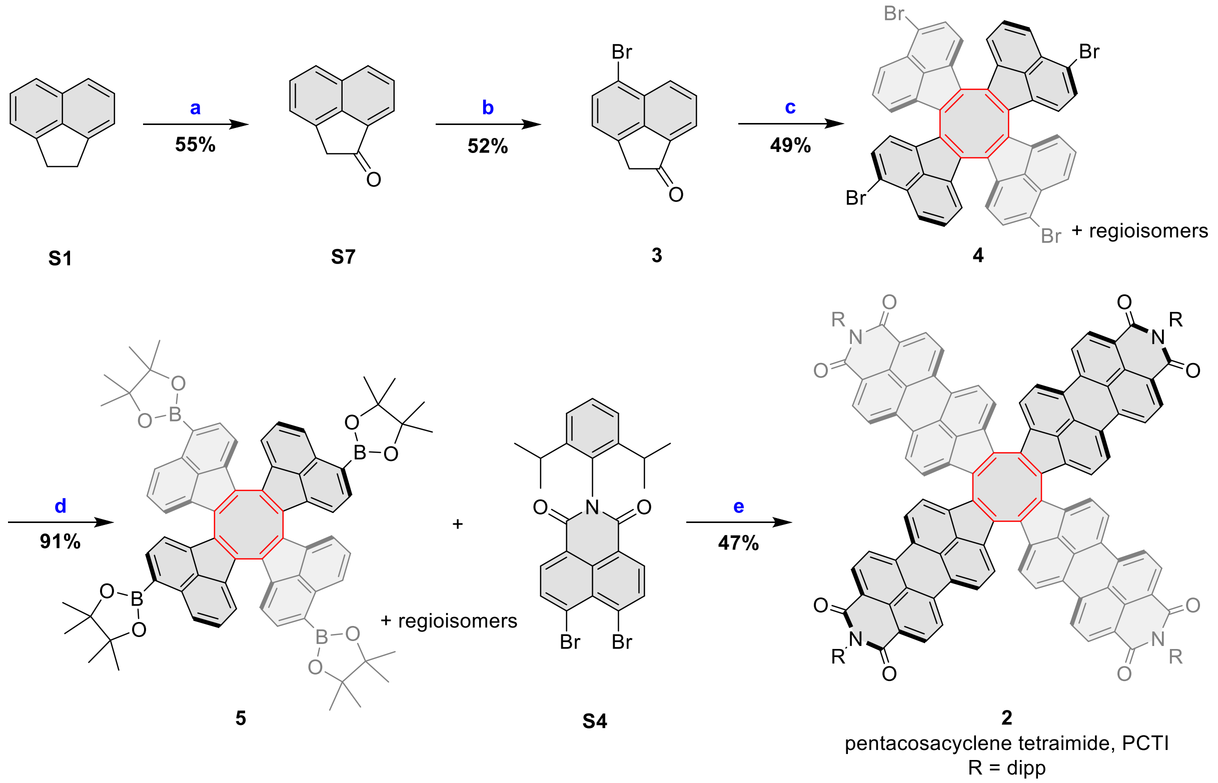

Scheme S4. Synthesis of compound **2**. Reagents and conditions: (a) $Na_2Cr_2O_7.2H_2O$, acetic acid, rt, 8 h; (b) *N*-Bromosuccinimide, DMF, rt, 2 days; (c) $TiCl_4$ (6.0 equiv), *o*-dichlorobenzene, reflux, 3 h; (d) Bis(pinacolato)diboron, Pd(dppf)Cl_2, KOAc, 1,4-dioxane, 110 °C, overnight; (e) $Pd_2(dba)_3.CHCl_3$, $PCy_3.HBF_4$, Cs_2CO_3, 1-chloronaphthalene, 160 °C, 24 h.

Additional Figures

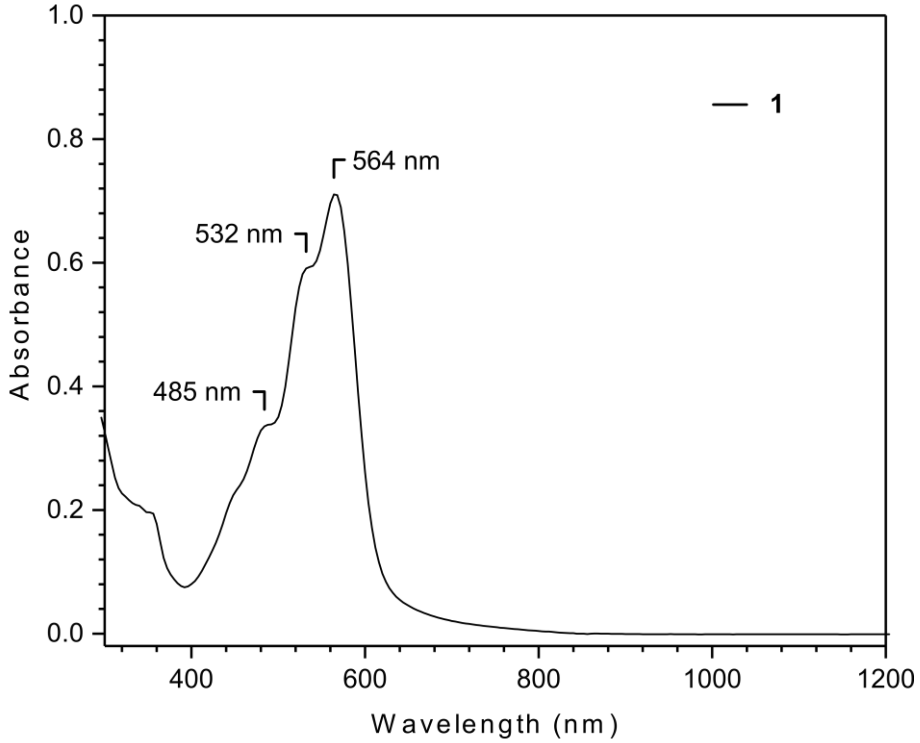

Figure S1. UV Spectrum of compound **1** (1.08 X 10^{-4} M) in 1 mm UV-cuvette.

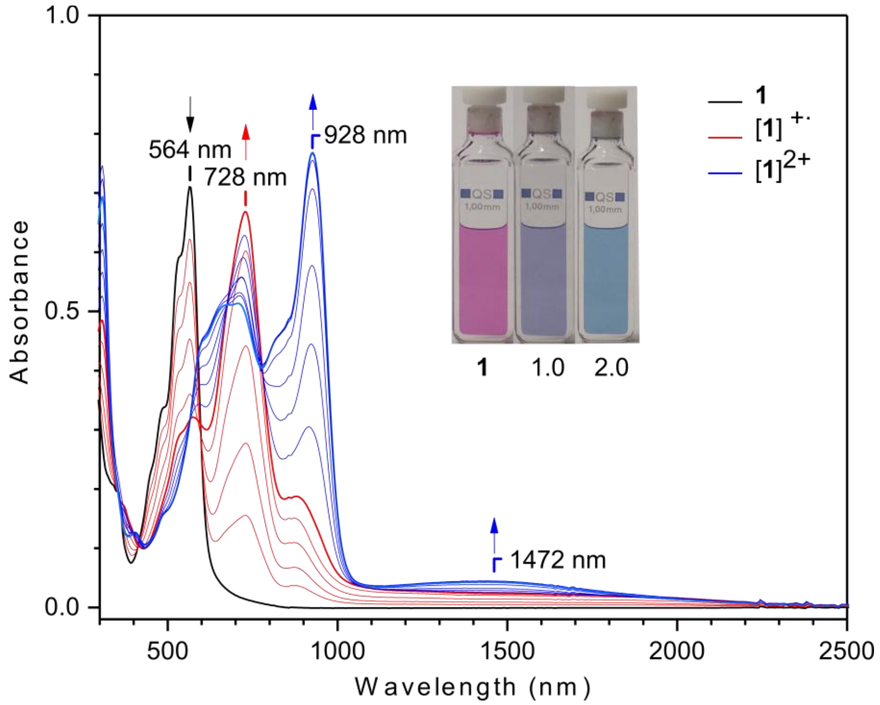

Figure S2. Oxidation of compound **1** with BAHA in DCM. Concentration of compound **1** (1.08 X 10^{-4} M) in 1 mm UV-cuvette.

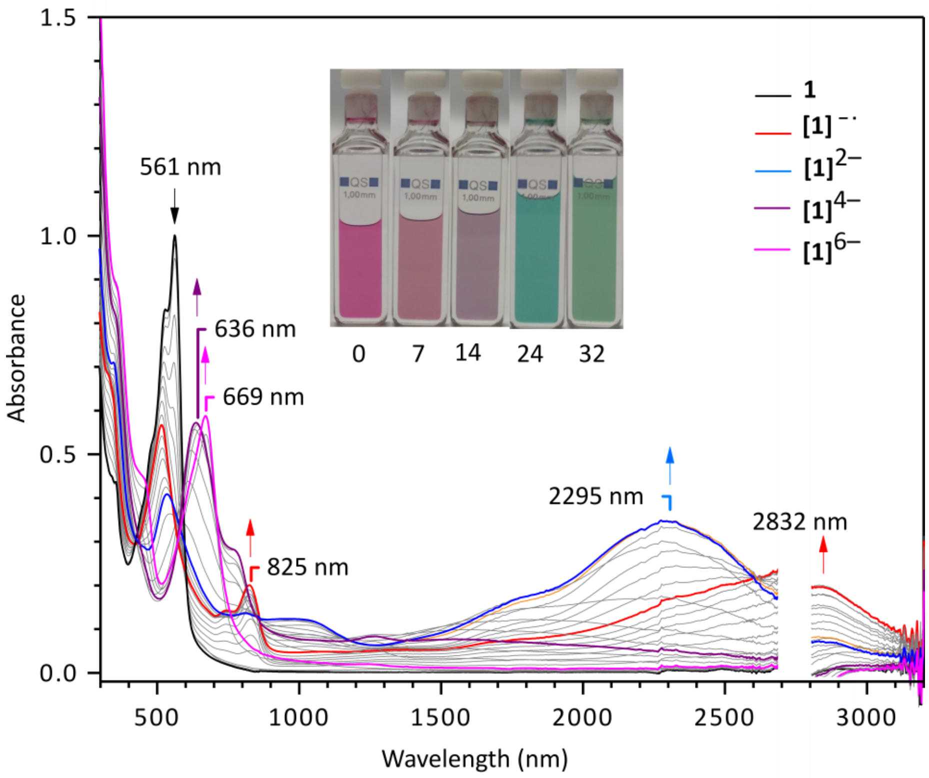

Figure S3. Reduction of compound **1** with NaN (sodium naphthanelide) in the presence of 15 crown 5 ether (100.0 equiv) in THF. Concentration of compound **1** (1.54 X 10-4 M) in 1 mm UV-cuvette.

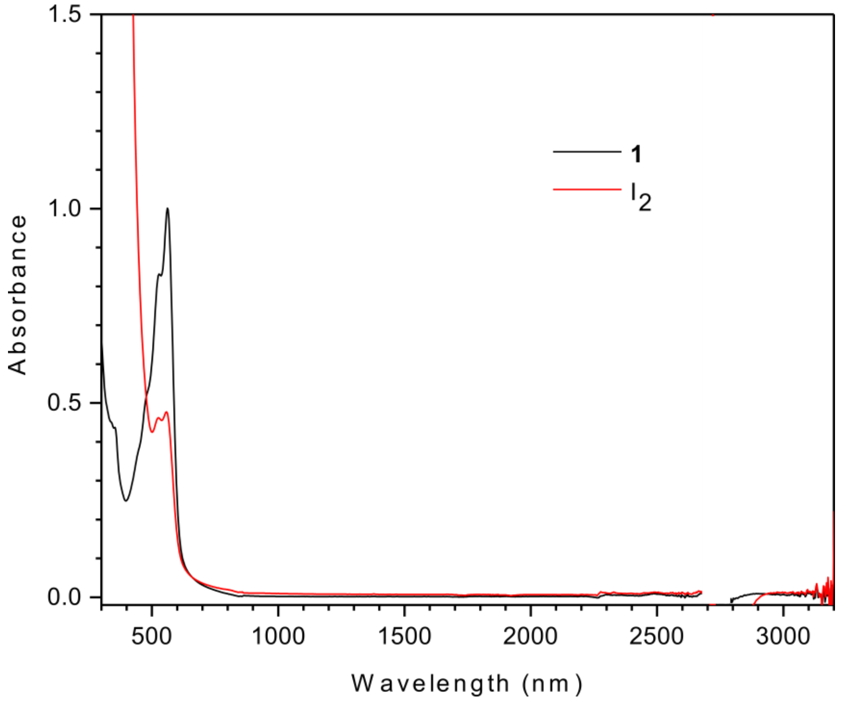

Figure S4. UV-vis-NIR Spectrum of compound **1** reduced with NaN in THF in the presence of 15 crown 5 ether (100 equiv) and oxidized back to neutral with iodine. Concentration of compound **2** (1.54 X 10^{-4} M) in 1 mm UV-cuvette.

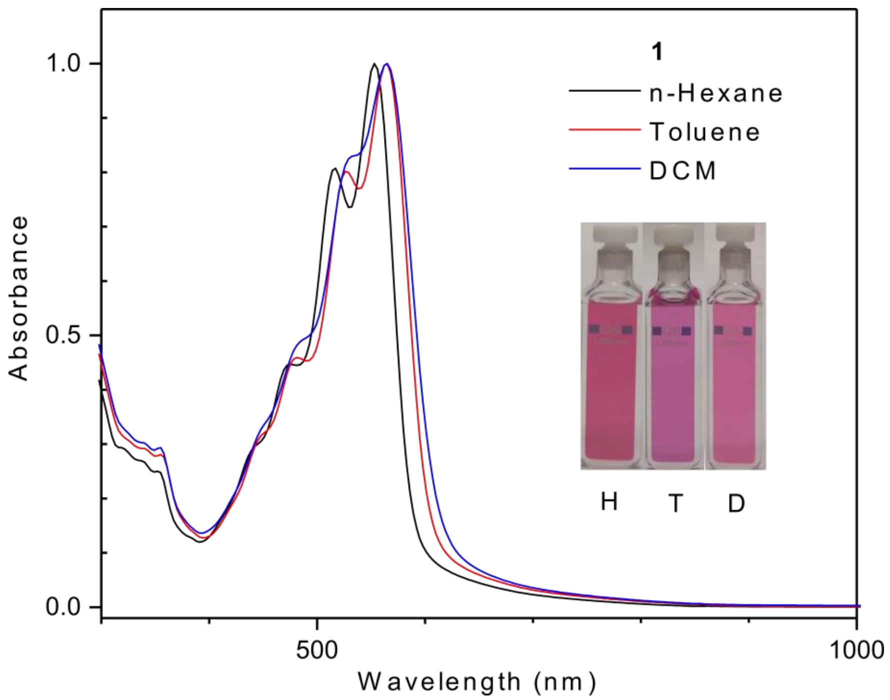

Figure S5. Solvent effect: UV-vis spectra of **1** in different solvents.

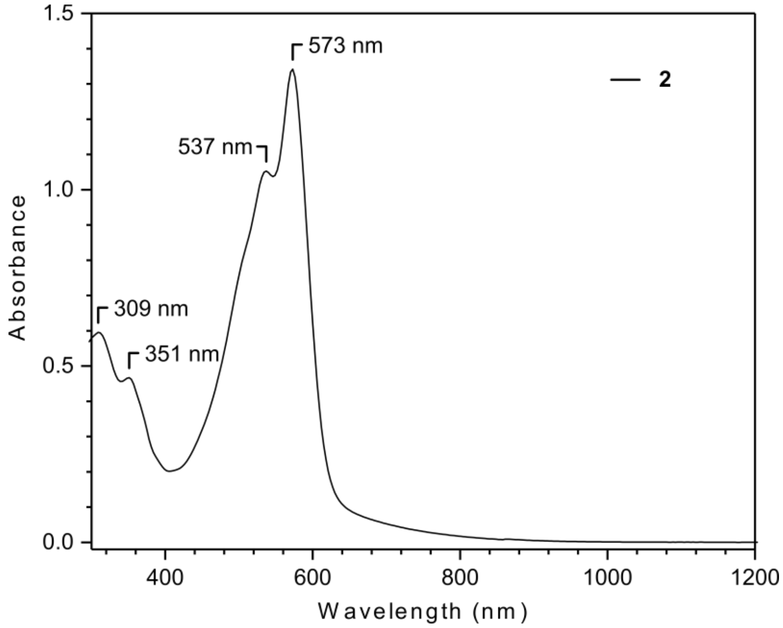

Figure S6. UV-vis Spectrum of compound **2** in THF. Concentration of compound **2** (1.0 X 10^{-4} M) in 1 mm UV-cuvette.

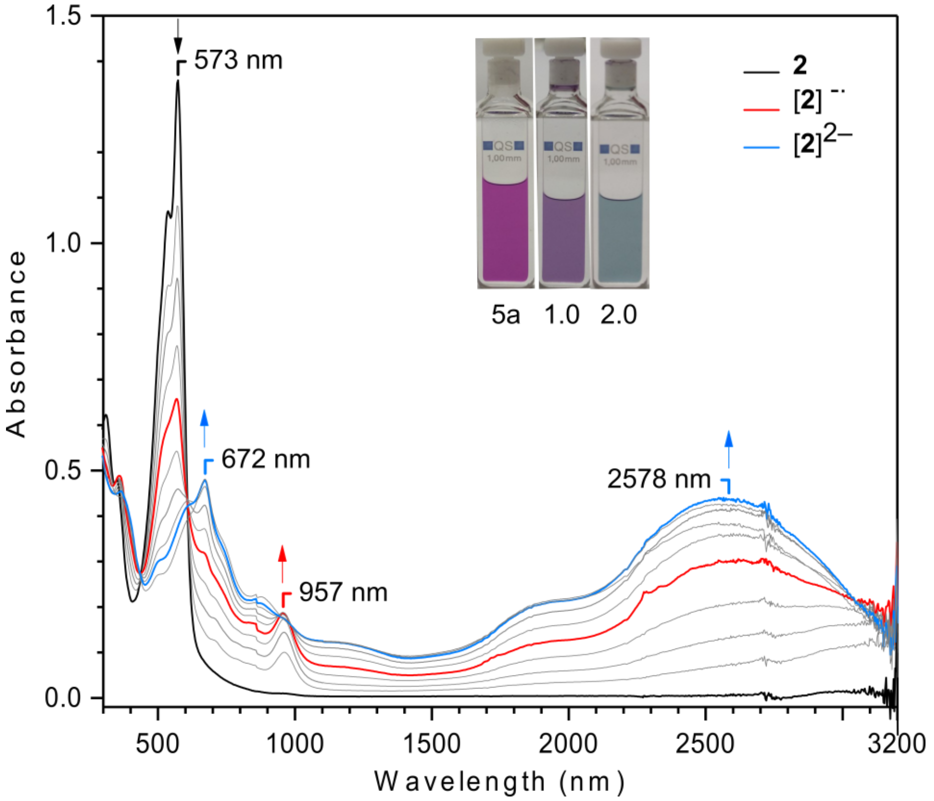

Figure S7. Reduction of compound **2** with cobaltocene in THF. Concentration of compound **2** (1.0 X 10^{-4} M) in 1 mm UV-cuvette.

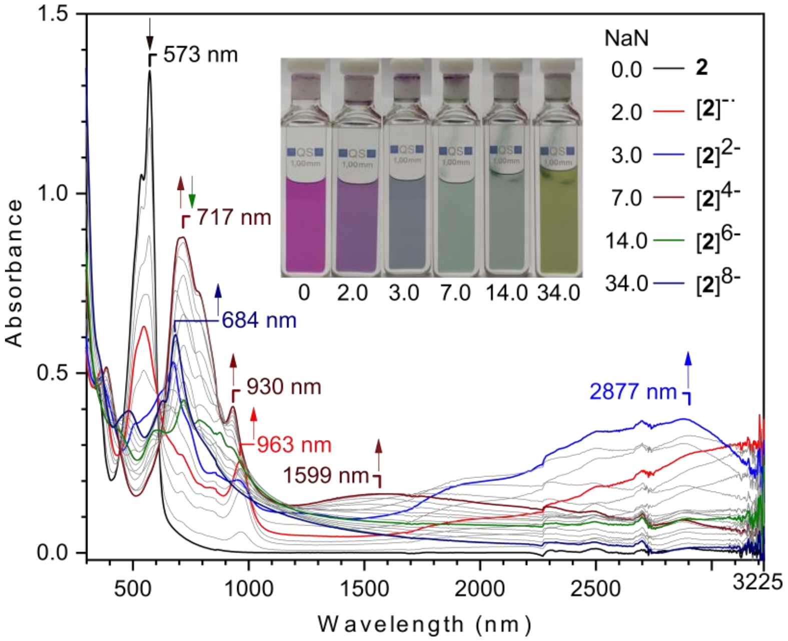

Figure S8. Reduction of compound **2** with NaN (sodium naphthanelide) in the presence of 15 crown 5 ether (100.0 equiv) in THF. Concentration of compound **2** (1.0 X 10^{-4} M) in 1 mm UV-cuvette.

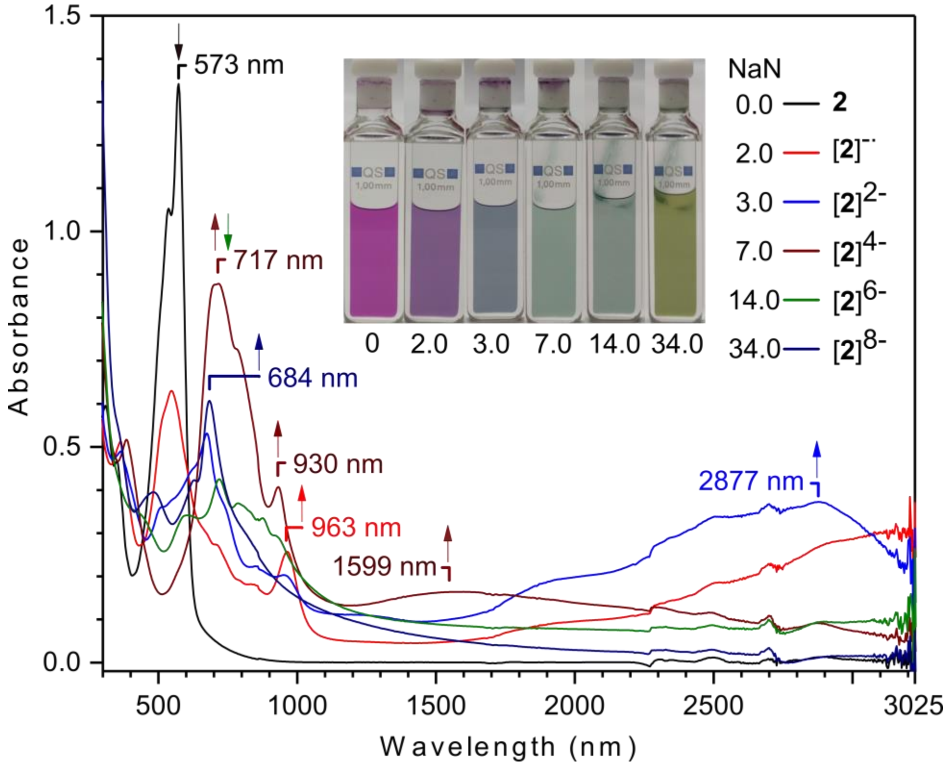

Figure S9. Reduction of compound **2** with NaN (sodium naphthanelide) in the presence of 15 crown 5 ether (100.0 equiv) in THF. Concentration of compound **2** (1.0×10^{-4} M) in 1 mm UV-cuvette.

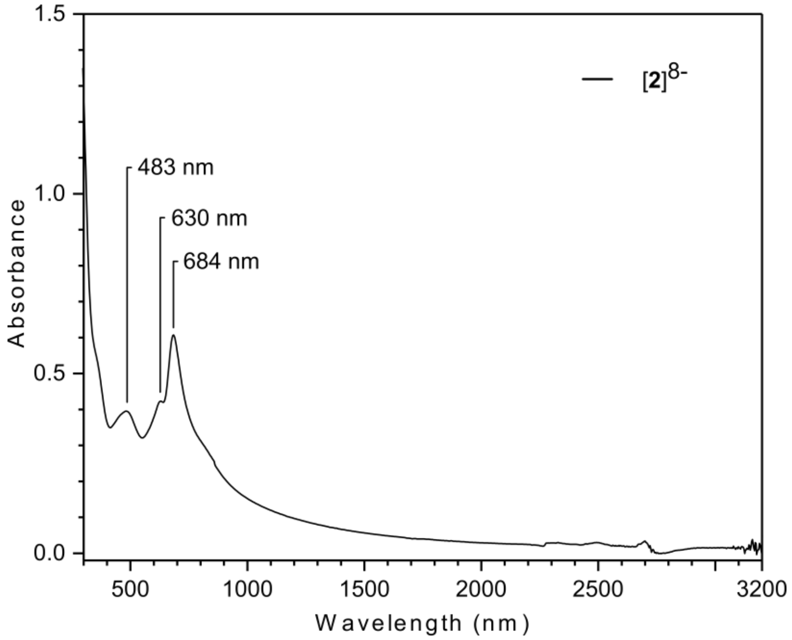

Figure S10. UV-vis-NIR Spectrum of compound [**2**]$^{8-}$ reduced with NaN in THF in the presence of 15 crown 5 ether (100 equiv). Concentration of compound **2** (1.0×10^{-4} M) in 1 mm UV-cuvette.

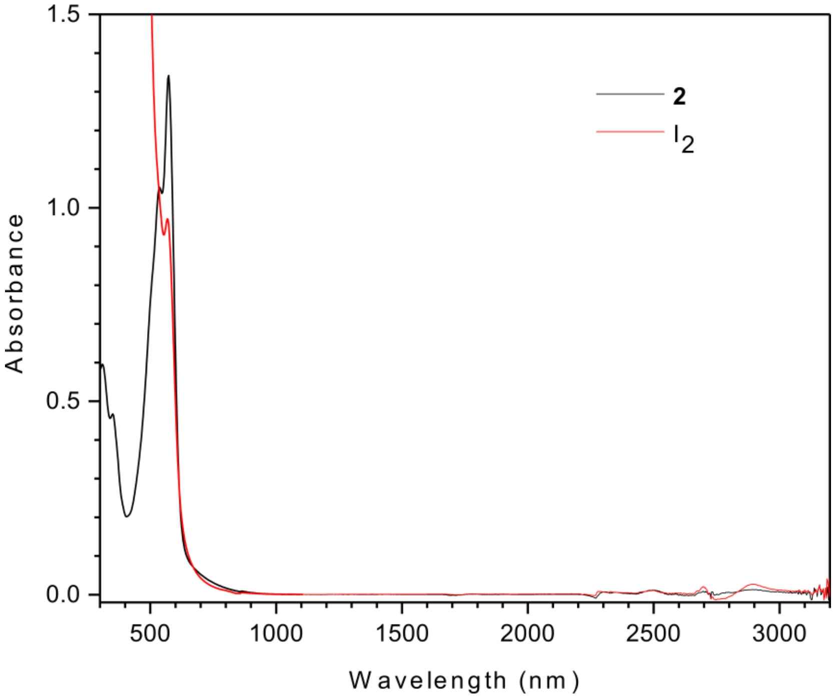

Figure S11. UV-vis-NIR Spectrum of compound **2** reduced with NaN in THF in the presence of 15 crown 5 ether (100 equiv) and oxidized back to neutral with iodine. Concentration of compound **2** (1.0 X 10^{-4} M) in 1 mm UV-cuvette.

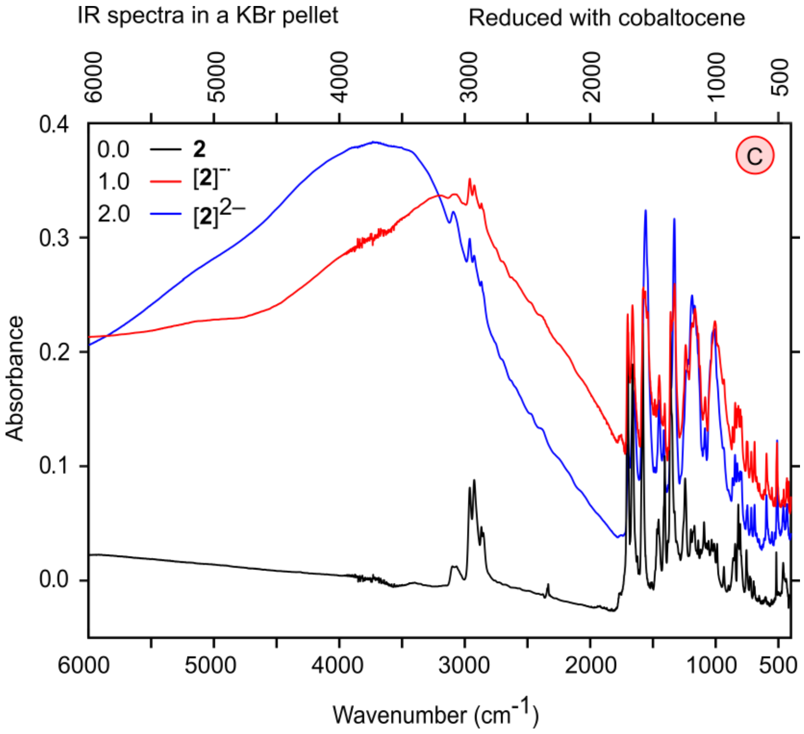

Figure S12. IR Spectra of compound **2** in a KBr pellet.

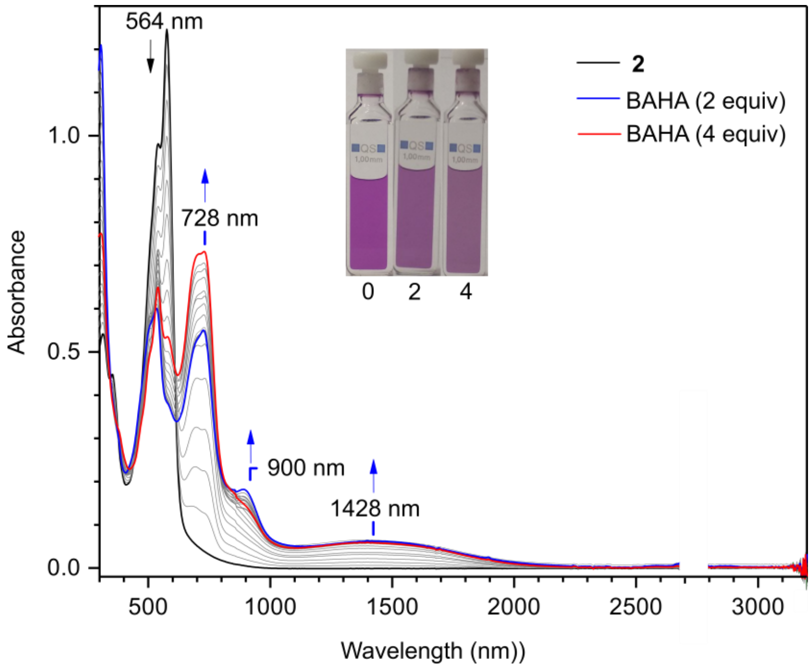

Figure S13. Oxidation of compound **2** with BAHA in DCM. Concentration of compound **2** (1.0×10^{-4} M) in 1 mm UV-cuvette.

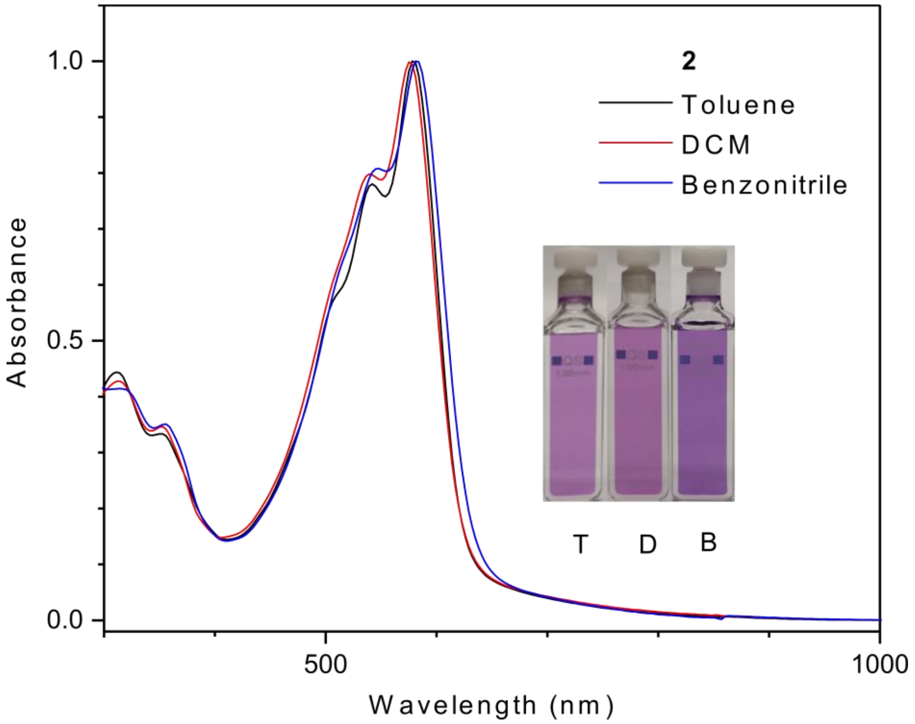

Figure S14. Solvent effect: UV-vis spectra of **2** in different solvents.

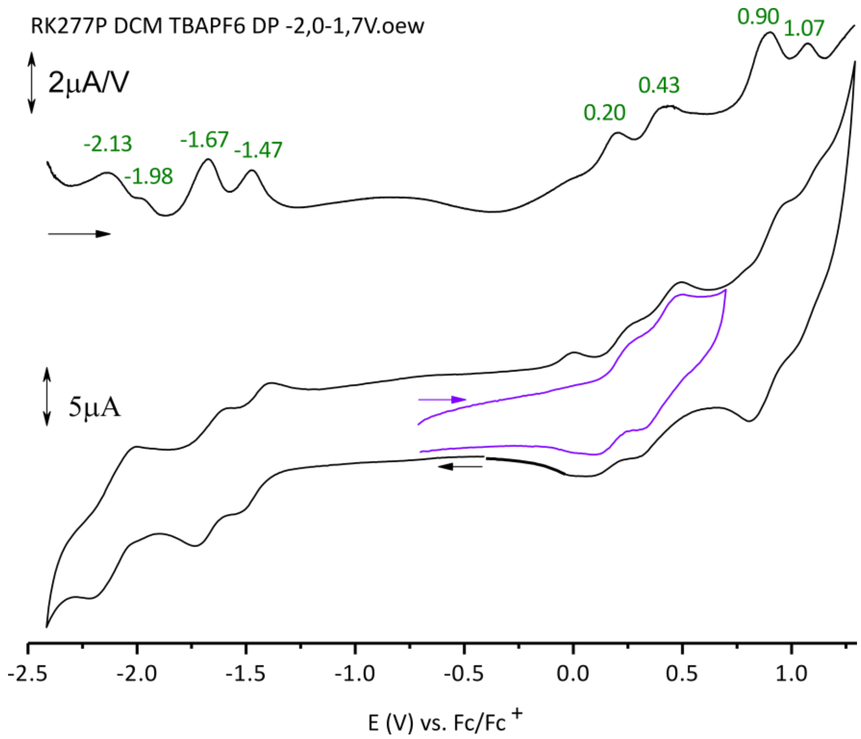

Figure S15. Cyclic voltammogram (bottom) and differential pulse voltammogram (top) recorded for compound **1** (DCM, $[Bu_4N]PF_6$, glassy-carbon electrode, 100 mV/s).

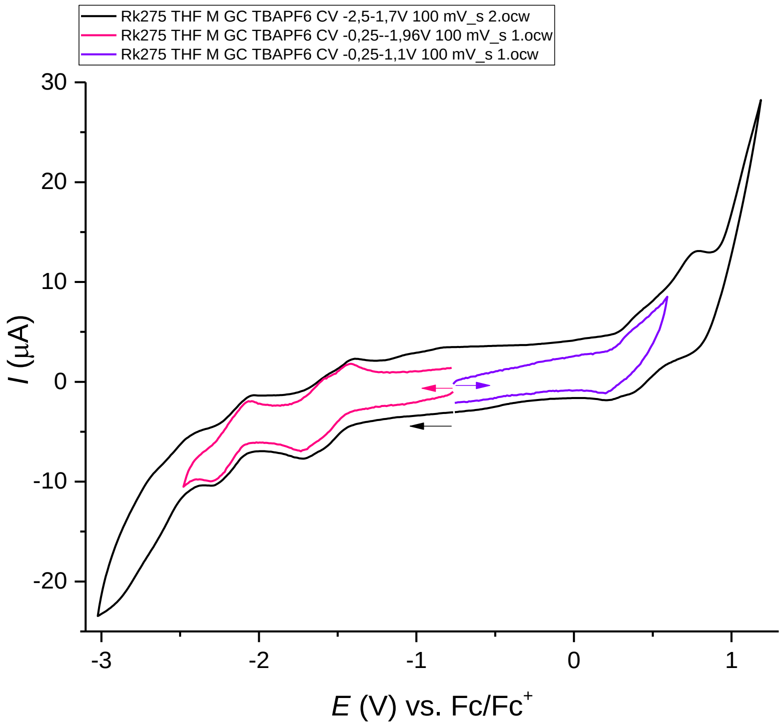

Figure S16. Cyclic voltammogram recorded for compound **1** (THF, $[Bu_4N]PF_6$, glassy-carbon electrode, 100 mV/s).

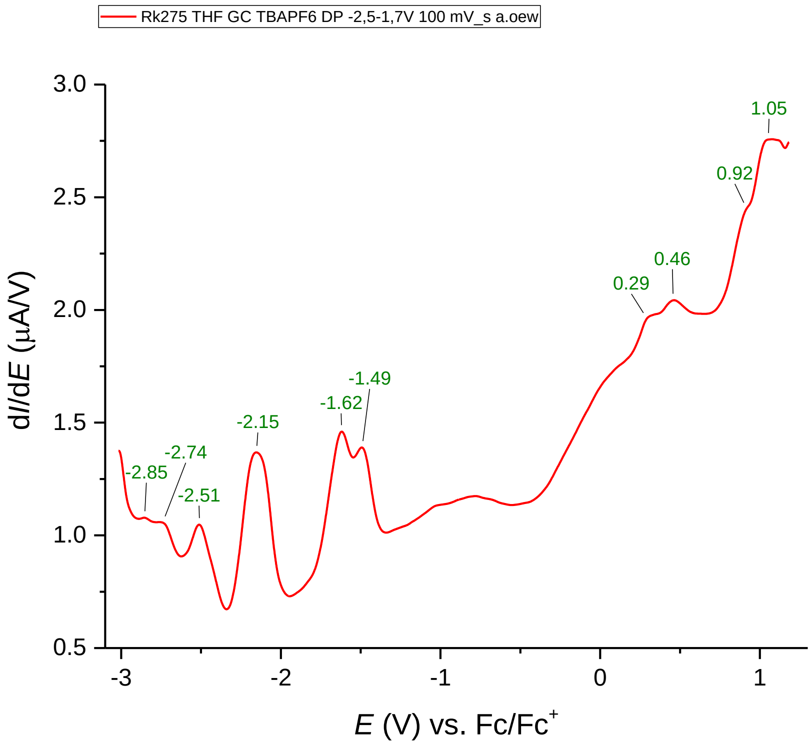

Figure S17. Differential pulse voltammogram recorded for compound **1** (THF, $[Bu_4N]PF_6$, glassy-carbon electrode, 100 mV/s).

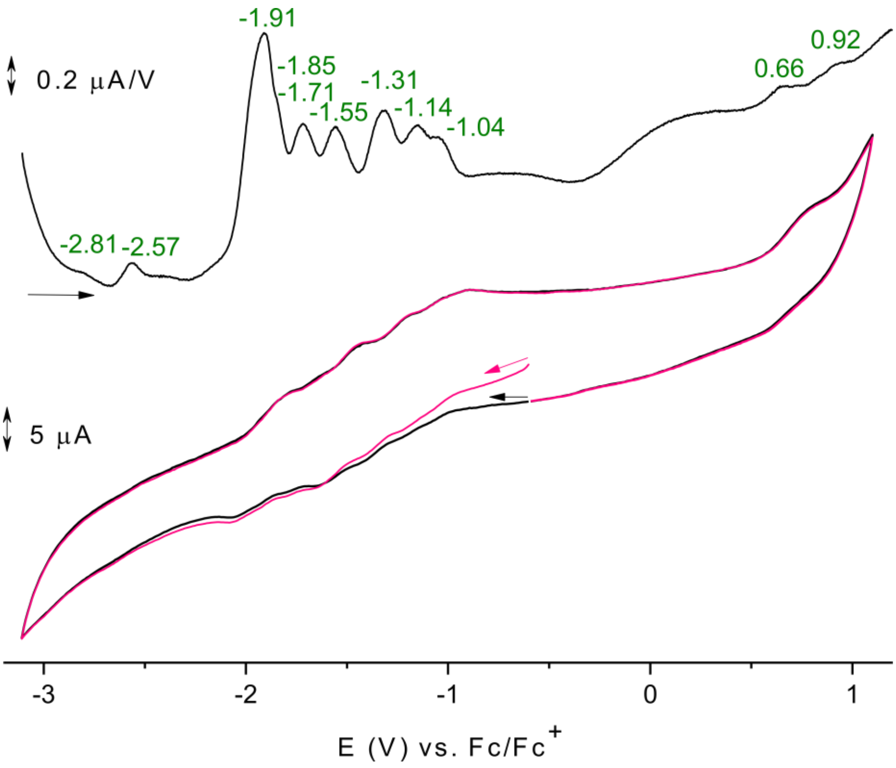

Figure S18. Cyclic voltammogram (bottom) and differential pulse voltammogram (top) recorded for compound **2** (THF, $[Bu_4N]PF_6$, glassy-carbon electrode, 100 mV/s).

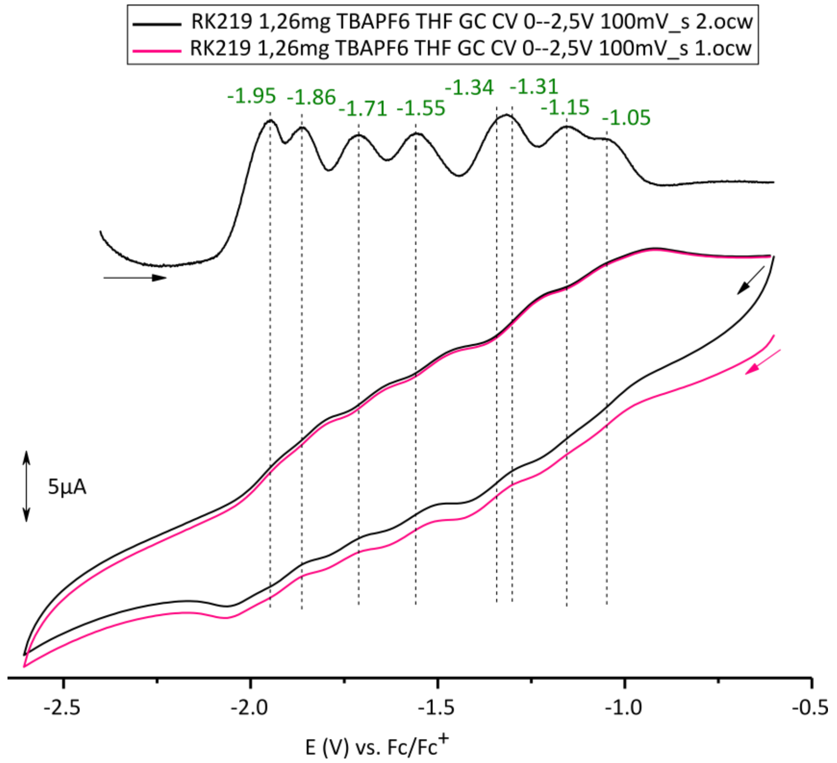

Figure S19. Cyclic voltammogram (bottom) and differential pulse voltammogram (top) recorded for compound **2** (THF, $[Bu_4N]PF_6$, glassy-carbon electrode, 100 mV/s).

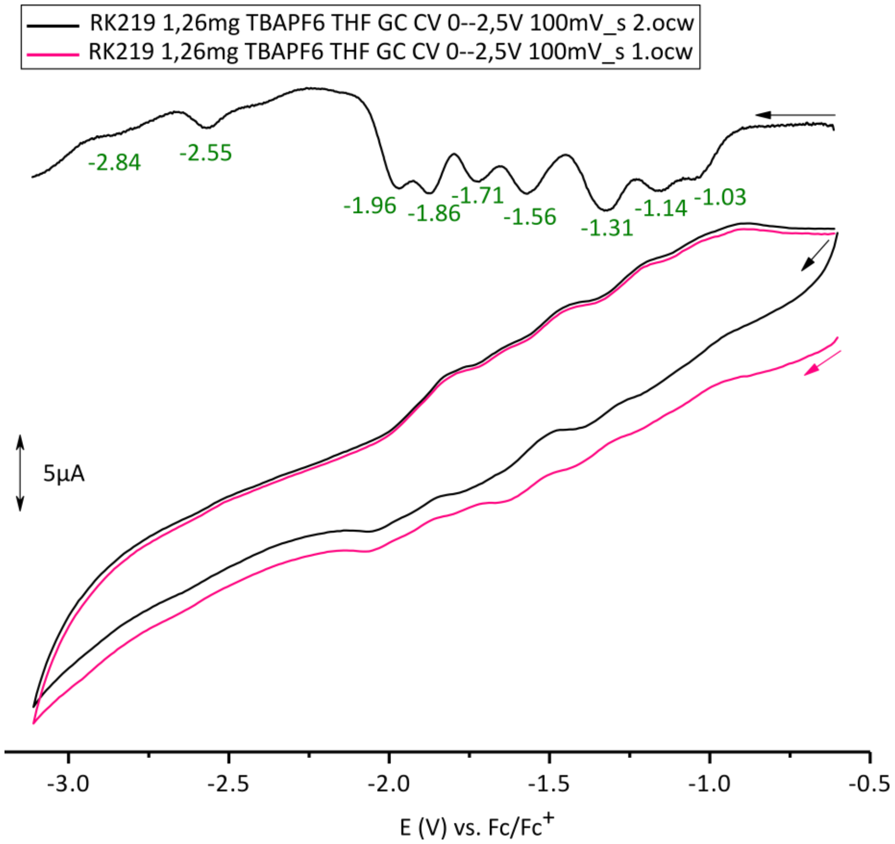

Figure S20. Cyclic voltammogram (bottom) and differential pulse voltammogram (top) recorded for compound **2** (THF, $[Bu_4N]PF_6$, glassy-carbon electrode, 100 mV/s).

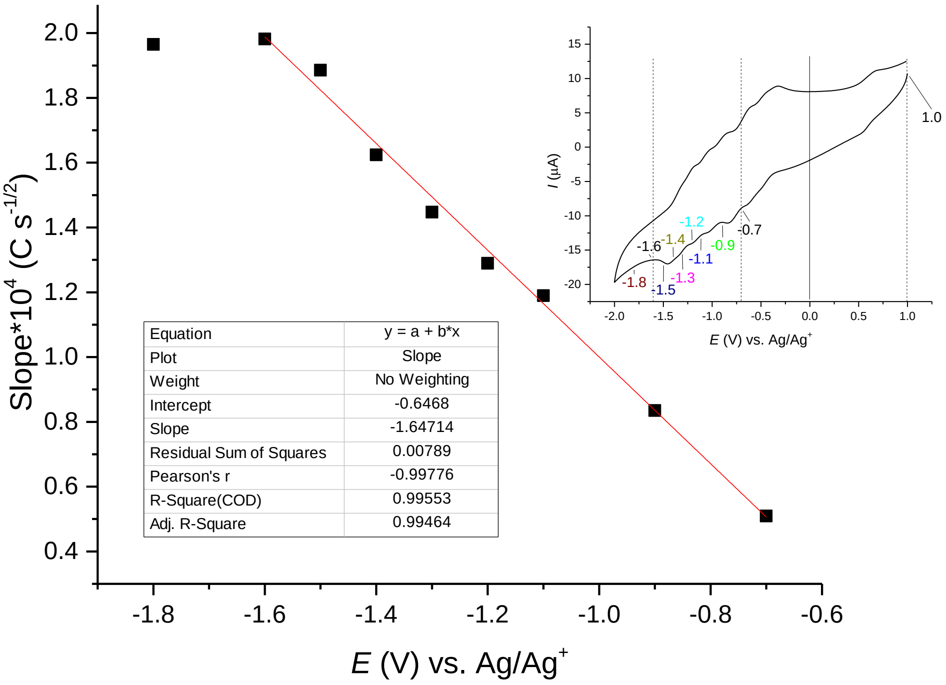

Figure S21. Chronocoulometric data obtained for compound **2** (THF, $[Bu_4N]PF_6$, glassy-carbon electrode, 100 mV/s).

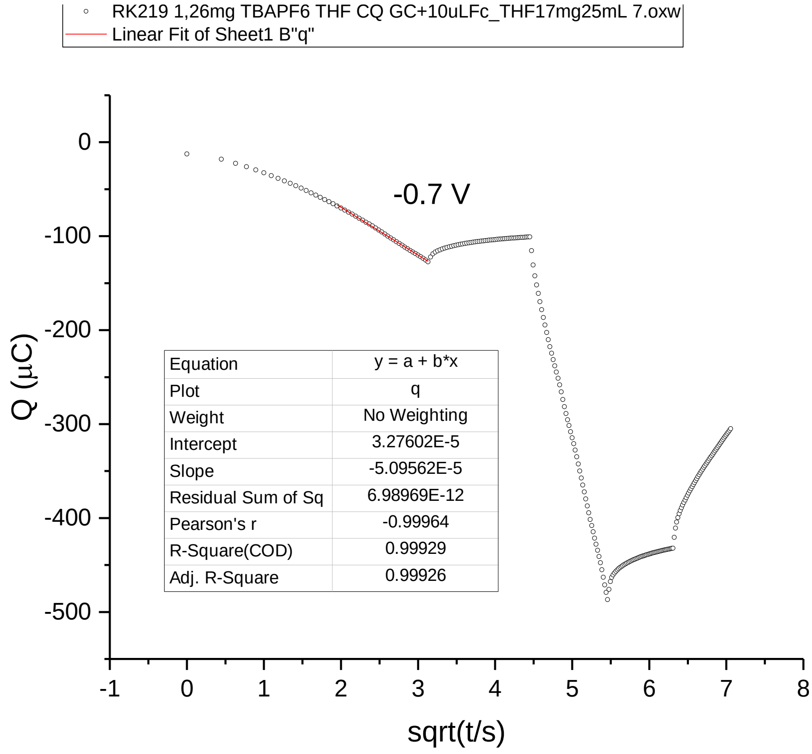

Figure S22. An example chronocoulometric experiment for compound **2** (@–0.7 V) presented as an Anson plot along with least-square fitting.

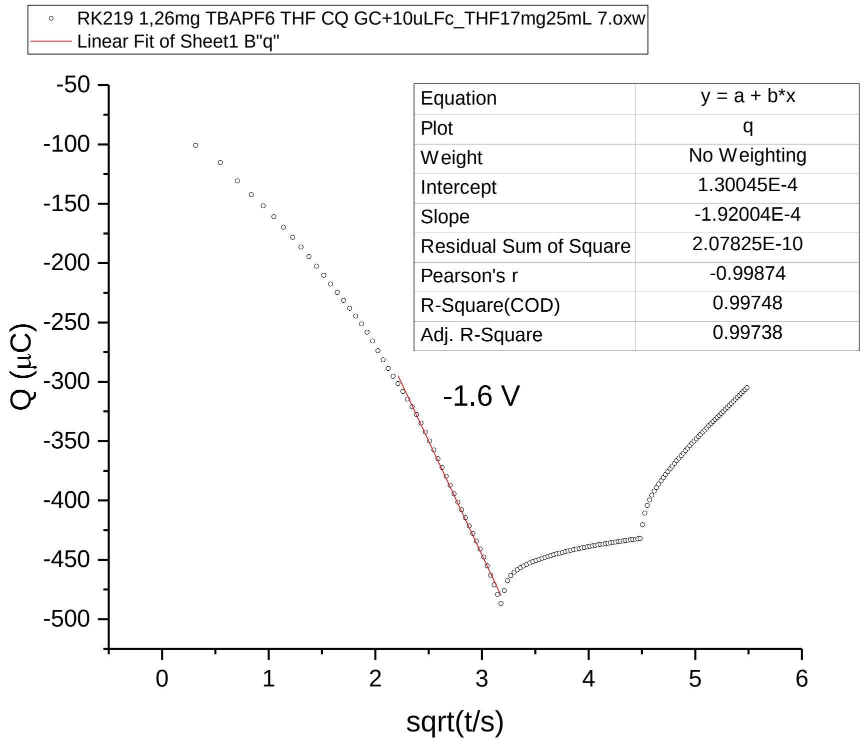

Figure S23. An example chronocoulometric experiment for compound **2** (@–1.6 V) presented as an Anson plot along with least-square fitting.

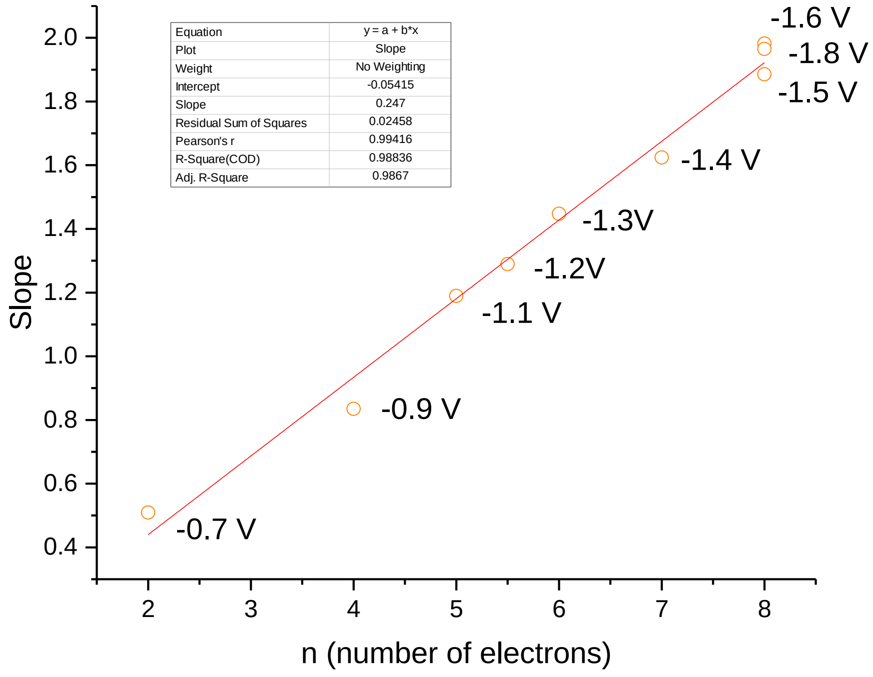

Figure S24. Absolute values of Anson equation slope for reductions of **2** (THF, GC, $TBAPF_6$, Ag/Ag^+, Pt) at specified potentials assuming that totally 8-electron transfer was observed in the potential region

from –2 to 0 V (vs. Ag/Ag^{+}), i.e. that the reduction @1.32 V (vs. Fc$^{0/+}$) observed @–0.9 V (vs.Ag$^{0/+}$) in chronocoulometric experiment is a two-electron process (3rd and 4th electrons)..

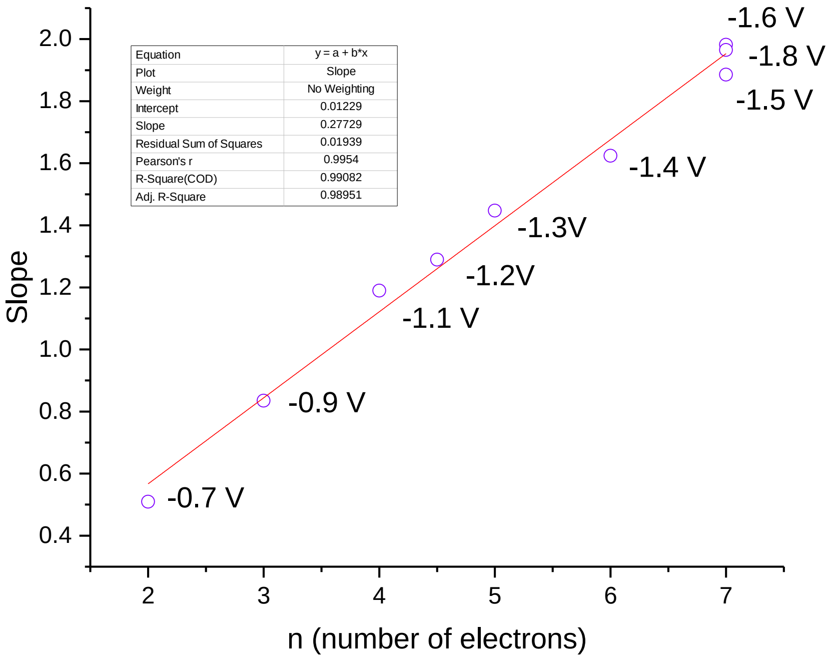

Figure S25. Absolute values of Anson equation slope for reductions of **2** (THF, GC, $TBAPF_6$, Ag/Ag^{+}, Pt) at specified potentials assuming that totally 7-electron transfer was observed in the potential region from –2 to 0 V (vs. Ag/Ag^{+}), i.e. that the reduction @1.32 V (vs. Fc$^{0/+}$) observed @–0.9 V (vs.Ag$^{0/+}$) in chronocoulometric experiment is a one-electron process (3rd electron).

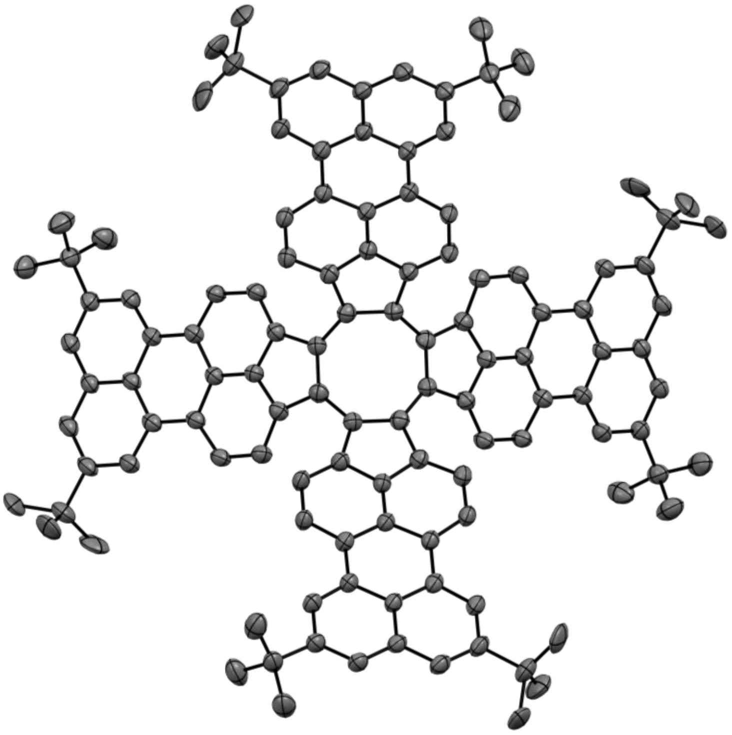

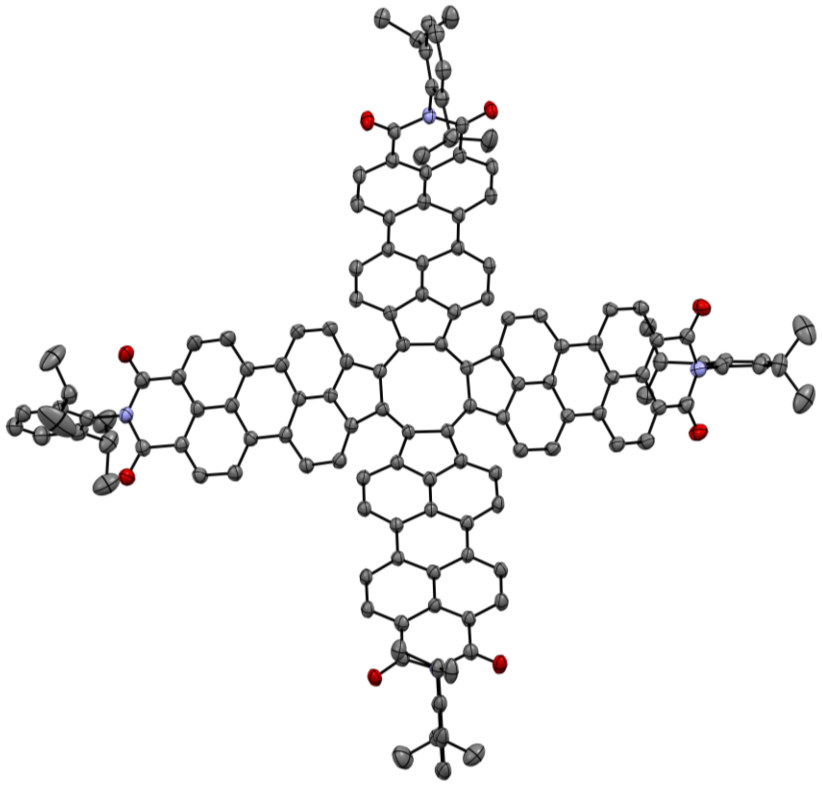

Figure S26. Top: Crystal structure of **1** with thermal ellipsoids shown at the 30% probability level. Solvent molecules, hydrogen atoms, and the other symmetry-independent molecule of **1** were removed for clarity. Bottom: Crystal structure of **2** with thermal ellipsoids shown at the 30% probability level. Solvent molecules, hydrogen atoms, and the other symmetry-independent molecule of **2** were removed for clarity.

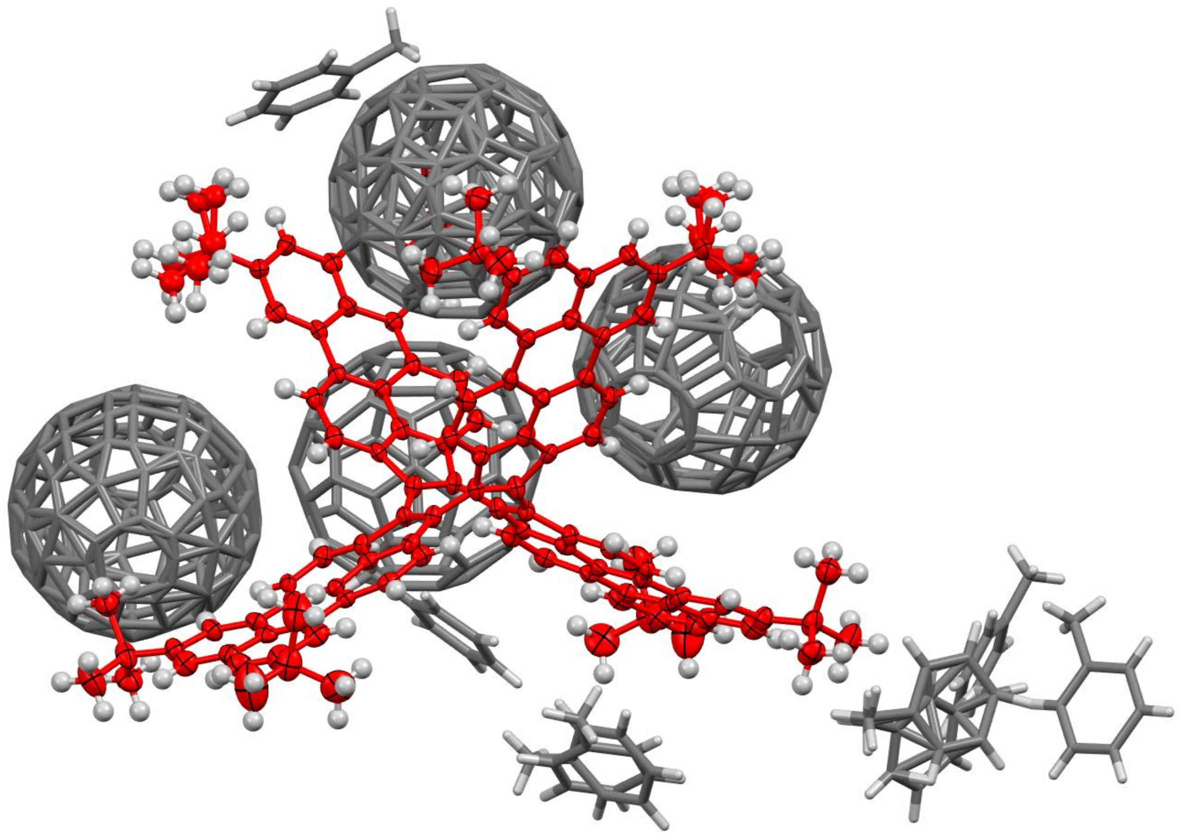

Figure S27. Crystal structure of **1**·5C_{60}·$n$$C_7H_8$. The molecule of **1** (red) is shown with thermal ellipsoids at the 50% probability level. Fullerene and solvent molecules are shown as sticks.

Additional Tables

Table S1. Crystal data and structure refinement for **1** (CCDC 2381163).

Identification code	rk275ba
Empirical formula	$C_{156}H_{188}$
Formula weight	2063.05
Temperature/K	80(2)
Crystal system	tetragonal
Space group	$I4_1/acd$
a/Å	21.398(3)
b/Å	21.398(3)
c/Å	53.967(14)
α/°	90
β/°	90
γ/°	90
Volume/Å^3	24710(9)
Z	8
ρ_{calc}g/cm^3	1.109
μ/mm^{-1}	0.062
F(000)	8992.0
Crystal size/mm^3	0.15 × 0.12 × 0.04
Radiation	Mo Kα (λ = 0.71073)
2Θ range for data collection/°	3.806 to 55.996
Index ranges	-28 ≤ h ≤ 28, -28 ≤ k ≤ 28, -71 ≤ l ≤ 71
Reflections collected	194560
Independent reflections	7461 [R_{int} = 0.1481, R_{sigma} = 0.0372]
Data/restraints/parameters	7461/53/404
Goodness-of-fit on F^2	1.614
Final R indexes [I>=2σ (I)]	R_1 = 0.1132, wR_2 = 0.2734
Final R indexes [all data]	R_1 = 0.1883, wR_2 = 0.2976
Largest diff. peak/hole / e Å^{-3}	0.46/-0.52

Table S2. Crystal data and structure refinement for **2** (CCDC 2381426).

Identification code	rk245t
Empirical formula	$C_{242}H_{212}N_4O_8$
Formula weight	3304.14
Temperature/K	100(2)
Crystal system	triclinic
Space group	P-1
a/Å	16.846(2)
b/Å	22.876(6)
c/Å	25.515(4)
α/°	69.30(2)
β/°	84.33(2)
γ/°	76.83(2)
Volume/Å^3	8954(3)
Z	2
ρ_{calc}g/cm^3	1.225
μ/mm^{-1}	0.559
F(000)	3512.0
Crystal size/mm^3	0.34 × 0.18 × 0.02
Radiation	Cu Kα (λ = 1.54184)
2Θ range for data collection/°	3.702 to 147.834
Index ranges	-20 ≤ h ≤ 20, -28 ≤ k ≤ 28, -31 ≤ l ≤ 31
Reflections collected	55865
Independent reflections	55865 [R_{int} = ?, R_{sigma} = 0.0374]
Data/restraints/parameters	55865/25/1880
Goodness-of-fit on F^2	1.246
Final R indexes [I>=2σ (I)]	R_1 = 0.1195, wR_2 = 0.3290
Final R indexes [all data]	R_1 = 0.1709, wR_2 = 0.3687
Largest diff. peak/hole / e Å^{-3}	0.77/-0.54

Table S3. Crystal data and structure refinement for **1**·$5C_{60}$·nC_7H_8 (CCDC 2381427).

Identification code	rk302ba
Empirical formula	C470.4H161.6
Formula weight	5812.39
Temperature/K	100(2)
Crystal system	hexagonal
Space group	P6522
a/Å	37.776(9)
b/Å	37.776(9)
c/Å	37.0120(12)
α/°	90
β/°	90
γ/°	120
Volume/Å^3	45741(22)
Z	6
ρ_{calc} g/cm^3	1.266
μ/mm^{-1}	0.556
F(000)	17904.0
Crystal size/mm^3	0.18 × 0.17 × 0.15
Radiation	Cu Kα (λ = 1.54184)
2Θ range for data collection/°	3.604 to 146.832
Index ranges	-41 ≤ h ≤ 46, -46 ≤ k ≤ 38, -42 ≤ l ≤ 45
Reflections collected	394855
Independent reflections	30067 [Rint = 0.0451, Rsigma = 0.0248]
Data/restraints/parameters	30067/1743/1857
Goodness-of-fit on F2	3.352
Final R indexes [I>=2σ (I)]	R1 = 0.1680, wR2 = 0.4278
Final R indexes [all data]	R1 = 0.1921, wR2 = 0.4423
Largest diff. peak/hole / e Å-3	0.97/-0.68
Flack parameter	0.12(17)

Table S4. Data for optimized geometries of fused cyclooctatetraenes discussed in this work (GD3BJ/CAM-B3LYP/6-31G(d,p)).

Name[a]	Formula	n[b]	m[b]	SCF E[c] a.u.	ZPV[d] a.u.	LVF[e] cm^{-1}	E[f] a.u.	H[f] a.u.	G[f] a.u.
COT	C_8H_8	–2	1	–309.253240	0.127716	209.49	–309.118855	–309.117911	–309.155870
		–2	3	–309.137170	0.120621	138.54	–309.008560	–309.007616	–309.049165
		–1	2	–309.422940	0.132156	103.83	–309.284170	–309.283226	–309.322132
		0	1	–309.417790	0.134573	183.42	–309.276575	–309.275631	–309.313626
		2	1	–308.680542	0.135753	27.01	–308.537492	–308.536548	–308.578432
		2	3	–308.620926	0.130948	159.24	–308.482964	–308.482020	–308.521824
T	$C_{48}H_{24}$	–2	1	–1842.670622	0.557688	31.22	–1842.081862	–1842.080918	–1842.172702
		–2	3	–1842.661510	0.557820	30.78	–1842.072468	–1842.071523	–1842.164773
		–1	2	–1842.730275	0.562613	32.56	–1842.136772	–1842.135828	–1842.228204
		0	1	–1842.683894	0.567165	28.63	–1842.085902	–1842.084958	–1842.177136
		1	2	–1842.464163	0.567295	30.99	–1841.866199	–1841.865255	–1841.957254
		2	1	–1842.136118	0.567360	25.03	–1841.538158	–1841.537213	–1841.628192
		2	3	–1842.113927	0.566184	29.71	–1841.516850	–1841.515906	–1841.608886
TI	$C_{56}H_{20}N_4O_8$	–8	1	–2964.175625	0.611082	19.92	–2963.519296	–2963.518352	–2963.639768
		–2	1	–2965.853189	0.626674	17.08	–2965.181559	–2965.180614	–2965.303639
		–2	3	–2965.845778	0.627563	15.97	–2965.173195	–2965.172250	–2965.296706
		–1	2	–2965.837628	0.629613	16.65	–2965.163029	–2965.162085	–2965.286260
		0	1	–2965.731385	0.632937	14.76	–2965.053405	–2965.052461	–2965.176804
		1	2	–2965.470435	0.632045	16.74	–2964.793430	–2964.792486	–2964.916762
		2	1	–2965.109604	0.631151	18.03	–2964.433488	–2964.432544	–2964.555968
		2	3	–2965.086396	0.629804	16.19	–2964.411320	–2964.410376	–2964.535919
P	$C_{88}H_{40}$	–2	1	–3376.046614	0.989956	10.71	–3375.000746	–3374.999802	–3375.146503
		–2	3	–3376.043870	0.990409	10.49	–3374.997503	–3374.996558	–3375.144489
		–1	2	–3376.060377	0.993493	10.89	–3375.011087	–3375.010143	–3375.157651
		0	1	–3375.998222	0.999023	9.94	–3374.943546	–3374.942601	–3375.089789
		1	2	–3375.797004	0.999137	11.10	–3374.742394	–3374.741449	–3374.888239
		2	1	–3375.513513	0.999220	11.48	–3374.458870	–3374.457926	–3374.603552
		2	3	–3375.500466	0.998031	10.88	–3374.446843	–3374.445898	–3374.593089
PI	$C_{96}H_{36}N_4O_8$	–8	1	–4498.102624	1.042813	9.11	–4496.989808	–4496.988864	–4497.164311
		–2	1	–4499.203787	1.059316	7.06	–4498.074809	–4498.073865	–4498.251532
		–2	3	–4499.201660	1.059884	6.94	–4498.072088	–4498.071143	–4498.250019
		–1	2	–4499.168743	1.060720	7.11	–4498.038264	–4498.037320	–4498.216274
		0	1	–4499.068646	1.065540	6.56	–4497.933399	–4497.932455	–4498.111415
		1	2	–4498.838231	1.065001	7.23	–4497.703645	–4497.702701	–4497.881421
		2	1	–4498.528576	1.064404	7.41	–4497.394598	–4497.393653	–4497.571333
		2	3	–4498.514592	1.062777	7.09	–4497.382026	–4497.381082	–4497.560435

[a] Structure code (T = **TC** with R = H, TI = **TCTI** with R = H, P = **1H**, PI = **2H** in Figure 1). [b] System charge (*n*) and multiplicity (*m*). Optimized geometries are provided as xyz files, named “code(*n*,*m*).xyz”. [c] Self-consistent field electronic energy. [d] Zero-point vibrational energy. [e] Lowest vibrational frequency. [f] Thermodynamic functions.

NMR Spectra

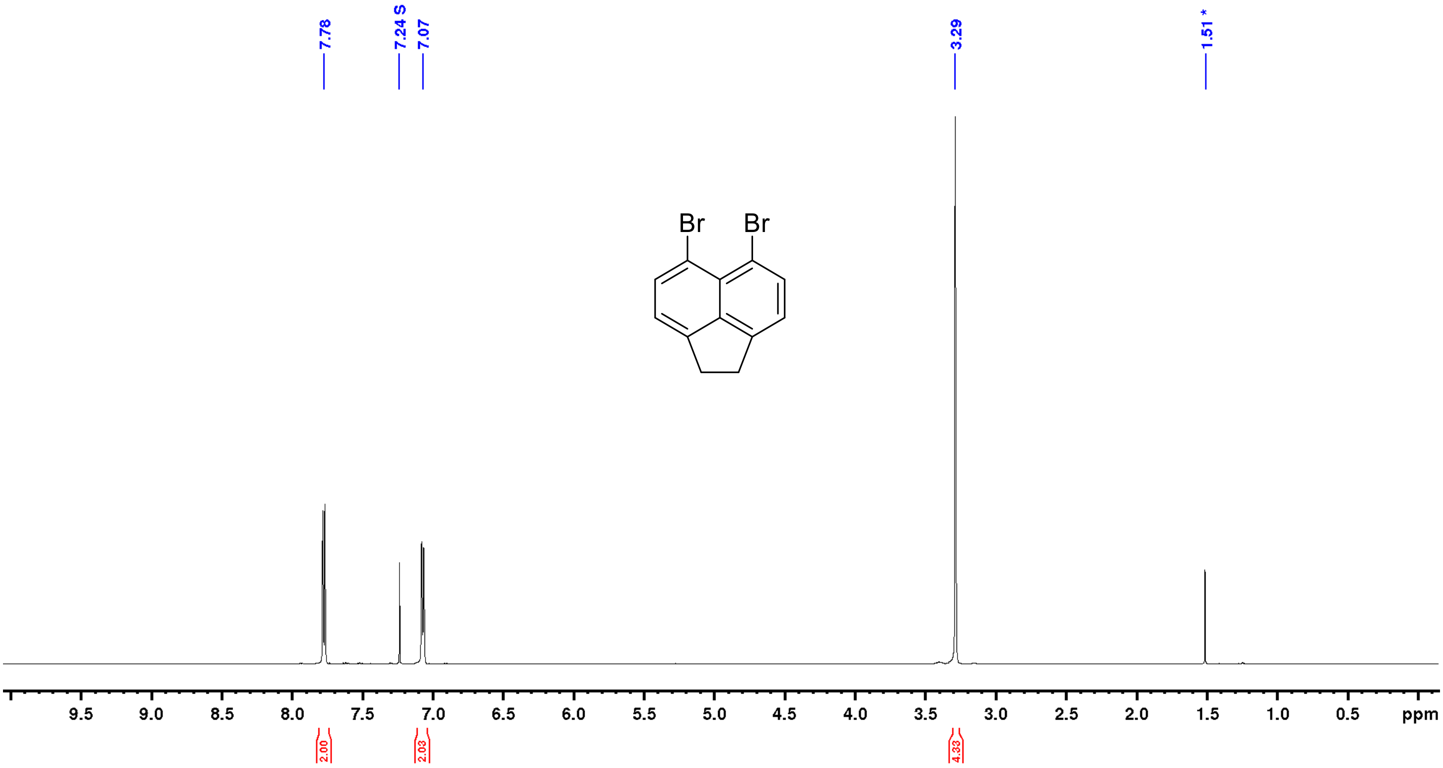

Figure S27. ^{1}H NMR spectrum of **S2** (500 MHz, chloroform-*d* , 300 K).

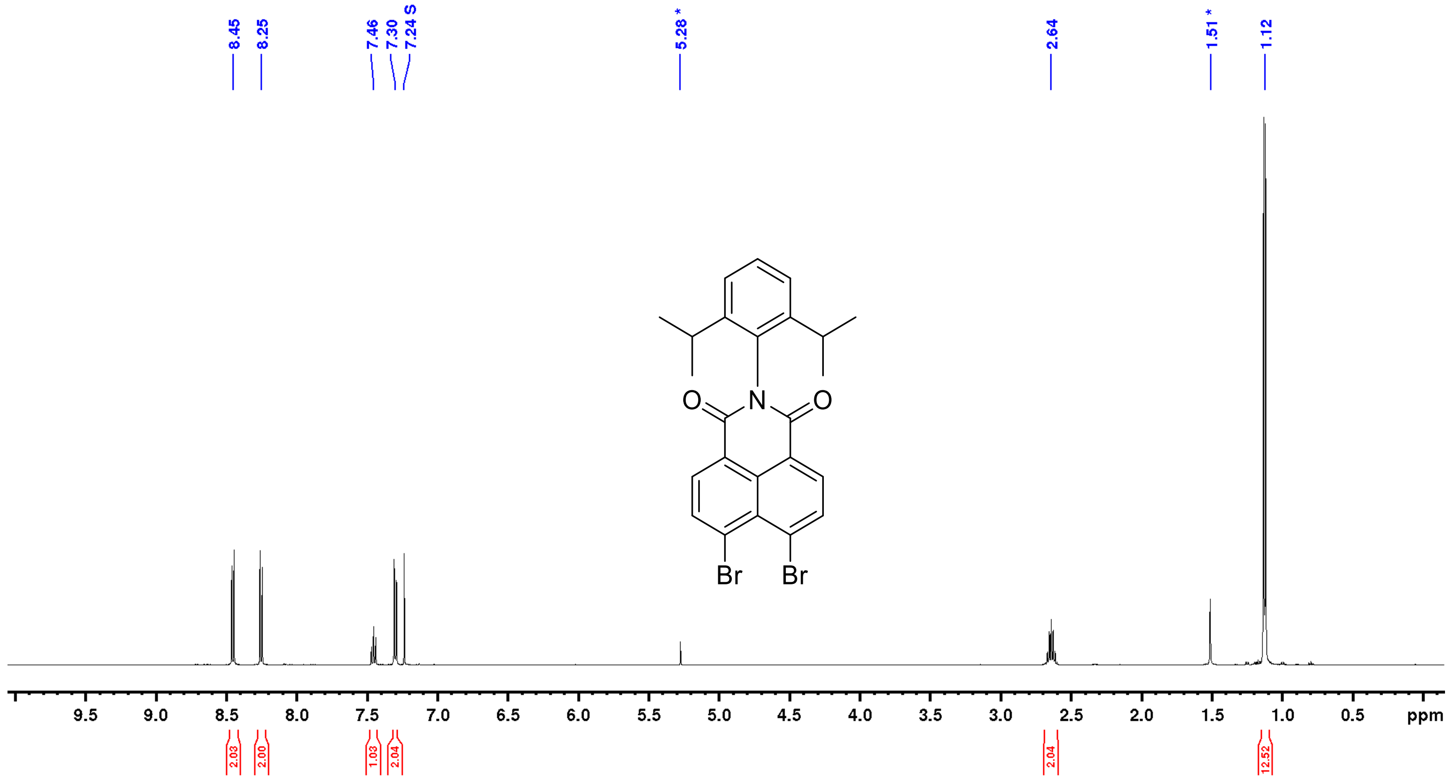

Figure S28. ^{1}H NMR spectrum of **S4** (500 MHz, chloroform-*d* , 300 K).

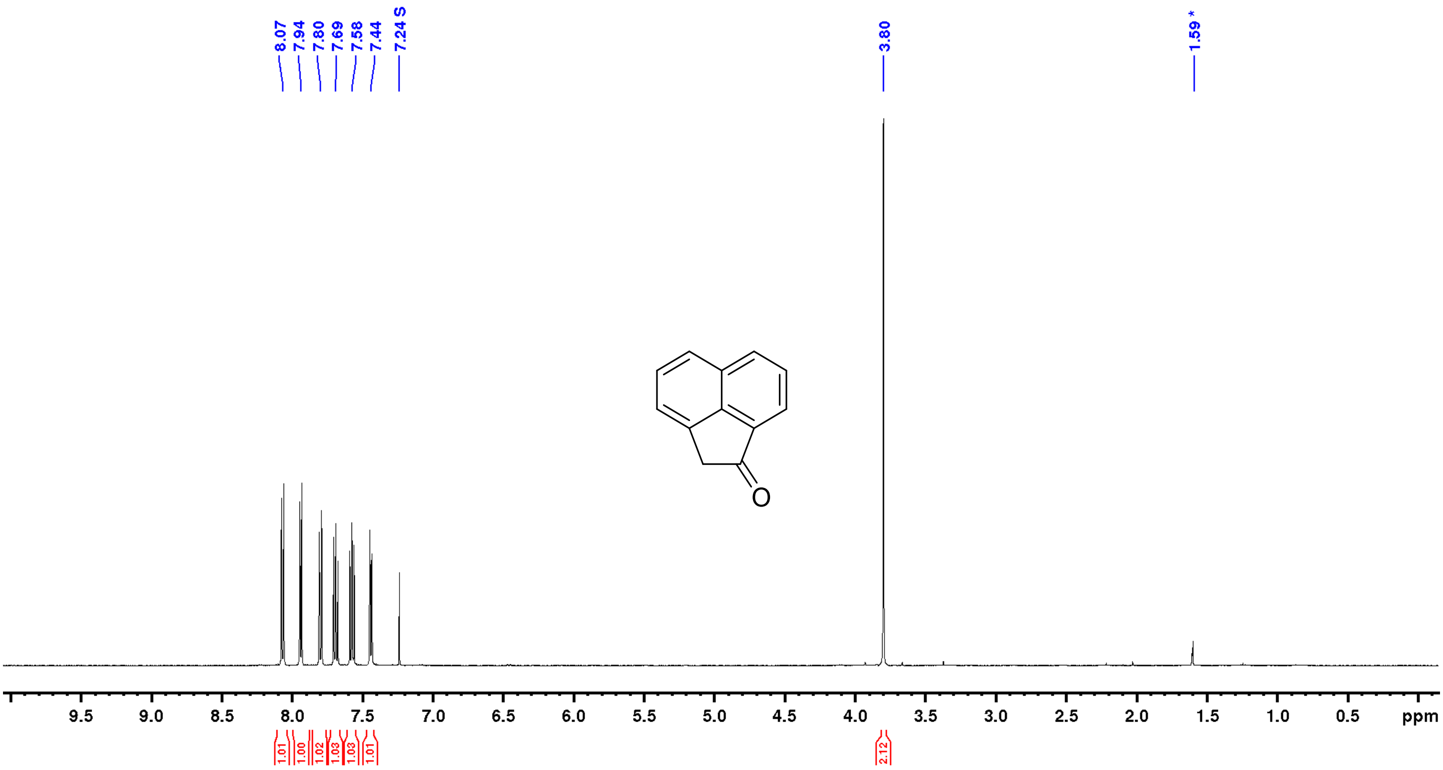

Figure S29. ^{1}H NMR spectrum of **S5** (500 MHz, chloroform-*d* , 300 K).

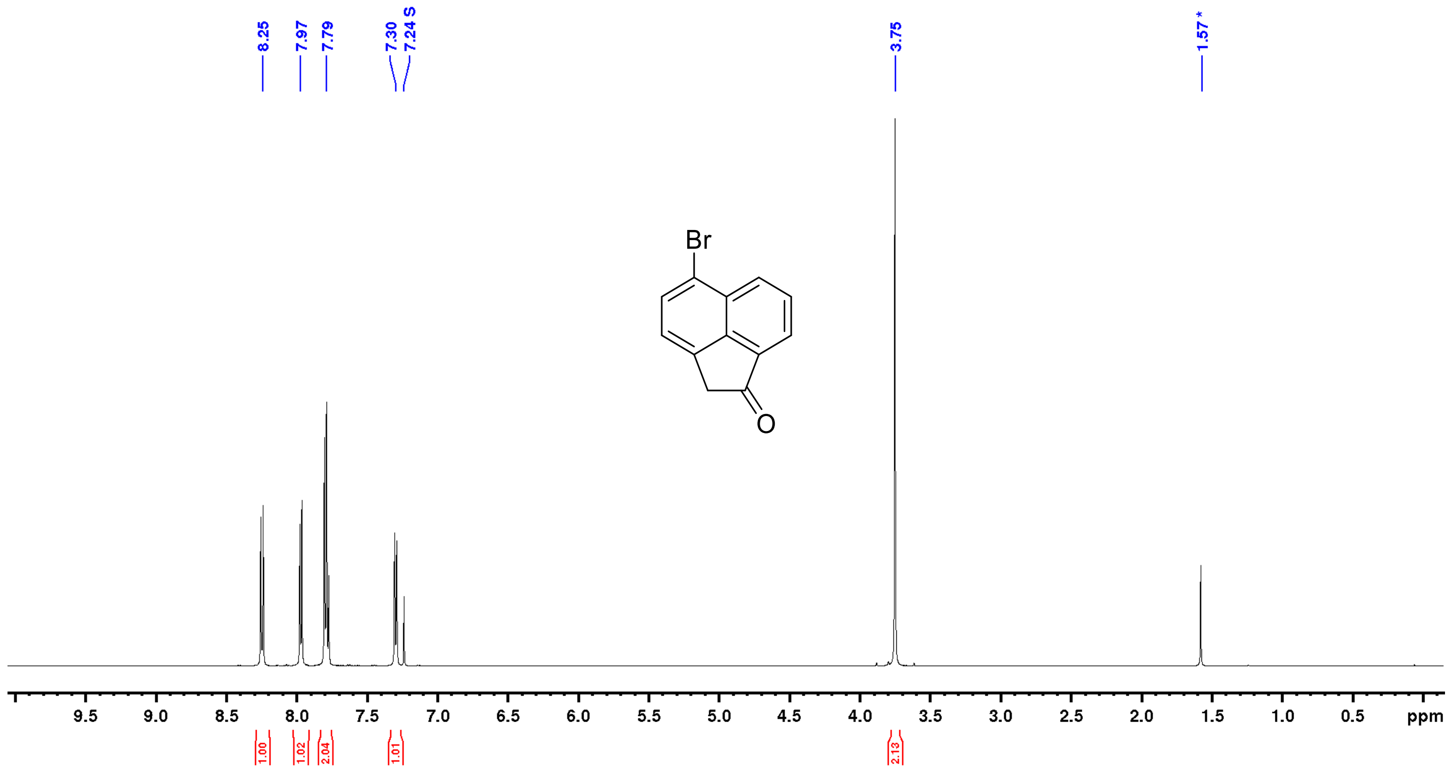

Figure S30. ^{1}H NMR spectrum of **3** (500 MHz, chloroform-*d* , 300 K).

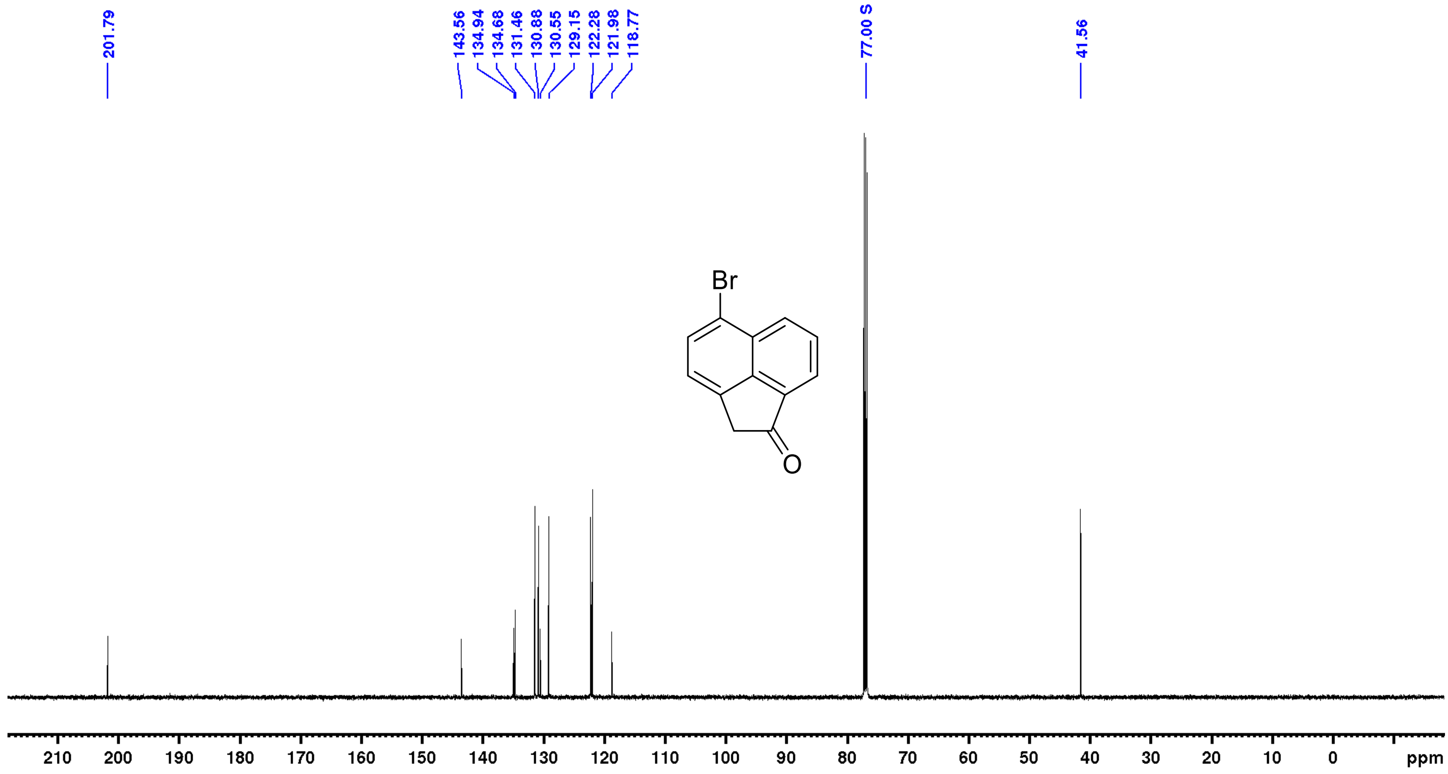

Figure S31. ^{13}C NMR spectrum of **3** (125 MHz, chloroform-*d* , 300 K).

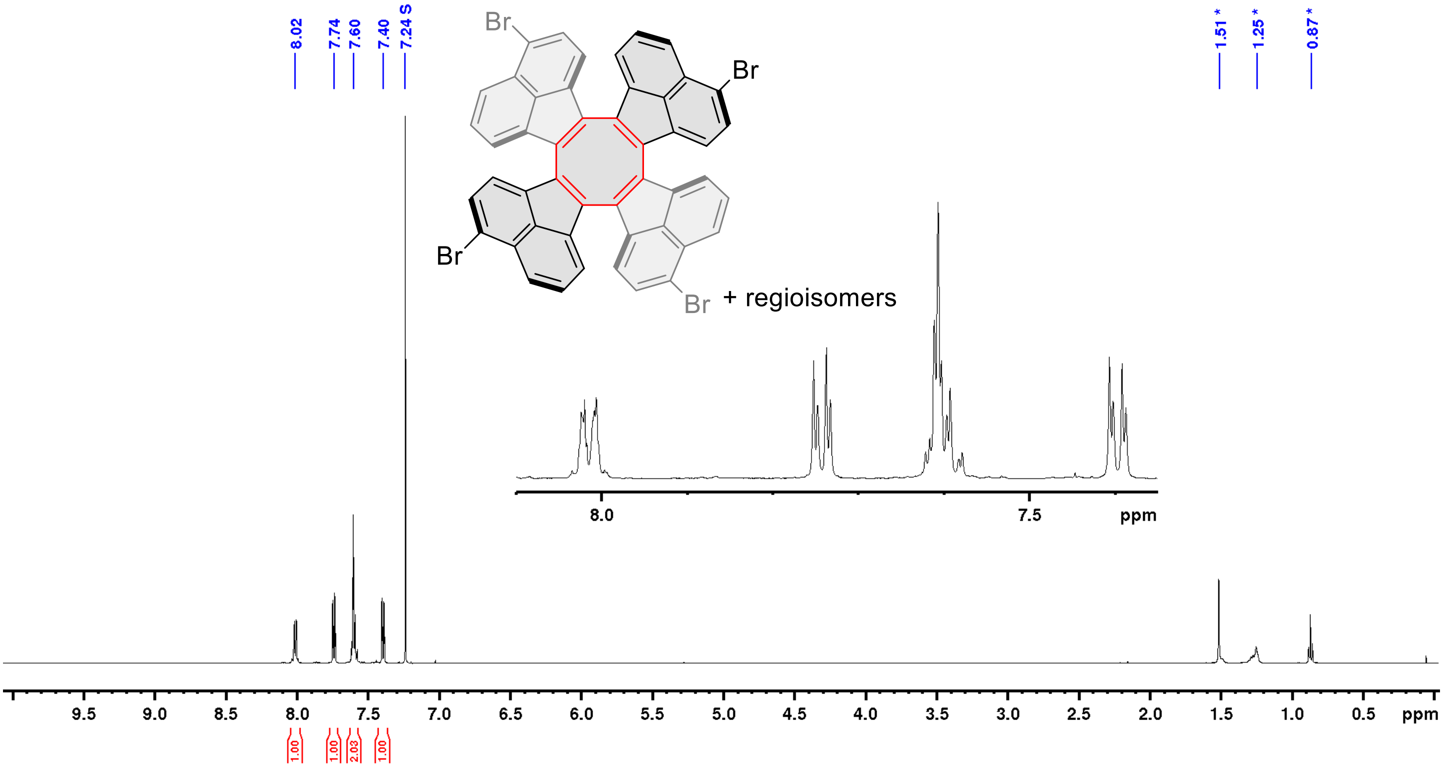

Figure S32. ^{1}H NMR spectrum of **4** (500 MHz, chloroform-*d* , 300 K).

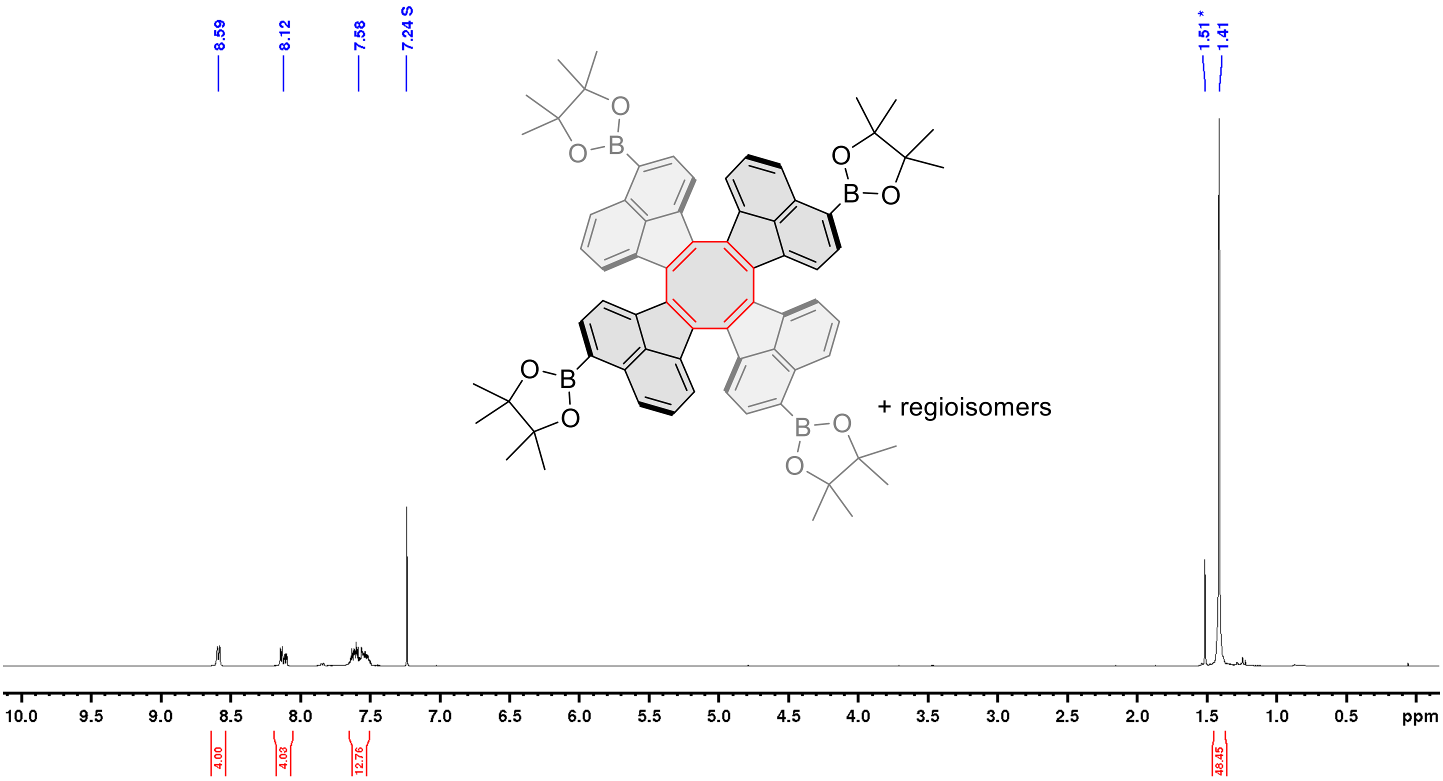

Figure S33. ^{1}H NMR spectrum of **5** (500 MHz, chloroform-*d* , 300 K).

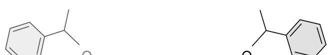

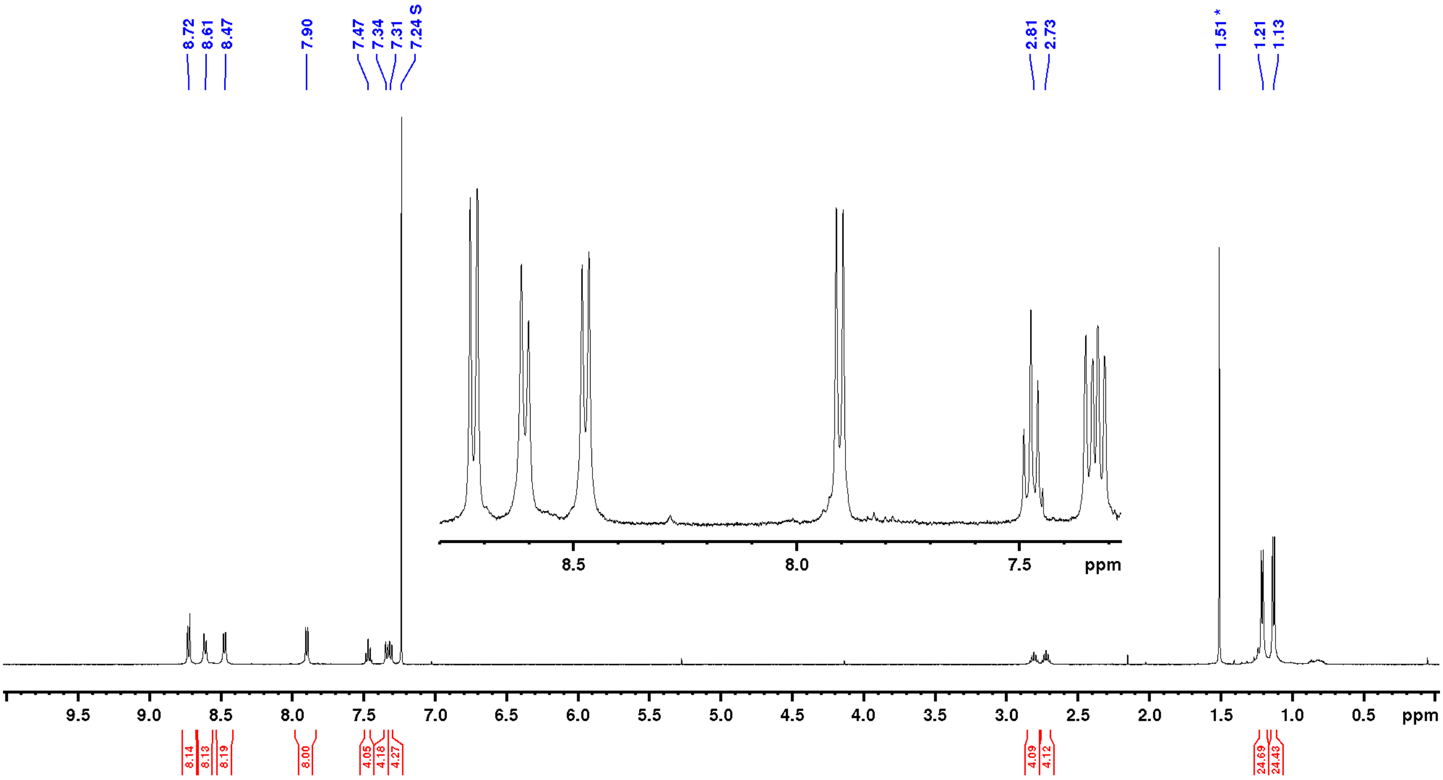

Figure S34. ^{1}H NMR spectrum of **2** (500 MHz, chloroform-*d* , 300 K).

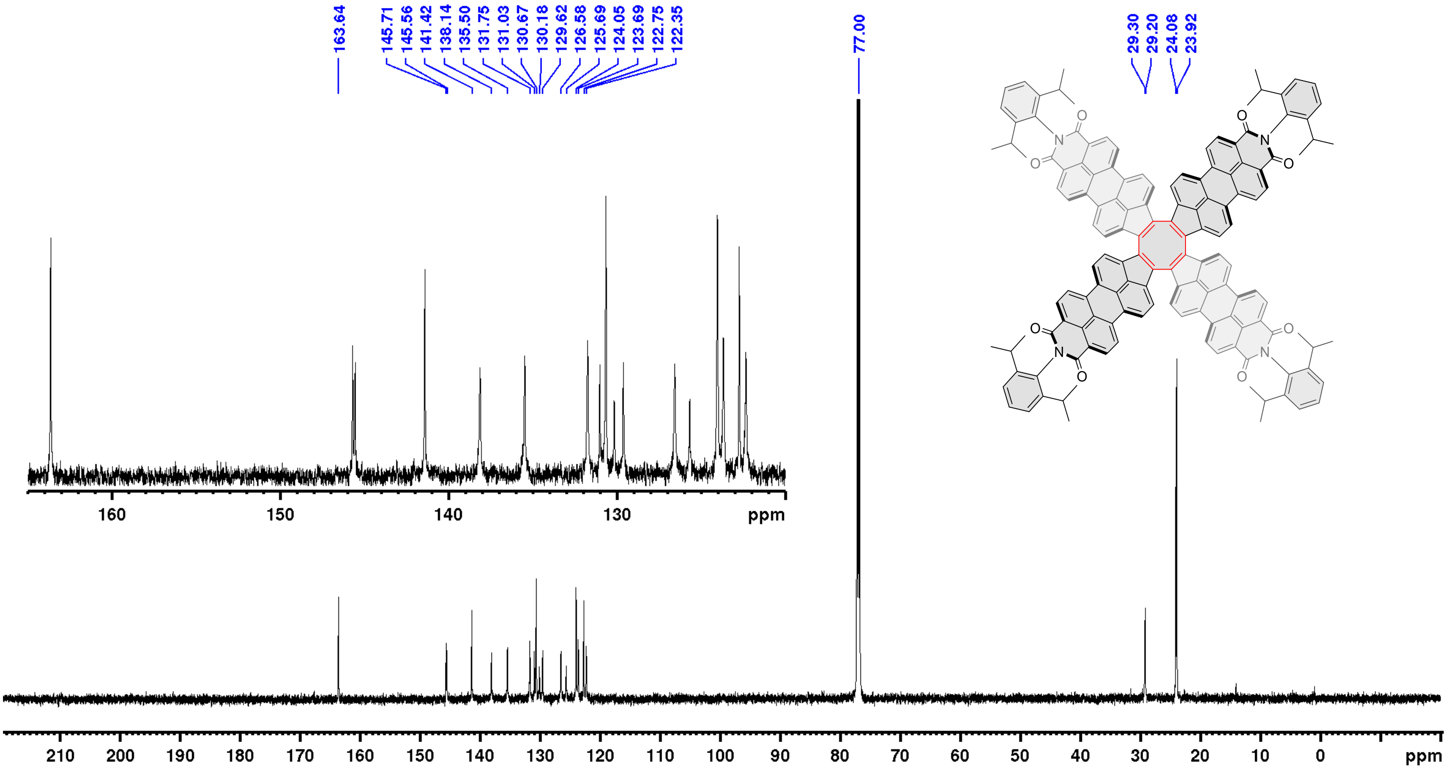

Figure S35. ^{13}C NMR spectrum of **2** (151 MHz, chloroform-*d* , 300 K).

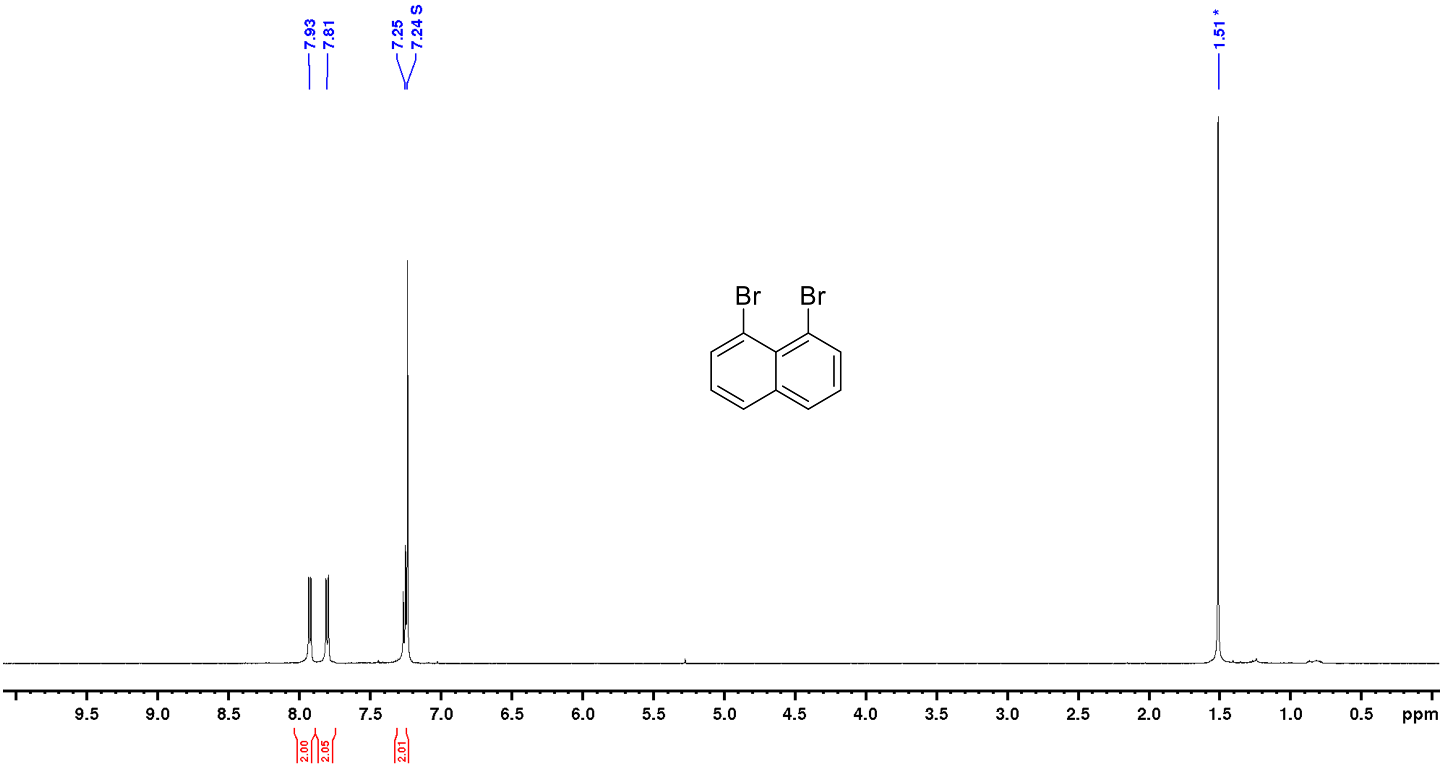

Figure S36. ^{1}H NMR spectrum of **S5** (500 MHz, chloroform-*d* , 300 K).

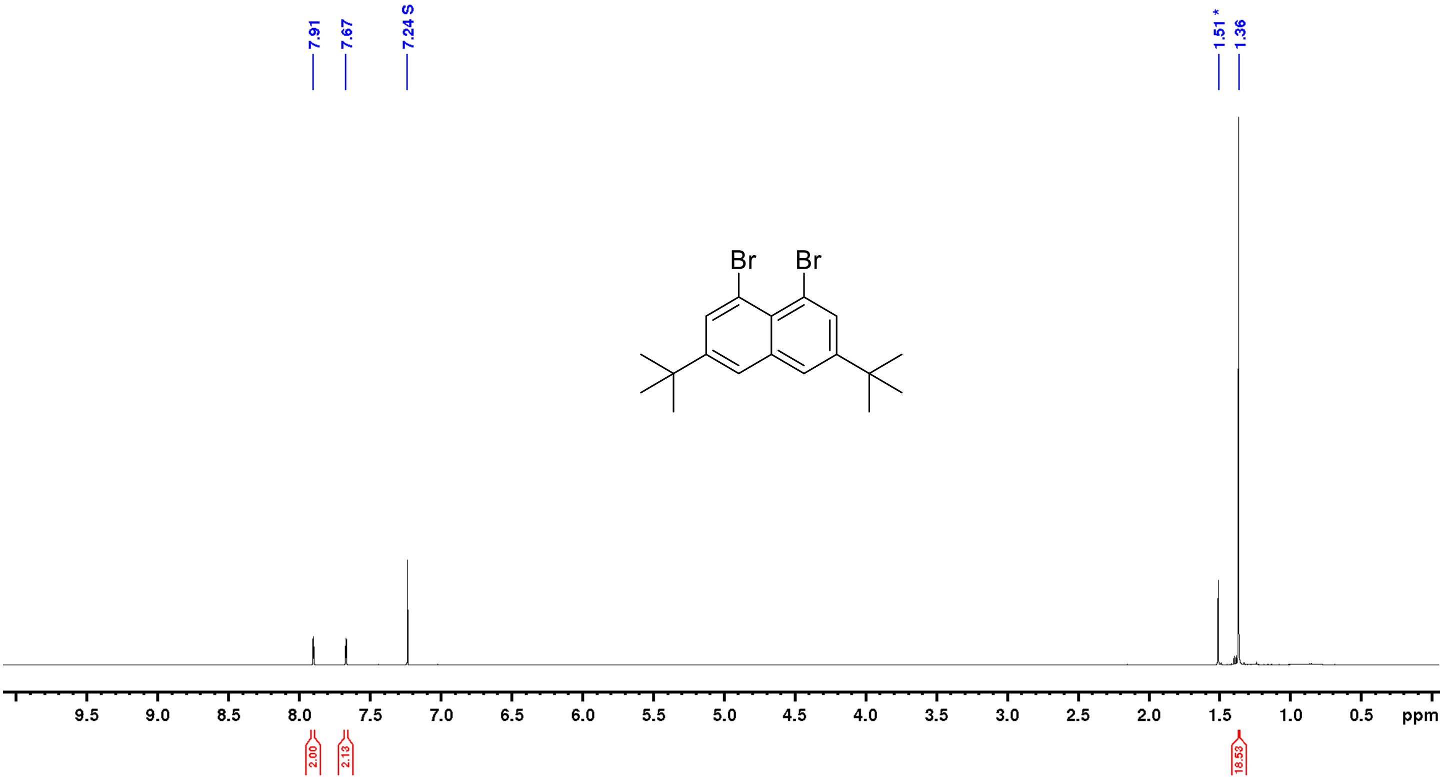

Figure S37. ^{1}H NMR spectrum of **6** (500 MHz, chloroform-*d* , 300 K).

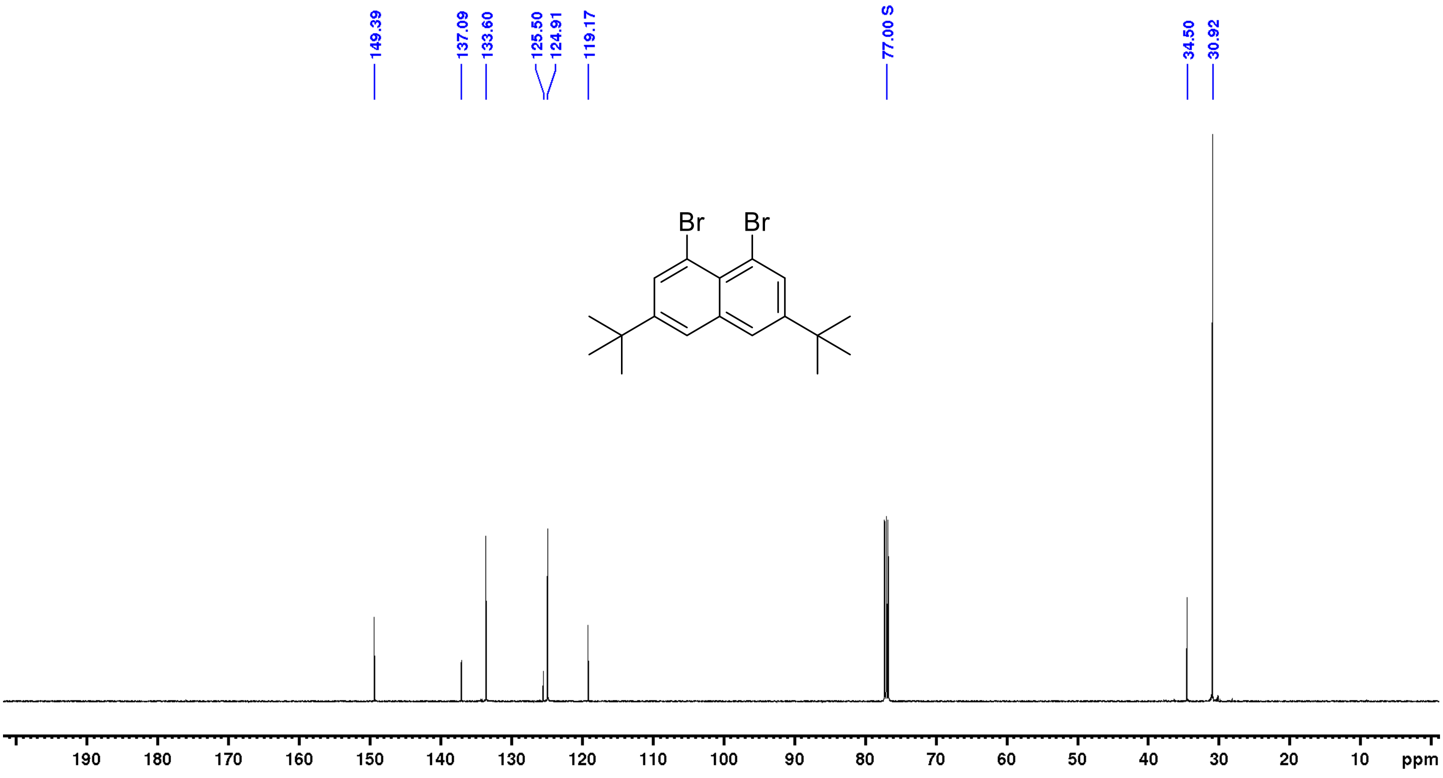

Figure S38. ^{13}C NMR spectrum of **6** (125 MHz, chloroform-*d* , 300 K).

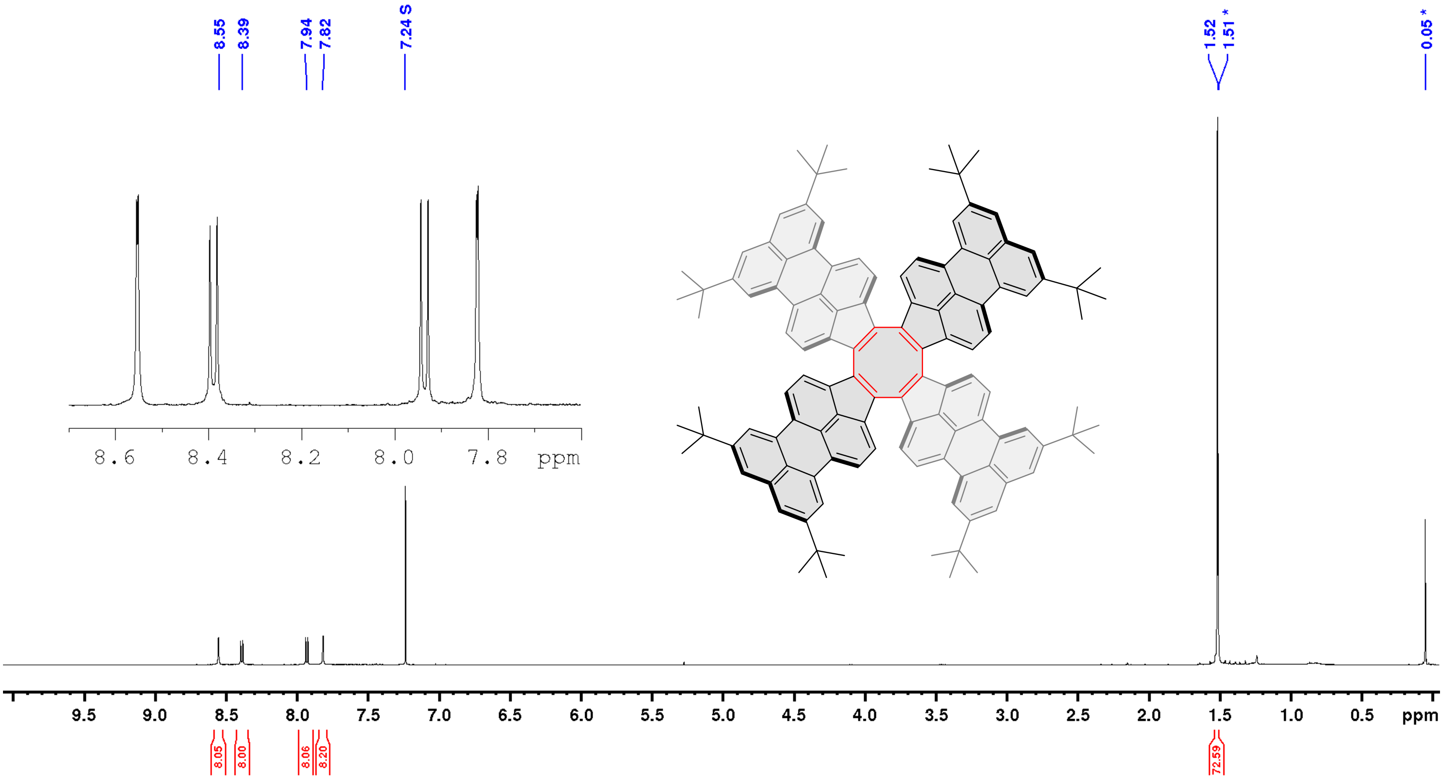

Figure S39. ^{1}H NMR spectrum of **1** (500 MHz, chloroform-*d* , 300 K).

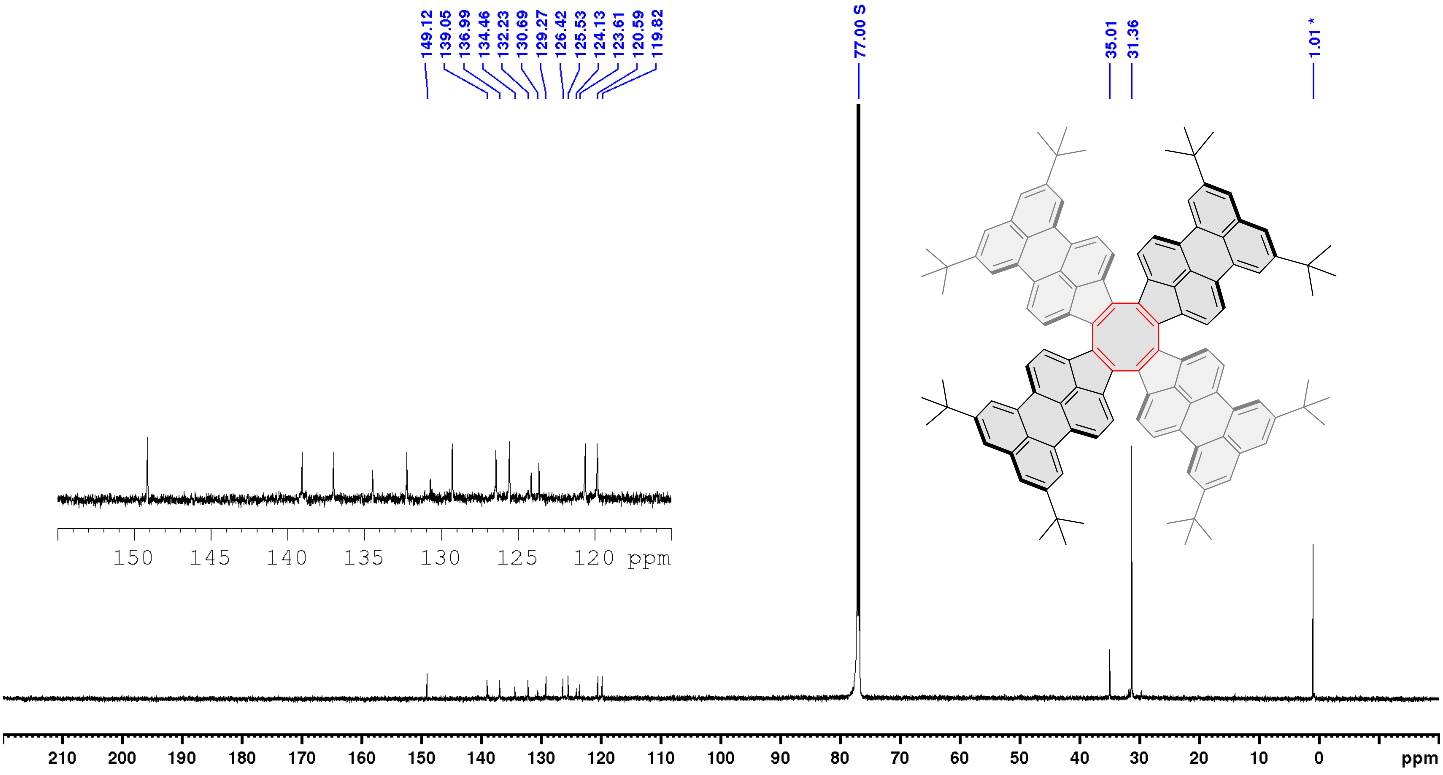

Figure S40. ^{13}C NMR spectrum of **1** (151 MHz, chloroform-*d* , 300 K).

Mass Spectra

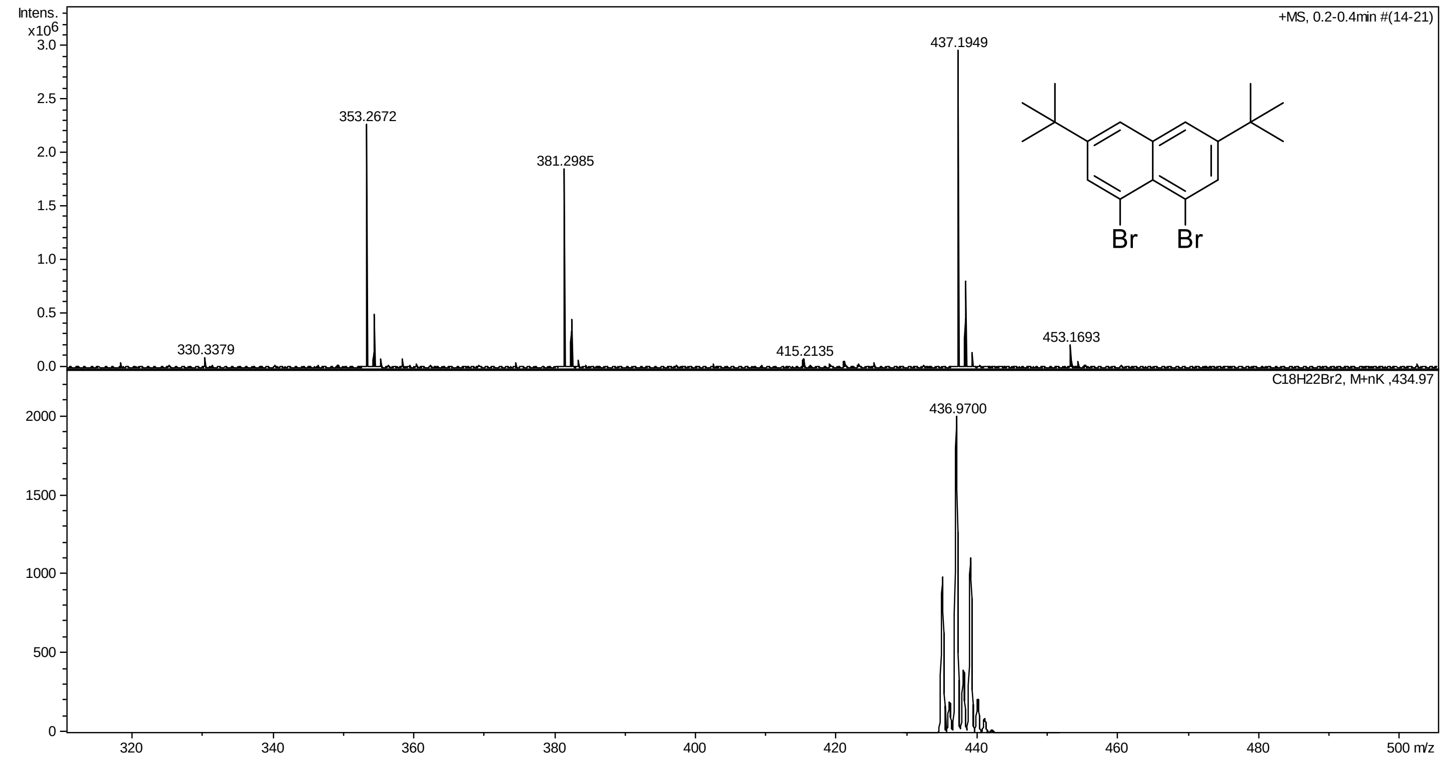

Figure S41. High resolution mass spectrum of **6** (ESI–TOF, top: experimental, bottom: simulated).

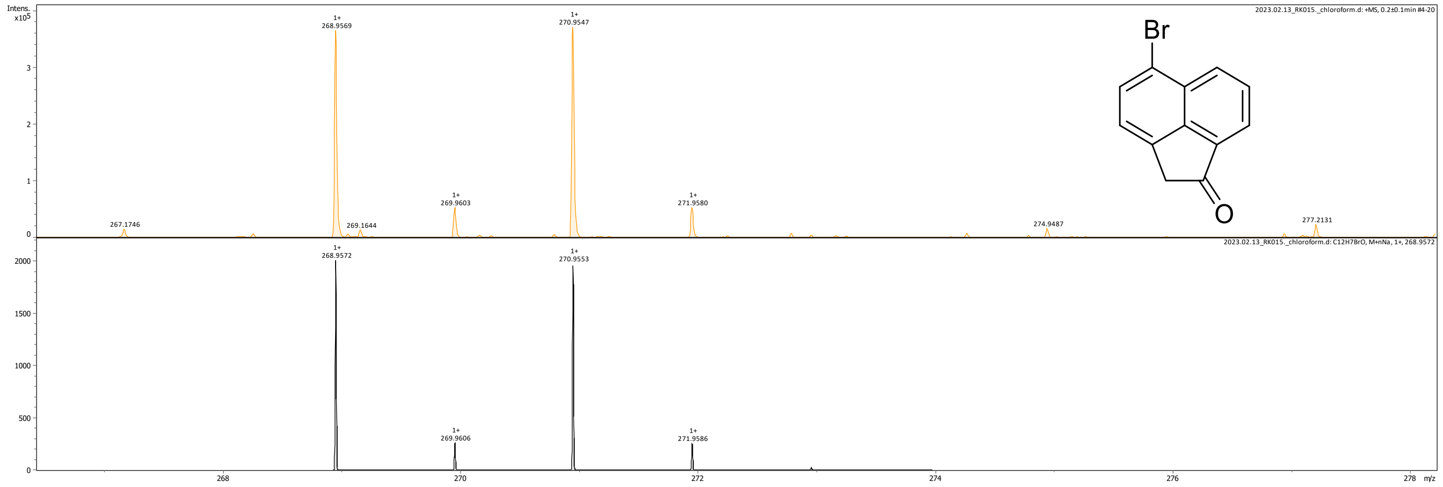

Figure S42. High resolution mass spectrum of **3** (ESI–TOF, top: experimental, bottom: simulated).

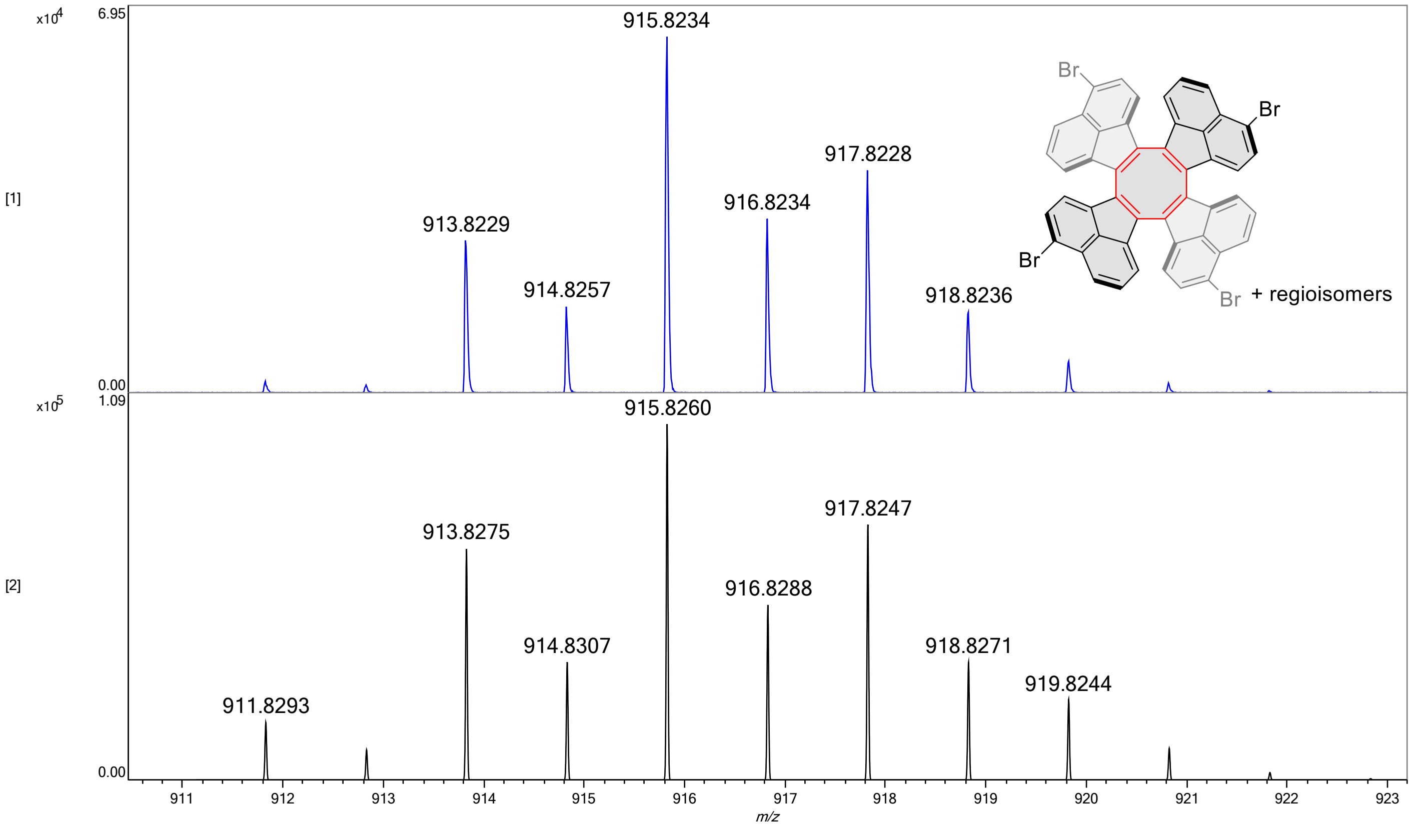

Figure S43. High resolution mass spectrum of **4** (MALDI–TOF, top: experimental, bottom: simulated).

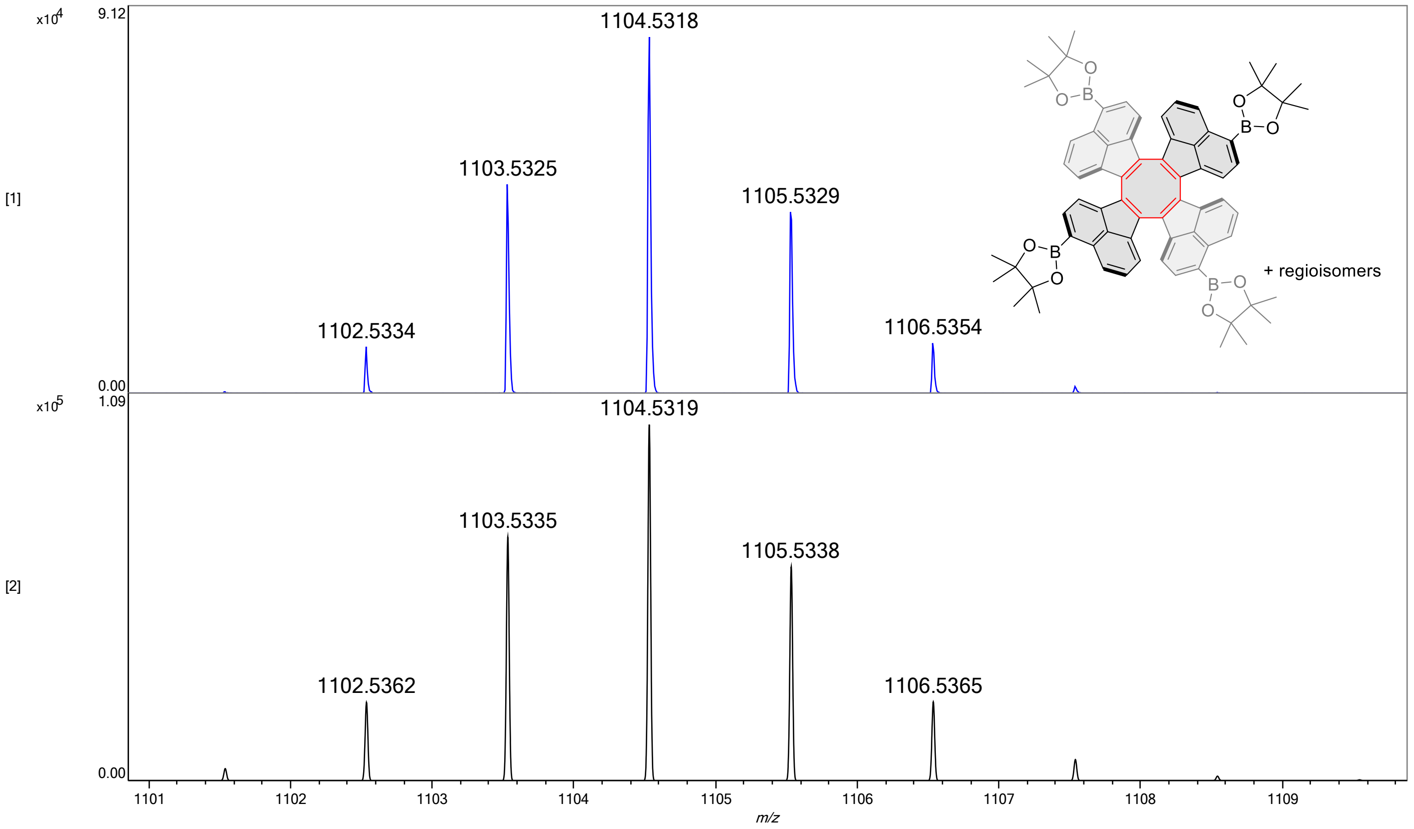

Figure S44. High resolution mass spectrum of **5** (MALDI–TOF, top: experimental, bottom: simulated).

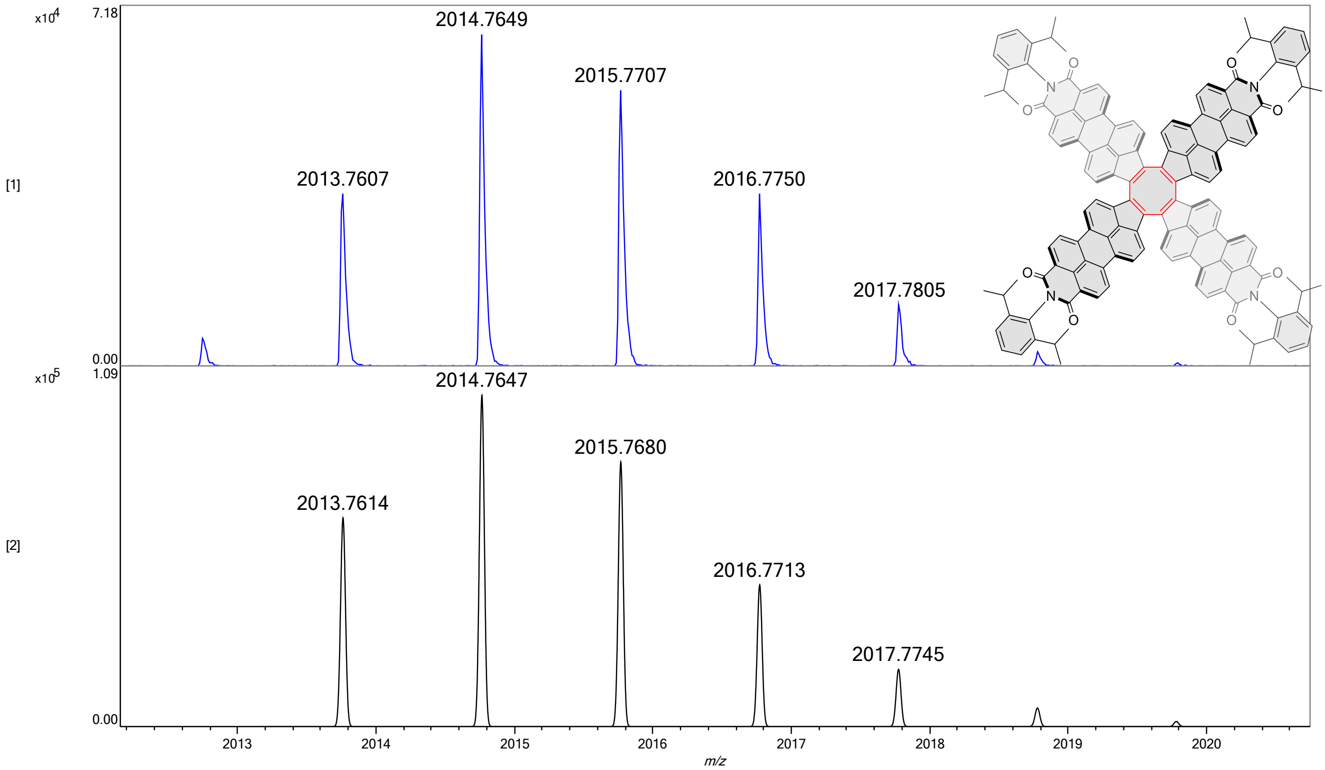

Figure S45. High resolution mass spectrum of **2** (MALDI–TOF, top: experimental, bottom: simulated).

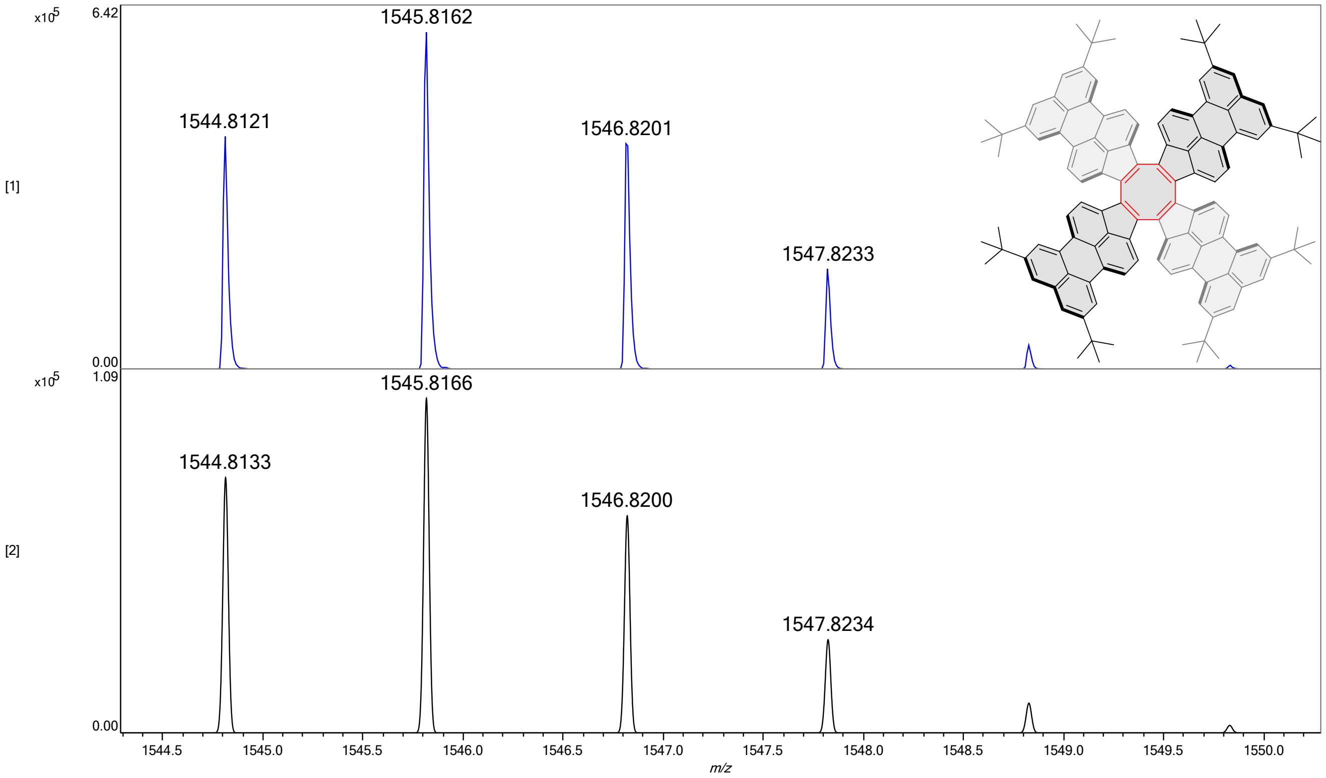

Figure S46. High resolution mass spectrum of **1** (MALDI–TOF, top: experimental, bottom: simulated).

References

(1) Frisch, M. J.; Trucks, G. W.; Schlegel, H. B.; Scuseria, G. E.; Robb, M. A.; Cheeseman, J. R.; Scalmani, G.; Barone, V.; Petersson, G. A.; Nakatsuji, H.; Li, X.; Caricato, M.; Izmaylov, A. F.; Zheng, G.; Sonnenberg, J. L.; Hada, M.; Ehara, M.; Toyota, K.; Fukuda, R.; Hasegawa, J.; Ishida, M.; Nakajima, T.; Honda, Y.; Kitao, O.; Nakai, H.; Vreven, T.; Montgomery, Jr., J. A.; Peralta, J. E.; Ogliaro, F.; Bearpark, M.; Heyd, J. J.; Brothers, E.; Kudin, K. N.; Staroverov, V. N.; Kobayashi, R.; Normand, J.; Raghavachari, K.; Rendell, A.; Burant, J. C.; Iyengar, S. S.; Tomasi, J.; Cossi, M.; Millam, J. M.; Klene, M.; Adamo, C.; Gomperts, R.; Stratmann, R. E.; Yazyev, O.; Austin, A. J.; Cammi, R.; Pomelli, C.; Ochterski, J. W.; Martin, R. L.; Morokuma, K.; Zakrzewski, V. G.; Voth, G. A.; Salvador, P.; Dannenberg, J. J.; Dapprich, S.; Daniels, A. D.; Farkas, O.; Foresman, J. B.; Fox, D. J. Gaussian 16, Revision B.01, 2016.

(2) Becke, A. D. Density-Functional Exchange-Energy Approximation with Correct Asymptotic Behavior. *Phys. Rev. A* **1988**, *38* (6), 3098–3100.

(3) Becke, A. D. Density-functional Thermochemistry. III. The Role of Exact Exchange. *J. Chem. Phys.* **1993**, *98* (7), 5648–5652. https://doi.org/10.1063/1.464913.

(4) Lee, C.; Yang, W.; Parr, R. G. Development of the Colle-Salvetti Correlation-Energy Formula into a Functional of the Electron Density. *Phys. Rev. B* **1988**, *37* (2), 785–789. https://doi.org/10.1103/PhysRevB.37.785.

(5) Yanai, T.; Tew, D. P.; Handy, N. C. A New Hybrid Exchange–Correlation Functional Using the Coulomb-Attenuating Method (CAM-B3LYP). *Chem. Phys. Lett.* **2004**, *393* (1–3), 51–57. https://doi.org/10.1016/j.cplett.2004.06.011.

(6) Grimme, S.; Ehrlich, S.; Goerigk, L. Effect of the Damping Function in Dispersion Corrected Density Functional Theory. *J. Comput. Chem.* **2011**, *32* (7), 1456–1465. https://doi.org/10.1002/jcc.21759.

(7) Chen, Z.; Wannere, C. S.; Corminboeuf, C.; Puchta, R.; Schleyer, P. von R. Nucleus-Independent Chemical Shifts (NICS) as an Aromaticity Criterion. *Chem. Rev.* **2005**, *105* (10), 3842–3888. https://doi.org/10.1021/cr030088+.

(8) Regulska, E.; Ruppert, H.; Rominger, F.; Romero-Nieto, C. Synthesis of Blue-Luminescent Seven-Membered Phosphorus Heterocycles. *J. Org. Chem.* **2020**, *85* (2), 1247–1252. https://doi.org/10.1021/acs.joc.9b02723.

(9) Niyas, M. A.; Ramakrishnan, R.; Vijay, V.; Sebastian, E.; Hariharan, M. Anomalous Halogen–Halogen Interaction Assists Radial Chromophoric Assembly. *J. Am. Chem. Soc.* **2019**, *141* (11), 4536–4540. https://doi.org/10.1021/jacs.8b13754.

(10) Chiarucci, M.; Mazzanti, A.; Righi, P.; Bencivenni, G.; Mancinelli, M. Noncovalent Interactions between Stacked Arenes in 1,8-Bis-(1-Naphthyl)-Naphthalenes. *Eur. J. Org. Chem.* **2021**, *2021* (18), 2594–2603. https://doi.org/10.1002/ejoc.202100044.

(11) Reddy Kotla, S.; Kakumanu, S.; Williams, D.; Kharel, K.; Günaydın-Şen, Ö.; Mague, J. T.; Chandrasekaran, P. Synthesis and Characterization of an Acenaphthene–Fused, π-Extended Tetrathiafulvalene Derivative. *Synth. Met.* **2018**, *242*, 49–54. https://doi.org/10.1016/j.synthmet.2018.05.003.
